# Prolonged β2-agonist treatment enhances muscle-specific glucose uptake in individuals with overweight and obesity: a randomized placebo-controlled trial

**DOI:** 10.1038/s41467-026-71897-9

**Published:** 2026-04-21

**Authors:** Pip M. G. Van Lier, Tineke van de Weijer, Froukje Vanweert, Kim Brouwers, Kevin M. R. Nijssen, Gert Schaart, Esther Moonen-Kornips, Sten M. M. van Beek, Sam Springer, Roel Wierts, Peter J. Joris, Vera B. Schrauwen-Hinderling, Esther Phielix, Tore Bengtsson, Patrick Schrauwen, Joris Hoeks

**Affiliations:** 1 Department of Nutrition and Movement Sciences, NUTRIM Institute of Nutrition and Translational Research in Metabolism, Maastricht University, Maastricht, the Netherlands; 2 Department of Radiology and Nuclear Medicine, MUMC+, Maastricht, the Netherlands; 3 Department of Human Biology, NUTRIM Institute of Nutrition and Translational Research in Metabolism, Maastricht University, Maastricht, the Netherlands; 4 Institute for Clinical Diabetology, German Diabetes Center, Leibniz Institute for Diabetes Research at Heinrich Heine University Düsseldorf, Düsseldorf, Germany; 5 German Center for Diabetes Research (DZD), Partner Düsseldorf, Neuherberg, Germany; 6 Department of Molecular Biosciences, The Wenner-Gren Institute, Stockholm University, Stockholm, Sweden; 7 Clinical Epidemiology, Leiden University Medical Center, Leiden, the Netherlands

**Keywords:** Type 2 diabetes, Obesity

## Abstract

Impaired post-prandial skeletal muscle glucose uptake plays a pivotal role in the development of type 2 diabetes mellitus (T2DM), yet pharmacological strategies to enhance muscle glucose uptake are limited. Previous (pre)clinical research revealed that β_2_-adrenergic receptor (β2-AR) stimulation enhances glucose uptake, but its clinical relevance in individuals susceptible to developing T2DM is unknown. Here we determined in a double-blinded, placebo-controlled, crossover study (ClinicalTrials.gov-identifier: NCT04921306), the effects of a 4-week treatment with the β_2_-adrenergic agonist clenbuterol (40 μg/day) on insulin-stimulated glucose uptake in the quadriceps muscle (primary outcome) and brown adipose tissue (BAT) (secondary outcome) using ^18^F-FDG PET-MRI during a hyperinsulinemic-euglycemic clamp in individuals with overweight or obesity (age: 40-70 years, BMI: 25-35 kg/m^2^). A total of 14 participants were recruited and randomized. Insulin-stimulated glucose uptake tended to improve in vastus lateralis (15%, p = 0.072) and increased significantly in the hamstring (13%, p = 0.039) muscle, while BAT uptake (p = 0.720) remained unaffected. These findings suggest potential therapeutic benefits of β_2_-AR stimulation for improving muscle-specific glucose uptake in individuals with or at risk for developing diabetes.

## Abstract (web summary)

The study showed in a placebo-controlled crossover design that 4-week treatment with β2-adrenergic agonist clenbuterol enhances insulin-stimulated glucose uptake in skeletal muscle but not brown adipose tissue in individuals with obesity.

## Introduction

Type 2 diabetes mellitus (T2DM) is a rapidly growing public health problem currently affecting over 530 million people worldwide^1^. In healthy individuals, skeletal muscle plays a crucial role in maintaining glucose homeostasis, as postprandial, insulin-stimulated glucose uptake by skeletal muscle accounts for approximately 80% of total glucose disposal^2–5^. Hence, it is not surprising that insulin resistance in skeletal muscle, which impairs its ability to take up glucose after a meal, is a key feature in the development of T2DM. Nevertheless, pharmacological options targeting muscle-specific glucose uptake remain underdeveloped.

In preclinical models it has been demonstrated that β_2_-adrenergic receptor (β_2_-AR) agonists enhance glucose uptake in skeletal muscle via the mTORC2 pathway^6,7^. Indeed, treatment of L6 muscle cells with the β_2_-AR agonist zinterol increased glucose uptake in vitro^8,9^. In line with these in vitro findings, C56Bl/6N diet-induced obese mice also showed a 74% improvement in basal skeletal muscle glucose uptake after 6 days of low-dose treatment with the selective β_2_-AR agonist clenbuterol^10^. In addition, other in vivo studies with obese Zucker rats reported robust improvements in glucose and insulin tolerance upon prolonged β_2_-agonist treatment^11–14^. While these preclinical findings are promising, the translational potential to humans—particularly those at risk for developing T2DM—remains unclear. Clinical studies by our group^15^ and others^16,17^ have demonstrated an improved glucose homeostasis and peripheral insulin sensitivity following β_2_-AR agonist treatment—such as terbutaline sulfate (4 weeks) or clenbuterol (2 weeks)—in healthy, young volunteers. However, as these studies were conducted in young, healthy males, the clinical implications for individuals with disturbed glucose homeostasis remain unknown. Thus, whether stimulation of the β_2_-adrenergic pathway can improve muscle-specific glucose uptake in an at risk population, such as individuals with overweight or obesity who are more susceptible to developing T2DM, needs to be investigated.

In addition to skeletal muscle glucose uptake, brown adipose tissue (BAT) activation provides a potential secondary mechanism through which prolonged β_2_-AR stimulation could augment glucose homeostasis. BAT has been described as a metabolic sink for glucose due to its high thermogenic capacity, rendering it as an interesting tissue to improve glucose homeostasis^18^. Although BAT was previously believed to be primarily activated by the β_3_-AR, recent evidence in healthy volunteers suggests a pivotal role for the β_2_-AR in stimulating glucose uptake in vivo in human BAT, as quantified by ^18^F-fluorodeoxyglucose (FDG) uptake^19,20^, thereby opening potential alternative avenues to combat T2DM. Although promising, it is currently unknown whether prolonged β_2_-AR stimulation is able to enhance in vivo BAT activity, and thereby improve glucose homeostasis, in a population at risk for developing T2DM.

To investigate whether β_2_-AR stimulation can improve tissue-specific clearance of glucose in skeletal muscle and BAT, and augment insulin sensitivity in individuals susceptible to developing T2DM, we conducted a randomized, double-blinded, placebo-controlled, cross-over study. This study examined the effects of a 4-week clenbuterol treatment (40 μg/day) on tissue-specific insulin-stimulated glucose uptake in skeletal muscle and BAT as reflected by metabolic rate of glucose (MRgluc) and mean standardized uptake value (SUV), respectively, in volunteers with overweight or obesity using a hyperinsulinemic-euglycaemic clamp combined with dynamic ^18^F-FDG positron emission tomography (PET)-magnetic resonance imaging (PET-MRI).

## Results

### Participant characteristics

Fourteen healthy male (*n* = 11) and postmenopausal female (*n* = 3) volunteers (age: 62 ± 8.2 years, BMI 28.6 ± 2.2 kg/m^2^) completed a randomized, double-blinded, placebo-controlled, crossover study (Table 1). No drop-outs were reported, and medication compliance rate was above 98% for both clenbuterol and placebo groups. Ten participants reported one or more side effects. In the clenbuterol period, three participants reported hand tremors, while two participants experienced headaches and an increased heart rate or palpitations. Muscle cramps were also noted by two participants, and one participant reported restlessness, feeling of anxiety, and nausea during this period. In the placebo period, three participants reported hand tremors, and one participant experienced precarious balance, restlessness, and headaches. All side effects were mild in nature. During the clenbuterol period, reported side effects were resolved after discontinuation of the treatment, while those reported during the placebo period were transient and not related to active drug effects. Two serious adverse events (subcutaneous infusion of glucose during a clamp and thrombosis in the lower extremity) were reported during the placebo period, which were unrelated to the study medication. No carryover effect was detected between treatment and period for the primary outcome, indicating that the washout period was adequate.

**Table 1:** Subject characteristics at screening

Parameters	Mean ± SD
Age (years)	62 ± 8.2
Sex (female | male)	3 | 11
Body weight (kg)	86.8 ± 10.7
Height (cm)	174.2 ± 8.4
BMI (kg/m^2^)	28.6 ± 2.2
Overweight (*n* = 9) | Obesity class I (*n* = 4)	27.4 ± 1.4 | 31.4 ± 0.9
Blood parameters:	
Potassium (mmol/L)	4.5 ± 0.4
ALAT (U/L)	27.9 ± 13.6
ASAT (U/L)	23.4 ± 8.5
y-GT (U/L)	28.5 ± 15.5
TSH (mU/L)	2.5 ± 1.4
Hemoglobin (mmol/L)	9.4 ± 0.9
Creatinine (µmol/L)	77.0 ± 24.5
eGRF (mL/min/1.73 m)	82.3 ± 9.7

Data are presented as mean ± standard deviation for *n*  =  14.

*BMI* body mass index, *ALAT* alanine transaminase, *ASAT* aspartate aminotransferase, *γ-GT* gamma-glutamyl transferase, *eGRF* estimated glomerular filtration rate, *TSH* thyroid-stimulating hormone.

### Clenbuterol increases insulin-stimulated glucose uptake in skeletal muscle

**Fig. 1:**
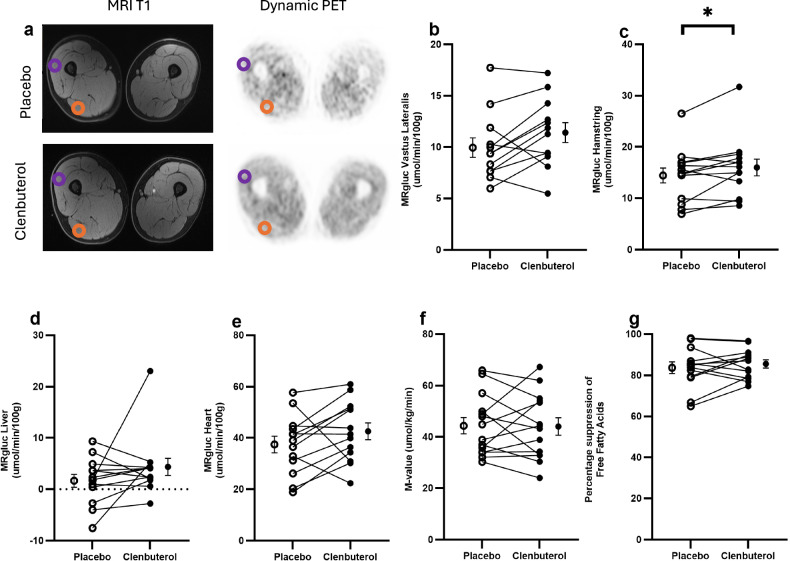
Insulin-stimulated skeletal muscle glucose uptake was higher after prolonged clenbuterol treatment. Insulin-stimulated metabolic rate of glucose (MRgluc) in skeletal muscle was measured by means of dynamic ^18^F-FDG PET-MRI during the hyperinsulinemic-euglycemic clamp. Representative MRI and PET images depicting the regions of interest (ROIs), drawn within the belly of the vastus lateralis (purple) and m. biceps femoris of the hamstring (orange) muscles of one participant upon clenbuterol vs. placebo (**a**). From these ROIs, the tissue-specific MRgluc (μmol/min/100 g) was computed in b-e: Vastus Lateralis muscle, t-test = −1.47, 95% CI: −3.09 to 0.15; *p* = 0.072, Cohen’s *d* = −0.575 (**b**), Hamstring muscle, t-test = −1.57, 95% CI: −3.03 to −0.11; *p* = 0.039, Cohen’s *d* = −0.649 (**c**), Liver (**d**) and Heart (e). The whole-body insulin sensitivity was assessed by calculating the M-value (umol/kg/min) during the hyperinsulinemic-eurglycemic clamp (**f**). The insulin-induced suppression of plasma free fatty acid concentrations, reflecting adipose tissue insulin sensitivity, was determined by assessing the plasma free fatty acids concentrations during the stable high phase of the clamp compared to baseline (**g**). All data are presented as mean ± SEM. Two-sided paired sample t-tests (for vastus lateralis (*n* = 12), heart (*n* = 13), *M*-value (*n* = 14), and percentage suppression of free fatty acids (*n* = 13)) or two-sided Wilcoxon signed ranks-test (for liver (*n* = 13) and hamstring (*n* = 13)) were used to compare differences between treatments, *p < 0.05.

**Fig. 2:**
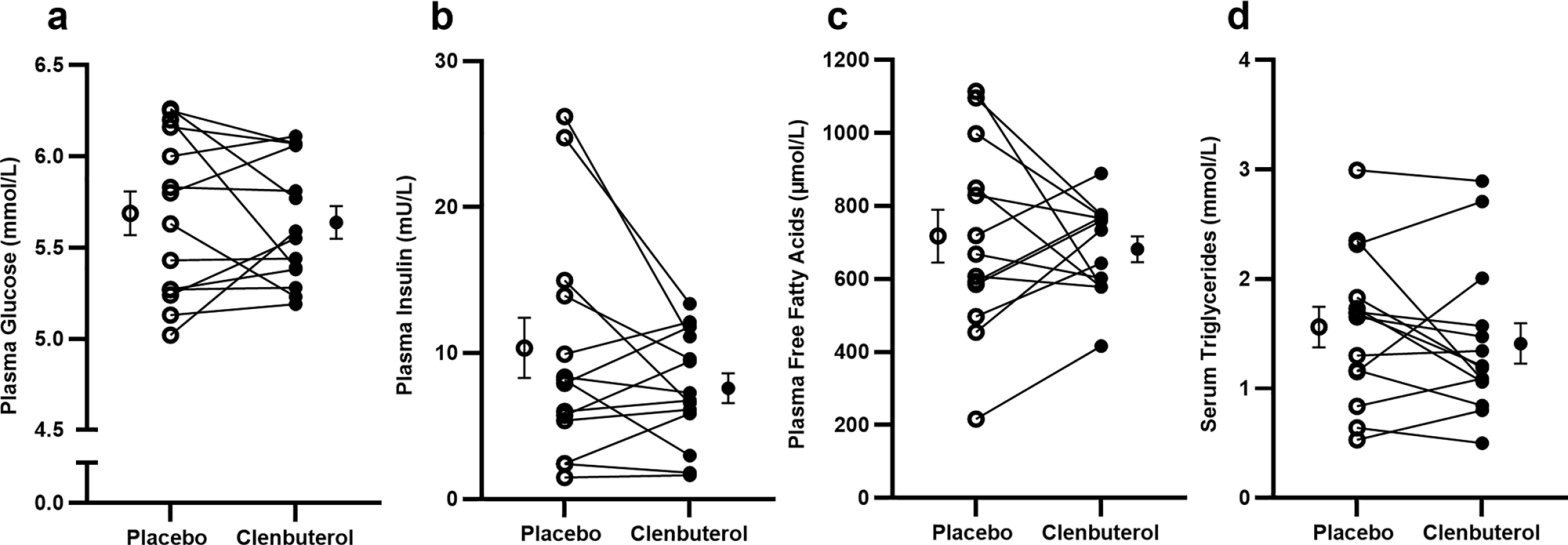
Four weeks of clenbuterol did not alter fasting plasma glucose and lipids. Plasma glucose concentration (mmol/L) (**a**), plasma insulin concentrations (mU/L) (**b**), plasma free fatty acids concentrations (µmol/L) (**c**), serum triglyceride concentrations (mmol/L) (**d**). All data are presented as mean ± SEM and statistically analyzed with two-sided paired sample t-test, only plasma insulin was analyzed with two-sided Wilcoxon signed rank test. *n* = 14 for plasma glucose, insulin, and serum triglycerides concentrations, *n* = 13 for plasma free fatty acid concentrations.

Tissue-specific insulin-stimulated glucose uptake, as reflected by MRgluc, was assessed by a hyperinsulinemic-euglycaemic clamp combined with dynamic ^18^F-FDG PET-MRI. In skeletal muscle, MRgluc was assessed in two different locations: in the vastus lateralis, the primary outcome of the study, insulin-stimulated MRgluc tended to be ~15% higher after clenbuterol vs. placebo treatment, although this did not reach statistical significance (11.43 ± 0.97 vs. 9.96 ± 0.96 μmol/min/100 g, mean difference in paired sample t-test = −1.47, 95% CI: −3.09–0.15; *p* = 0.072, Cohen’s *d* = −0.575; Fig. 1a, b). However, in the hamstring muscle, MRgluc was significantly increased (by ~13%) as compared to placebo (16.03 ± 1.63 vs. 14.46 ± 1.45 μmol/min/100 g, mean difference in paired sample t-test = −1.57, 95% CI: −3.03 to −0.11; p = 0.039, Cohen’s *d* = −0.649; Fig. 1a, c), further underlining a beneficial effect of clenbuterol on muscle-specific insulin-stimulated MRgluc. No significant changes in insulin-stimulated MRgluc were observed in the liver and heart (*p* = 0.249 and *p* = 0.127, respectively; Fig. 1d, e). Whole-body insulin sensitivity, as defined by the M-value during the hyperinsulinemic-euglycemic clamp, was similar between clenbuterol and placebo treatment (*p* = 0.926, respectively; Fig. 1f). During the high-insulin infusion phase of the clamp, neither plasma insulin concentrations (104.08 ± 7.55 μmol/kg/min vs. 105.85 ± 5.54 μmol/kg/min; *p* = 0.723, data not shown) nor C-peptide levels (1.71 ± 0.23 nmol/L vs. 1.45 ± 0.17 nmol/L; *p* = 0.103, Fig. S1) were different between placebo and clenbuterol, indicating similar insulin levels and endogenous insulin secretion under these conditions. Moreover, since adipose tissue is also an important insulin-sensitive tissue and insulin inhibits lipolysis, we determined the insulin-induced suppression of plasma free fatty acid concentrations during the clamp as a measure of adipose tissue insulin sensitivity. However, no changes in adipose tissue insulin sensitivity were observed (*p* = 0.378, Fig. 1g). Fasting plasma glucose, insulin, free fatty acids, and serum triglyceride concentrations were not altered after clenbuterol treatment compared with placebo (*p* = 0.681, *p* = 0.363, *p* = 0.693, and *p* = 0.300, respectively; Fig. 2a–d).

### Clenbuterol does not affect insulin-stimulated glucose uptake in brown adipose tissue

**Fig. 3:**
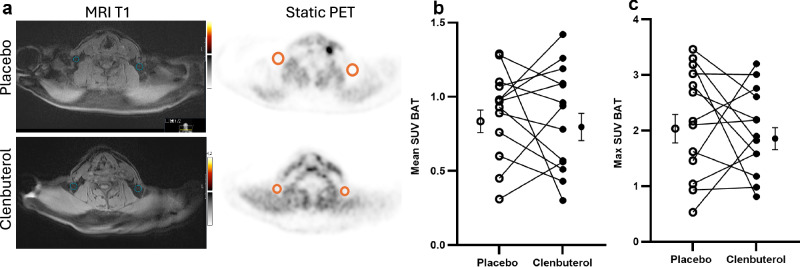
Supraclavicular adipose tissue activity was not altered after 4 weeks of clenbuterol treatment. Representative MRI and PET images of BAT activity in the supraclavicular region following placebo or clenbuterol treatment. Volumes of Interest (VOIs) with a diameter of 2 cm were placed in the supraclavicular adipose tissue area, where BAT activity could be expected (blue circle in MRI T1 image and orange circle in static PET image), during both the placebo and clenbuterol periods. In the PET image, if BAT activity were present, it would appear as a high-uptake signal (black color) in the supraclavicular regions (orange circles) on the static PET images (**a**). BAT activity was quantified using the mean standardized uptake value (SUV) (**b**) and the maximal SUV (**c**). All data are presented as mean ± SEM and statistically analyzed by means of a two-sided paired sample t-test. *n* = 13 for all figures.

Insulin-stimulated glucose uptake in the supraclavicular fat depot, measured by static ^18^F-FDG PET-MRI, was assessed as a potential indicator of BAT activity. Overall, however, we did not detect any notable ^18^F-FDG uptake in the supraclavicular fat depot in this population. The mean SUV of the supraclavicular fat depot was also not different between clenbuterol and placebo treatment and averaged 0.85 ± 0.1 vs. 0.89 ± 0.08, respectively (mean difference in paired samples t-test = 0.04, 95% CI: −0.20 to 0.28; *p* = 0.720, Cohen’s *d*  = 0.102; Fig. 3a, b). Similar results were observed for the maximal SUV in BAT, which averaged 1.99 ± 0.21 after clenbuterol and 2.18 ± 0.27 after placebo (mean difference in paired sample t-test = 0.19, 95% CI: −0.50–0.88; *p* = 0.556, Cohen’s *d*  = 0.167; Fig. 3a, c).

To assess potential refractory adrenal gland atrophy upon clenbuterol treatment, T2-weighted images were used to assess adrenal gland volumes. However, no significant differences were observed between clenbuterol and placebo, neither in the right adrenal gland volume (*p* = 0.752) nor in the left (*p* = 0.747) and mean adrenal gland volume (average of left and right adrenal glands; *p* = 0.963) (Fig. S2).

### Body composition is not affected by clenbuterol treatment

**Fig. 4:**
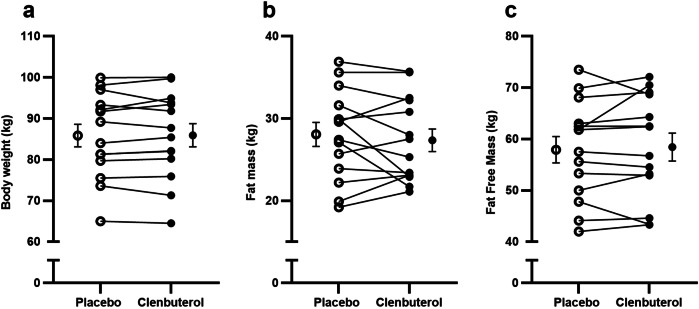
Body weight and composition were not different after 4 weeks of clenbuterol. Body weight (kg) (**a**), fat mass (kg) (**b**), fat-free mass (kg) (**c**). All data are presented as mean ± SEM and statistically analyzed with two-sided paired sample t-test. *n* = 14 for all figures.

Since (prolonged) treatment with β_2_-AR agonists—especially at high doses—has been associated with alterations in body composition, we next investigated the effect of clenbuterol vs. placebo treatment on body weight and fat (free) mass. Body weight was unaffected by clenbuterol treatment (*p* = 0.877) (Fig. 4a). In addition, fat mass and lean mass were also not significantly different between clenbuterol and placebo (*p* = 0.396 and *p* = 0.557, respectively) (Fig. 4b, c).

### Clenbuterol alters nocturnal protein oxidation but not energy expenditure

**Fig. 5:**
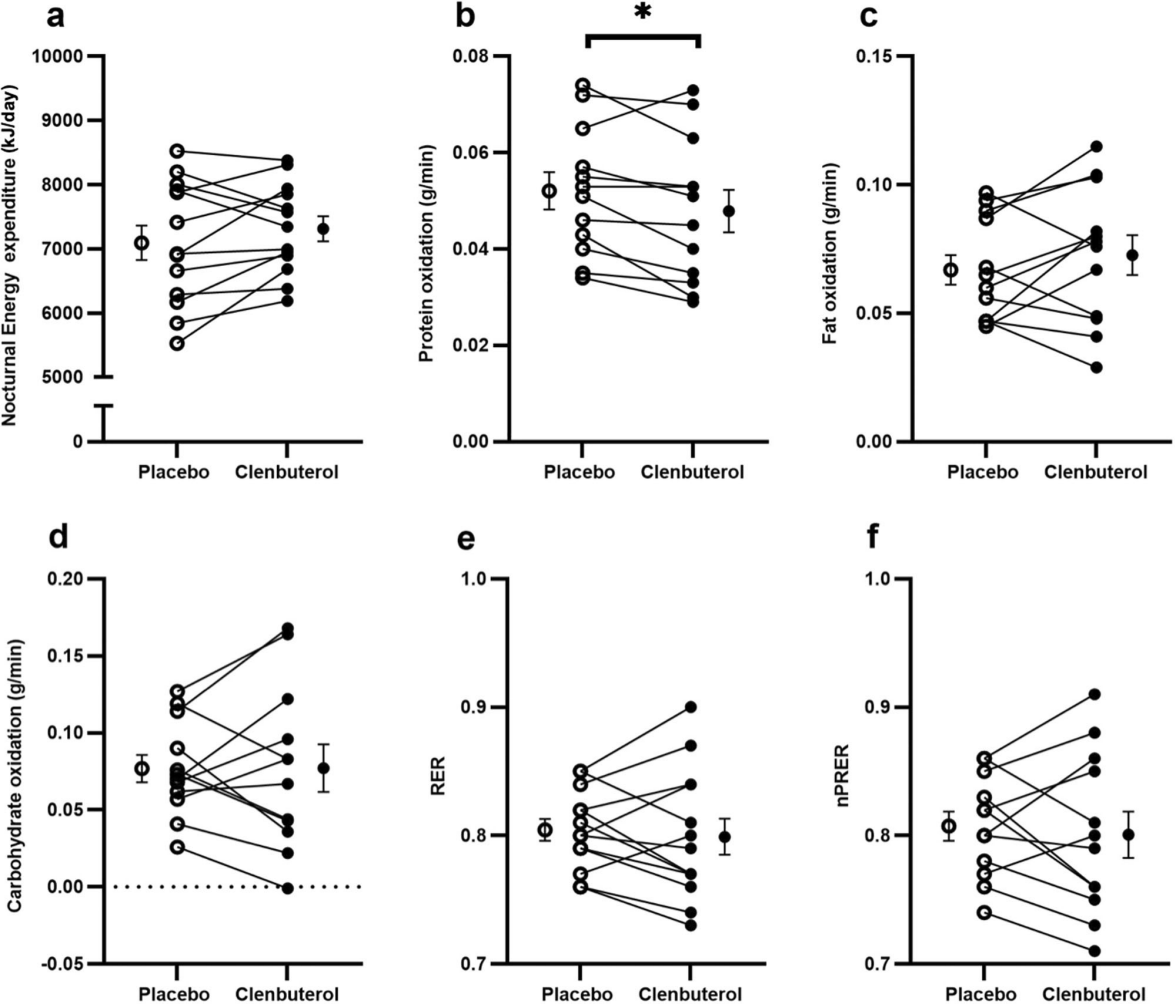
Nocturnal protein oxidation was lower after 4 weeks of clenbuterol, without alterations in energy expenditure. Nocturnal energy expenditure (kJ/min) (**a**), Nocturnal protein oxidation (g/min), *p* = 0.032 (**b**), Nocturnal fat oxidation (g/min) (**c**), Nocturnal carbohydrate oxidation (g/min) (**d**), Nocturnal respiratory exchange ratio (RER) (**e**), Nocturnal non-protein RER (nPRER) (**f**). All data are presented as mean ± SEM and statistically analyzed with two-sided paired sample t-test. *n* = 13 for RER and *n* = 12 for substrate oxidation analyses, nPRER, and nocturnal energy expenditure, *p < 0.05.

In our previous study investigating the effects of clenbuterol in healthy young males, we observed a statistically significant increase in sleeping metabolic rate without significant alterations in nocturnal substrate oxidation^15^. In the current study in individuals with overweight or obesity, clenbuterol treatment did not result in any notable increase in nocturnal energy expenditure (*p* = 0.758) (Fig. 5a). However, protein oxidation during the night was 7.9% lower after clenbuterol treatment as compared to placebo (*p* = 0.032; Fig. 5b). Nocturnal fat oxidation, carbohydrate oxidation, respiratory exchange ratio (RER), and non-protein RER (nPRER) all remained unaffected by clenbuterol (*p* = 0.271, *p* = 0.505, *p* = 0.308, and *p* = 0.388, respectively; Fig. 5c–f).

### Clenbuterol affects heart rate and blood pressure but not endothelial function

**Fig. 6:**
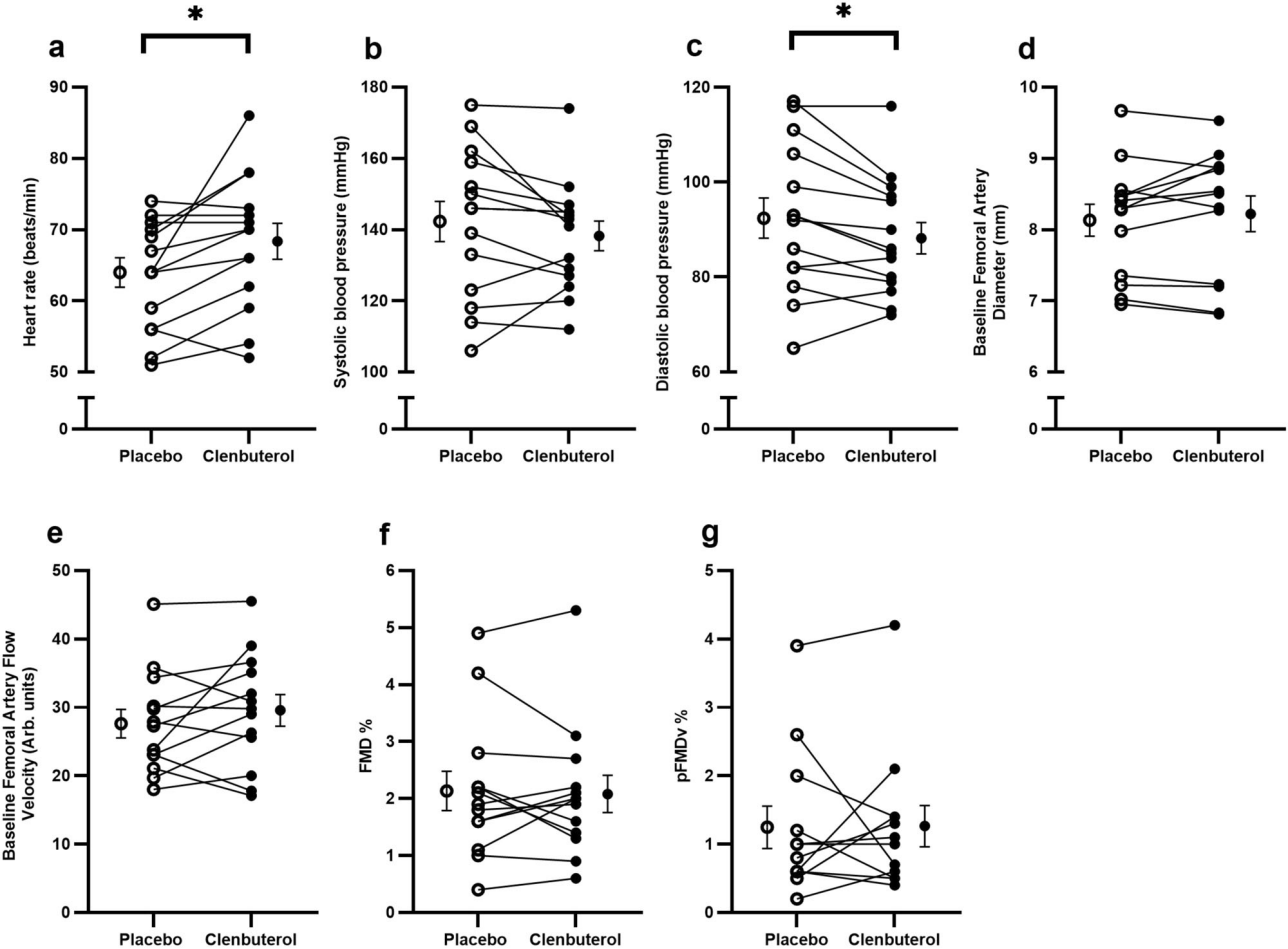
The effects of 4 weeks clenbuterol treatment on cardiovascular parameters. Heart rate (beats/min), *p* = 0.012 (**a**), systolic blood pressure (mmHg) (**b**), diastolic blood pressure (mmHg), *p* = 0.023 (**c**), baseline femoral artery diameter (mm) (**d**), basal femoral artery blood flow velocity (arb. Units) (**e**), flow-mediated dilation (FMD) (%) (**f**), flow-mediated vasodilation corrected for peak velocity flow stimulus (pFMDv) (%) (**g**). All data are presented as mean ± SEM. Two-sided paired sample t-test was used to determine differences in treatment for blood pressure (*n* = 14), heart rate (*n* = 14), and baseline femoral artery diameter (*n* = 13) and flow velocity (*n* = 13), whereas two-sided Wilcoxon signed rank test was used for FMD% (*n* = 13) and pFMDv% (*n* = 12), *p < 0.05.

Next, we measured several cardiometabolic parameters and found that heart rate was significantly elevated by ~5 beats/min after 4 weeks of clenbuterol compared to placebo (*p* = 0.012) (Fig. 6a). Although systolic blood pressure remained unaffected (*p* = 0.186, Fig. 6b), diastolic blood pressure was ~4 mmHg lower (*p* = 0.023, Fig. 6c) upon clenbuterol treatment vs. placebo. Baseline femoral artery diameter (*p* = 0.303) and blood flow velocity (*p* = 0.245) did not change, indicative of no changes in blood flow after clenbuterol treatment compared to placebo (Fig. 6d, e). Furthermore, endothelial function was not affected by clenbuterol treatment as flow-mediated dilation (FMD) responses (with and without correction for peak velocity flow stimulus; pFMDv) were similar to placebo (*p* = 0.889 and *p* = 0.859, respectively; Fig. 6f, g).

## Discussion

Stimulation of the β_2_-adrenergic pathway is a potential new strategy to alleviate insulin resistance and T2DM due to the ascribed beneficial impact on skeletal muscle glucose uptake and whole-body glucose homeostasis. Despite considerable (pre)clinical evidence derived from animal models and studies in healthy, young volunteers, the effects of β_2_-adrenergic stimulation on metabolic outcomes in a population at risk for the development of T2DM, i.e., individuals with overweight or obesity, remain unclear. In the current study, we assessed the effects of prolonged treatment with the β_2_-AR agonist clenbuterol (40 µg/day for 28 days) on tissue specific insulin-stimulated glucose uptake, as reflected by metabolic rate of glucose (MRgluc), in skeletal muscle (primary outcome) and BAT glucose uptake (secondary outcome) in individuals with overweight and obesity, using the state-of-the-art ^18^F-FDG PET-MRI technique combined with hyperinsulinemic-euglycemic clamp. We found that 4 weeks of clenbuterol treatment tended to enhance insulin-stimulated MRgluc in the vastus lateralis and significantly improved in the hamstring muscle, while BAT glucose uptake remained unchanged.

Preclinical studies have demonstrated that stimulation with β_2_-AR agonists improves skeletal muscle insulin sensitivity and glucose uptake, potentially via the mTORC2 pathway^6,7,10–12,21^, thereby highlighting the β_2_-AR pathway as an alternative antidiabetic target. This is further supported by previous clinical research demonstrating that β_2_-AR agonist treatment can markedly increase whole-body glucose homeostasis by 13–45% in young, healthy humans, depending on the specific agonist used, dose and treatment duration^15–17^. In the current study, we could demonstrate that 4-week treatment with the β_2_-AR agonist clenbuterol (40 μg/day) resulted in an enhanced muscle-specific MRgluc in individuals with overweight and/or obesity, thereby underscoring the clinical relevance of the β_2_-AR pathway. Specifically, glucose uptake in the hamstring muscle increased significantly by 13% following treatment with clenbuterol, while glucose uptake in the vastus lateralis muscle tended to improve by 15%, highlighting a beneficial effect of clenbuterol on insulin-stimulated MRgluc.

Treatment with β_2_-AR agonists might augment skeletal muscle glucose uptake by increasing peripheral and microvascular blood flow through vasodilation, as previously suggested^15,22–24^. Nevertheless, we here did not observe significant changes in femoral artery blood flow velocity, suggesting that the effects on skeletal muscle glucose uptake occurred independent of changes in tissue perfusion. It is important to note, however, that femoral artery blood flow velocity was used as an indirect measure of tissue perfusion, thereby encouraging the use of specific measurements of microvascular blood flow in follow-up studies. Besides the effects on blood flow, β_2_-AR agonists are renowned for their repartitioning effects, as demonstrated in previous studies^11,12,16,25^, which could potentially augment skeletal muscle glucose uptake. However, the improvements in skeletal muscle glucose uptake observed in the present study were not attributed to changes in body weight and composition. This could potentially be related to the relatively low dose and/or short treatment duration applied in the present study, which may not have been sufficient to induce notable changes in body composition.

In contrast to previous clinical studies, which reported increases in energy expenditure following both acute^25–29^ and prolonged^15^ β_2_-AR agonist treatment, we here did not observe differences in nocturnal energy expenditure following clenbuterol treatment. Interestingly, we did observe a significant decrease in nocturnal protein oxidation. While the effects of prolonged β_2_-AR agonist treatment on protein metabolism remain largely unexplored in humans, we previously reported a significant reduction in fasting plasma concentrations of 12 amino acids, including all three branched-chain amino acids, after 2 weeks of clenbuterol treatment in healthy, lean individuals^15^. Combined, these data suggest that β_2_-AR agonist treatment may redirect proteins away from oxidation, which would fit with the reported increased incorporation of amino acids into proteins^30^, presumably in muscle tissue. Although the mechanisms underlying these changes remain unclear, our findings are consistent with the effects of β_2_-AR agonists on whole-body protein turnover, also in individuals with overweight or obesity.

Despite the observed effects of clenbuterol on insulin-stimulated MRgluc in skeletal muscle, clenbuterol treatment did not affect whole-body insulin sensitivity, fasting plasma glucose or fasting insulin concentrations. It has been shown that hepatic glucose output is enhanced following β-adrenergic stimulation^31^, which could have counteracted the increased muscle glucose uptake observed in the current study. Unfortunately, in the current study, hepatic insulin sensitivity and glucose output were not examined; however, hepatic MRgluc measured during the hyperinsulinemic-euglycemic clamp using ^18^F-FDG PET-MRI, another aspect of hepatic insulin sensitivity, was not altered upon clenbuterol treatment. Nevertheless, modest alterations in hepatic glucose output or possibly in adipose tissue glucose and lipid fluxes cannot be excluded and could have offset the beneficial effects on skeletal muscle MRgluc, resulting in unchanged whole-body insulin sensitivity. Despite the lack of changes in whole-body insulin sensitivity, the increase in muscle glucose uptake observed in the current study is an important finding, as skeletal muscle accounts for the majority of post-prandial glucose uptake^32^, and enhancing skeletal muscle glucose uptake—for example, through acute exercise—is known to have beneficial health effects. However, longer-term studies will be needed to investigate whether β2-adrenergic stimulation of muscle glucose uptake results in clinical benefits on the long term.

Besides impacting skeletal muscle, β_2_-AR agonists have also been shown to enhance BAT activity and glucose uptake in vivo in healthy, lean volunteers^19,20,33^. However, whether β_2_-AR treatment also increases BAT glucose uptake in individuals with overweight or obesity has thus far not been investigated. Since insulin has been shown to stimulate glucose uptake in BAT^34^, we measured insulin-stimulated ^18^F-FDG uptake (standardized uptake values SUVmean and SUVmax) in the supraclavicular BAT region^35^ following clenbuterol treatment. However, glucose uptake in the supraclavicular area during insulin stimulation was very low in this population and we also did not observe any differences between clenbuterol treatment and placebo. Our population of individuals with overweight or obesity was defined by a high BMI, had a relatively high waist-to-hip ratio and was of older age, all of which have been shown to associate with reduced BAT depots^20,36^, which likely made detection of BAT glucose uptake challenging. Besides the supraclavicular area, other adipose tissue regions known to contain BAT, such as the paravertebral, mediastinal, and neck areas, showed no visual increase in glucose uptake. Although clenbuterol has a long half-life, our participants consumed their last clenbuterol capsule the evening before the insulin-stimulated ^18^F-FDG uptake measurement, and its residual effects may have been insufficient to effectively stimulate BAT glucose uptake. Future studies should incorporate an acute β-adrenergic stimulus to assess the long-term effects of β_2_-AR agonist treatment on BAT glucose uptake and activity in individuals with overweight or obesity.

The current proof-of-principle study highlights that stimulation of the β_2_-adrenergic pathway provides an interesting alternative therapeutic avenue to improve glucose homeostasis by augmenting skeletal muscle insulin-stimulated glucose uptake. Obviously, clenbuterol itself is not suitable for long-term treatment or clinical studies in people with or at risk for T2DM due to its cardiovascular side effects, which is why we also excluded these patients from the current study and focused on relatively healthy individuals with overweight or obesity. As anticipated, we observed significant elevations in heart rate, alongside a decrease in diastolic blood pressure upon clenbuterol treatment in the current study, albeit that all values remained within clinically acceptable ranges. Although these cardiovascular effects underscore that the β-adenergic pathway was indeed activated in the current study, they also highlight the necessity for the development of novel, highly selective β_2_-AR agonists with a more neutral cardiovascular risk profile in order to exploit the muscle-specific glucose uptake benefits and to achieve benefits on clinical endpoints. In this context, a recent report highlighted the development of GRK2-biased β₂-agonists, able to selectively activate the intracellular GRK2 pathway, thereby enhancing skeletal muscle glucose uptake while limiting intracellular cAMP production and hence potentially minimizing associated metabolic (e.g., enhanced hepatic glucose output) and cardiovascular side effects^37^. Such compounds would allow safe (long-term) studies in people with or at risk for T2DM—probably also at higher doses—which are warranted to establish the true clinical potential of this pathway.

Our study has several limitations. Firstly, we administered clenbuterol at a dose of 40 µg/day, similar to our previous study in healthy, lean participants^15^. Due to the larger volume of distribution of our volunteers, this dose likely resulted in a reduced relative efficacy of the dose per kilogram of body weight compared to lean individuals, thereby potentially attenuating the effects on insulin-stimulated skeletal muscle glucose uptake. Secondly, given the long (~35 h) half-life of clenbuterol^38^, rebound or withdrawal effects following the last dose taken in the evening prior to the study day are likely limited, but cannot be fully excluded and may influence the observed metabolic responses. Thirdly, our study included fewer females than males, which may reduce the generalizability of the results, as sex-specific differences in glucose metabolism^39^ and/or β_2_-AR agonist stimulation^40,41^ have been observed. Fourthly, the study’s complex design resulted in limited sleep duration for participants, thereby potentially masking the effects of β_2_-AR agonist treatment on nocturnal energy expenditure and substrate oxidation. Finally, the lack of direct measurements of skeletal muscle microvascular recruitment in our study limits our ability to draw definitive conclusions about the role of (micro)vascular function in the observed metabolic responses.

In conclusion, we here demonstrate the intrinsic capacity of β_2_-AR agonist treatment to stimulate muscle-specific insulin-stimulated glucose uptake, as reflected by MRgluc, in a population susceptible to developing T2DM, thereby potentially opening new therapeutic avenues to improve glucose homeostasis in individuals with prediabetes and T2DM. Hence, future studies are warranted to develop and test new β_2_-AR agonists with minimal cardiovascular side effects to evaluate their impact on glucose homeostasis in patients with T2DM, as the current study demonstrates that the mechanistic target is active and functional in an at-risk population.

## Methods

### Study participants

**Fig. 7:**
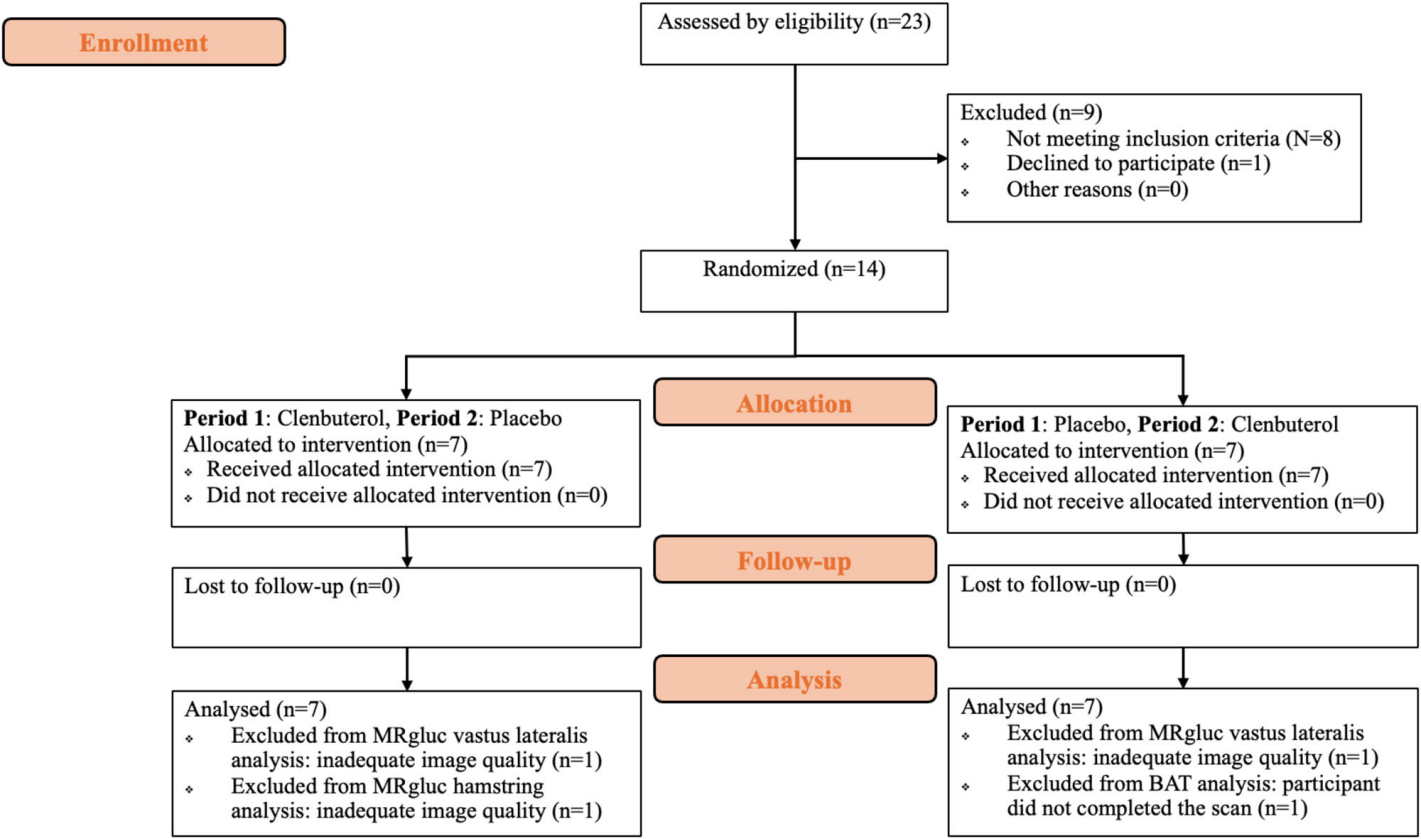
Flow diagram of participant inclusion and exclusion in the study. The CONSORT flow diagram shows the number of participants assessed for eligibility, excluded, and included in the final analysis. Reasons for exclusion at each stage are provided. Numbers indicate the number of participants (*n*) at each step.

This study included healthy, white men and (postmenopausal) women with overweight or obesity, aged 40–75 years old, and with a Body Mass Index (BMI) of 25–35 kg/m^2^. A total of 14 individuals were recruited and randomly assigned to participate in the study, with no cases of drop-out (see Fig. 7 for a CONSORT inclusion flow chart). The research data was collected at Maastricht University, Maastricht, The Netherlands, from November 1, 2022, to March 1, 2024. This study was conducted in compliance with the Declaration of Helsinki and received approval from the Medical Ethical Review Committee of Maastricht University and the university hospital (METC azM/UM) (NL76746.068.21). The research was registered prospectively at ClinicalTrials.gov on June 10, 2021, carrying the identifier NCT04921306. Prior to undergoing screening procedures, all participants gave their written informed consent.

Race, ethnicity, and sex or gender were self-reported by participants. Although both male and (postmenopausal) female participants were included in the study, we did not focus on sex- or gender-specific differences in the outcome parameters as the study assessed individual changes as compared to a between-group analysis. Participants of both sexes were included to improve the generalizability of the findings. However, the study was not powered to detect sex-specific effects, and the sample size was insufficient to support reliable stratified analyses. A full justification can be found in the Nature Portfolio Reporting Summary. The full trial protocol can be found in the Supplementary Information.

Participants were not allowed to participate in any organized or structured physical activity. Recruitment was conducted in Maastricht and its direct surroundings through flyers and online advertisements. All potential participants underwent a screening process prior to inclusion in the study. Exclusion criteria included: type 2 diabetes mellitus, cardiovascular diseases (identified through ECG, blood pressure checks, and medical histories); respiratory conditions; significant body weight fluctuations (>5 kg gain or loss in the past three months); any intention to alter body weight via diet or exercise; excessive consumption of alcohol and/or drugs; hypokalemia; hyperthyroidism; anemia; epilepsy; smoking; renal and/or liver insufficiency; and the use of any medication that could compromise the participant’s safety during the study. Participants were compensated for their time and travel related to study participation.

### Experimental design

The sample size was based on a previous study, in which ^18^F-FDG uptake was assessed in thigh muscle of healthy, overweight individuals averaged 0.0142 mL/cm^3^/min with a standard deviation of 0.0037 mL/cm^3^/min during a constant insulin infusion^42^. Assuming a physiologically relevant 20% increase in muscle glucose uptake, 80% power, and a Type I error probability of 0.05, a power calculation based on a paired sample t-test indicated that in total 14 participants were needed to complete the study.

**Fig. 8:**
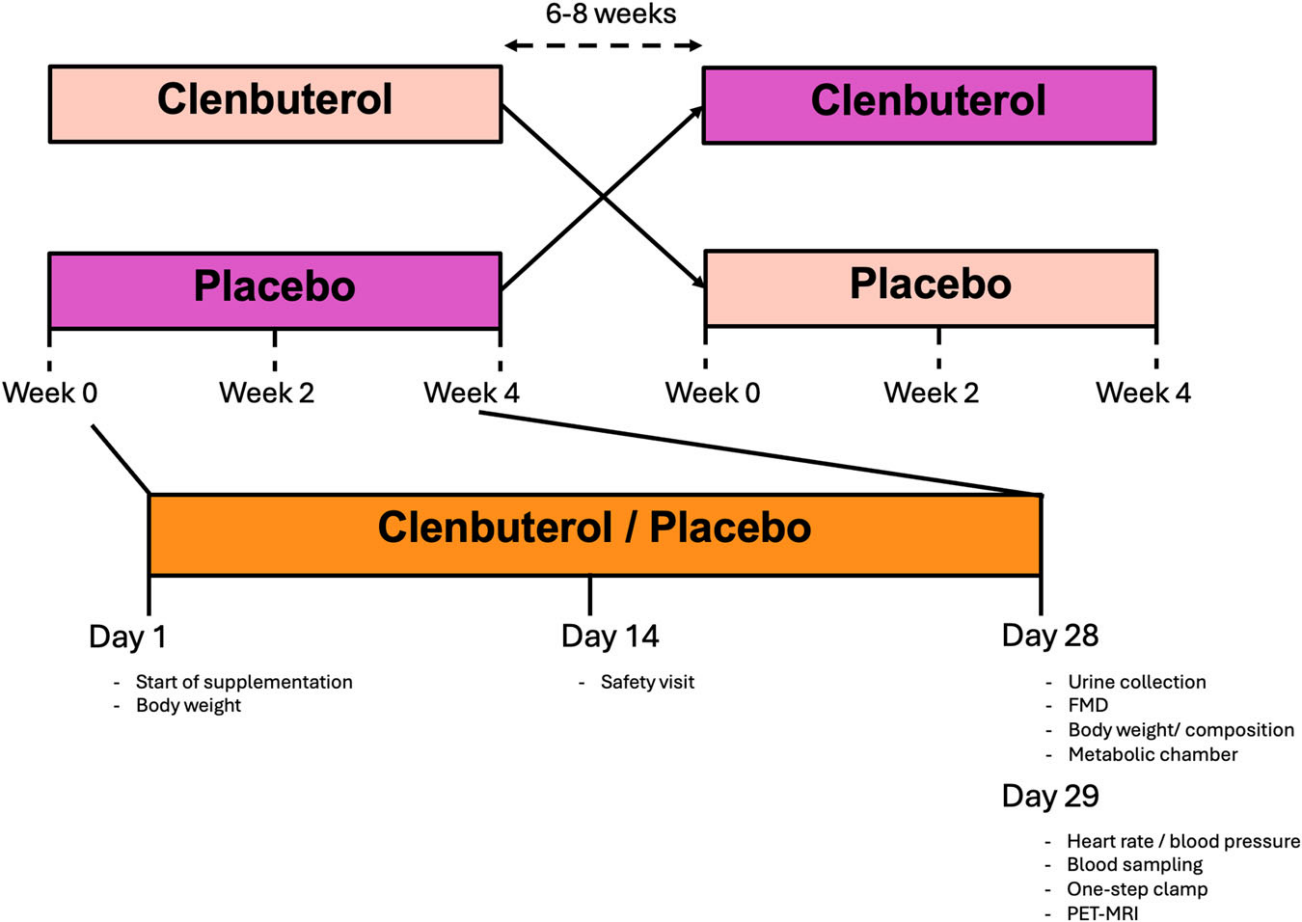
Study design. A randomized, placebo-controlled, double-blinded, cross-over design was used, in which participants received either daily clenbuterol hydrochloride (40 µg/day) or placebo treatment for 4 weeks, with a minimum wash-out period of at least 6–8 weeks. Participants were randomly allocated to one of the study arms. After 14 days of treatment, body weight, heart rate, and blood pressure were measured. After 28 days of treatment, 24-h urine was collected, blood flow and endothelial function were measured by means of flow-mediated dilation (FMD), and body composition was assessed using air displacement plethysmography (BodPod). Thereafter, participants spent the night in a respiration chamber to measure nocturnal energy expenditure and substrate utilization. The next morning, a fasted blood sample was taken, followed by a one-step hyperinsulinemic-euglycemic clamp. In the final phase of the clamp, positron emission tomography (PET)-magnetic resonance imaging (PET-MRI) was performed to assess tissue-specific insulin-stimulated ^18^F-fluorodeoxyglucose (FDG) (^18^F-FDG) uptake. All measurements conducted during the first treatment period were repeated in the second treatment period.

After screening, subjects were enrolled in a randomized, double-blind, placebo-controlled crossover study (Fig. 8). Participants were administered either clenbuterol (40 µg/day, administered as two doses of 20 µg) or a placebo for 4 weeks, before crossing over to the other arm of the study. The two treatment phases were separated by a washout period of 6–8 weeks. We opted for clenbuterol over other β_2_-agonists such as salbutamol or formoterol, given its high β_2_-selectivity, oral bioavailability and long half-life (~35 h), allowing specific and relatively stable systemic β_2_-agonist exposure over time^38,43,44^. Block randomization was performed in groups of four by an independent researcher to achieve random allocation in the study arms. Clenbuterol hydrochloride (Spiropent®) was sourced from Hikma Pharmaceuticals (Terrugem, Portugal) and, along with placebo capsules, prepared by the Pharmacy at Radboud University, Nijmegen, The Netherlands. More specifically, clenbuterol tablets were encapsulated alongside lactose, whereas placebo capsules contained only lactose. The allocation was blinded, with consecutively numbered containers. Participants were instructed to take two capsules daily, one with breakfast and one with dinner. They were also instructed to return any unused capsules and to maintain their habitual diet and physical activity levels throughout the study. The compliance rate was measured as the number of capsules returned by participants divided by the total capsules dispensed for both treatment periods. To ensure baseline measures were not influenced by recent changes, participants were asked to avoid alcohol and physical activity other than their normal daily routine for 72 h prior to these measurements.

Two weeks after starting clenbuterol treatment, participants returned to the research lab for a comprehensive safety check-up and evaluation of any potentially emerged side-effects. Body weight, heart rate, and blood pressure measurements were performed.

After 4 weeks of treatment (on day 28), participants arrived at the metabolic research unit at 17:00 to first determine the endothelial function and blood flow velocity using FMD of the femoral artery in the right leg. Subsequently, body composition was measured using air displacement plethysmography (BodPod). Around ~18:00, participants consumed the final capsule of the study period with a standardized meal. Throughout the day, urine was collected for determination of nitrogen excretion. Participants stayed overnight in a respiration chamber to measure energy expenditure and substrate oxidation. Participants woke up and got dressed at ~04:00 after which they were transported to the hospital via a wheelchair. After a 5 min rest period in supine position, heart rate and blood pressure were assessed. This was followed by the collection of a fasted blood sample. Next, a one-step hyperinsulinemic-euglycemic clamp was conducted. To determine tissue-specific insulin-stimulated MRgluc, thoracic and upper leg PET imaging was performed in the final, stable period of the hyperinsulinemic-euglycemic clamp procedure.

The primary objective of this study was to assess the effect of 4 weeks of clenbuterol versus placebo treatment on tissue-specific insulin-stimulated glucose uptake, as reflected by MRgluc, in the quadriceps muscle. The secondary objective was defined as insulin-stimulated glucose uptake in BAT, as reflected by the SUV. These outcomes were quantified using a hyperinsulinemic-euglycemic clamp combined with ^18^F-FDG PET-MRI.

### ^18^F-FDG PET-MRI to determine glucose uptake and measurement of insulin sensitivity

To assess insulin sensitivity and the impact of insulin on glucose metabolism in the skeletal muscle, heart, and liver, thoracic PET scans were performed in the final phase of an hyperinsulinemic-euglycemic clamp^45^. Outside the scanning area, insulin was administered at a rate of 40 mU/m^2^/min for 3 h before the scan to evaluate whole-body insulin sensitivity, assessed by M-value, in accordance with established protocols^45^. Plasma glucose levels were clamped at approximately 6.0–6.5 mmol/L through variable co-infusion of 20% glucose. Three hours after initiating the clamp procedure, participants—while in a steady state—were transported to the PET-MRI system on a trolley and received an intravenous injection of 1 MBq/kg of ^18^F-FDG. Over the next 60 min, regular blood samples were collected, and glucose metabolism was analyzed in the skeletal muscle (vastus lateralis, hamstring), liver, and heart using ^18^F-FDG PET-MRI imaging.

All PET-MRI measurements were performed using a 3.0T Magnetom Biograph mMR scanner (Siemens Healthineers, Erlangen, Germany) equipped with two anterior body coils. Dynamic PET scans were started immediately after the ^18^F-FDG injection, with ^18^F-FDG PET data captured in list-mode. The liver region underwent a dynamic 10-min scan, followed by the reconstruction of data into frames of 4×15 s, 4×30 s, 4×60 s, and 2×90 s. Subsequently, four static frames alternated between the thorax and the upper leg regions, each lasting 5 min. Additionally, a 10-min static scan of the head and neck was performed to identify BAT glucose uptake. All data were corrected for dead time, radioactive decay, photon attenuation, scatter, and reconstructed into a 128 × 128 matrix using a 3D-iterative ordinary Poisson ordered-subsets expectation maximization (OP-OSEM3D) algorithm (3 iterations, 21 subsets, 1.0 zoom, 2.0 mm Gaussian smoothing). A 2D T2-weighted fast spin-echo image in two planes (sagittal and axial) was performed (TR 2150/ TE 138 ms, echo train length 33, 1 average, (0.98 × 0.98) × 6.50 mm^3^) of the regions of interest. These scans were used for anatomic correlation of the findings and for semi-automatic segmentation of the adrenal glands to obtain volumetric measurements using MIMvista software (MIM 7.3.7). Finally, the data were merged with MRI images using Siemens Syngo.via Client 10.4 (x64) VB60I_HF01, Siemens syngo.via Bootstrapper 8.0 (Siemens Healthcare).

Dynamic and static images of the abdomen and upper legs were manually combined into a dynamic time series for subsequent Patlak analysis. Time-activity curves from the acquired PET data were generated by drawing volumes of interest (VOIs) on the fused dynamic PET-MRI images, focusing on the distribution of ^18^F-FDG. VOIs were defined in critical regions, including the arch of the aorta, the entire left ventricle of the myocardium, and the right half of the liver. The VOIs for the arch of the aorta and the entire left ventricle of the myocardium were manually drawn over the entire region, whereas the VOI for the right half of the liver was manually drawn as a spherical VOI with a diameter of 20 cm. Skeletal muscle MRgluc was measured using ellipsoid VOIs, which were manually placed over the vastus lateralis and hamstring, each with a diameter of 5 cm and a length of 20 cm (Fig. 1).

The tissue-specific MRgluc was computed using a two-tissue-compartment model facilitated by Patlak analysis. Plasma input functions were sourced from the aortic arch, with adjustments made for plasma glucose concentrations using PMOD software (Version 3.7, PMOD Technologies, Zurich, Switzerland). The net influx rate constant (Ki) for ^18^F-FDG was determined through graphical analysis with Patlak plots^46–48^. The MRgluc was then calculated by multiplying Ki by the plasma glucose concentration, adjusted using the Lumped Constant (LC) (LC for heart and liver: 1.0; LC for vastus lateralis and hamstring: 1.16).

The static scans of the head and neck were analyzed for BAT glucose uptake using Syngo.via software. To assess BAT glucose uptake, VOIs were first drawn around the entire supraclavicular adipose tissue area. Within this selected VOI, a sphere with a 2 cm radius was placed at the point of highest average SUV (Fig. 3), following previously described methods^49,50^. For each VOI and sphere, the measured activity concentration was adjusted for radioactive decay, total administered activity, and body weight, yielding the mean SUV (SUVmean).

The number of participants included in the analyses differs by tissue type. For skeletal muscle, one or two participants were excluded from the analysis due to inadequate image quality (e.g., noise, movement artefacts) (vastus lateralis: *n* = 12; hamstring: *n* = 13). One participant could not complete the entire scan procedure and was therefore excluded from the BAT analysis (*n* = 13). For liver (*n* = 13) and heart (*n* = 13) only one participant was excluded from the analysis due to inadequate image quality.

### Body composition

Measurements of body weight and composition were determined using air displacement plethysmography (BodPod®, COSMED, Inc., Rome, Italy), according to standard procedures^51,52^.

### Indirect whole-room calorimetry

To evaluate nocturnal energy expenditure and substrate oxidation, participants spent the night in a metabolic chamber (Omnical, IDEE, Maastricht, the Netherlands). Participants were instructed to go to sleep at 23:00 until the next morning. The nocturnal energy expenditure during the night and substrate oxidation (carbohydrates, fats, and proteins) were calculated using Brouwers’ equation^53^. In addition, participants were asked to collect their urine over a 22-h period, which was then analyzed for urinary nitrogen excretion and total volume, in order to assess protein oxidation rates. This protein oxidation rate was subsequently used to calculate the protein oxidation based on the Brouwer method^53,54^. The protein oxidation data were then used to adjust the Brouwer equations for fat and carbohydrate oxidation. RER data was excluded for one participant due to technical issues with the respiration chamber overnight (*n* = 13). For nPRER and substrate oxidation analyses, two participants were excluded due to failed urine collection, which prevented nitrogen analysis and subsequent corrections for protein, fat, carbohydrate oxidation, and nocturnal energy expenditure (*n* = 12).

### Plasma substrate analyses

During the study, blood samples were collected in EDTA, NaF, and serum tubes. From these tubes, EDTA and NaF tubes were stored on ice and further centrifuged at 4 °C for 10 min at 1300 × *g*. Serum tubes were stored at room temperature for at least 30 min to coagulate, followed by 10 min of centrifugation at 21 °C at 1300 × *g*. Blood plasma and serum were then collected and stored at −80 °C until further analysis. During analysis, plasma glucose (Horiba, Montpellier, France), free fatty acid (WAKO, Neuss, Germany) and triglyceride concentrations (Sigma, St Louis, USA) were measured in duplicate by colorimetric analysis with a Cobas Pentra C400 analyzer (Horiba, Montpellier, France). Furthermore, plasma insulin concentrations were measured in duplicate with an ELISA (Crystal Chem, Elk Grove Village, USA). To minimize variability between samples from each participant, the analysis was performed within the same analytical run. Plasma free fatty acid concentrations were determined in duplicate at baseline and during the stable high insulin phase of the hyperinsulinemic-euglycemic clamp, to determine the degree of insulin-induced suppression of lipolysis, as a measure for adipose tissue insulin sensitivity. One participant was excluded from free fatty acid analyses due to poor sample quality (*n* = 13). C-peptide concentrations were measured using a chemiluminescent immunometric assay (Immulite XPi, Siemens). One participant was excluded from the C-peptide analysis due to a missing blood sample, resulting in a final sample size of 13 (*n* = 13).

### Blood pressure and heart rate

On day 29, heart rate and blood pressure were measured three times consecutively using an automatic inflatable cuff (Omron Healthcare, Hamburg, Germany) at ~04:30.

### Femoral artery flow-mediated vasodilation

Femoral artery blood flow was evaluated using Doppler ultrasound technology (MyLabTM-Gamma, Esaote, Maastricht, The Netherlands) with a 13–4 MHz linear transducer. In this assessment, artery diameter and blood flow velocity were continuously monitored using B-mode imaging combined with Doppler techniques. For this procedure, a 3-min baseline recording was taken after placing a pneumatic cuff around the participant’s right leg. This cuff was then inflated to 200 mmHg for a duration of 5 min, followed by a deflation phase to induce a hypoxic state, with imaging continuing for another 5 min. The collected images were then processed with special MATLAB software (MyFMD 2015, developed by Professor A.P.G. Hoeks, Department of Biomedical Engineering, Maastricht University, Maastricht, the Netherlands) to determine changes in artery diameter and velocity profiles during the measurement period^55^. The primary measure was the maximum percentage increase in arterial diameter after occlusion, compared with baseline diameter. This increase, known as the FMD response, served as a non-invasive marker for endothelial function. To account for changes in blood flow velocity, the FMD response was normalized by dividing it by the percent increase in peak velocity flow after occlusion. Due to scheduling issues, FMD data is lacking for one participant (*n* = 13). For the analysis of FMD corrected for pFMDv%, one additional participant was excluded because of a failed (peak velocity flow) stimulus assessment (*n* = 12).

### Statistical analyses

Data were significantly analyzed with SPSS IBM version 28 and graphs were created in GraphPad Prism version 10.4.0. Normality was assessed using the Shapiro–Wilk test. In line with the crossover design of the study, all data (differences between clenbuterol and placebo arms) were analyzed using a parametric paired sample t-test in case of normal distribution whereas a non-parametric Wilcoxon signed-rank test was used in case of a non-normal distribution. Effect sizes of the primary and secondary outcome parameters were calculated using Cohen’s *d* for paired samples. Potential carryover effect between treatment and period was examined for the primary outcome by unpaired t-test analyses. Statistical analyses were considered significant if *p*  <  0.05. All data are presented as mean ± SEM unless stated otherwise.

### Reporting summary

Further information on research design is available in the Nature Portfolio Reporting Summary linked to this article.

## Supplementary information

Supplementary information

Reporting summary

Transparent Peer Review file

## Source data

Source data

## Data Availability

The study protocol and statistical analysis plan are available in the Supplementary Material. Source data are provided in this paper. Upon scientific request, de-identified and processed participant data can be requested from the corresponding author (j.hoeks@maastrichtuniversity.nl), with no end date, following the completion of a signed data transfer agreement. De-identified data will be shared due to participant privacy.
