# Supplementary material for: Prolonged β2-agonist treatment enhances muscle-specific glucose uptake in individuals with overweight and obesity: a randomized placebo-controlled trial
Source: Nat Commun. 2026 Apr 21;17:5483. doi: 10.1038/s41467-026-71897-9 (PMC13284196; doi:10.1038/s41467-026-71897-9)
Supplement: Supplementary file 1 — Supplementary information [file 41467_2026_71897_MOESM1_ESM.pdf]

Supplementary Data

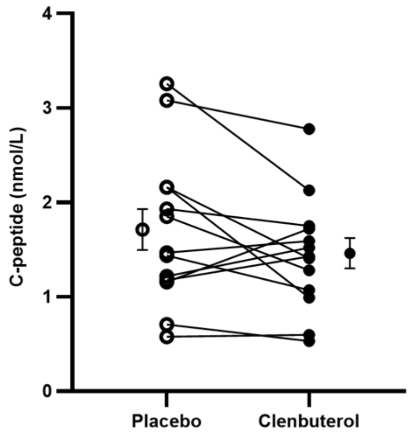

Fig S1. C-peptide was not different after four weeks of clenbuterol treatment. Data is presented as mean ± SEM and statistically analyzed with two-sided paired sample t-test. n=13.

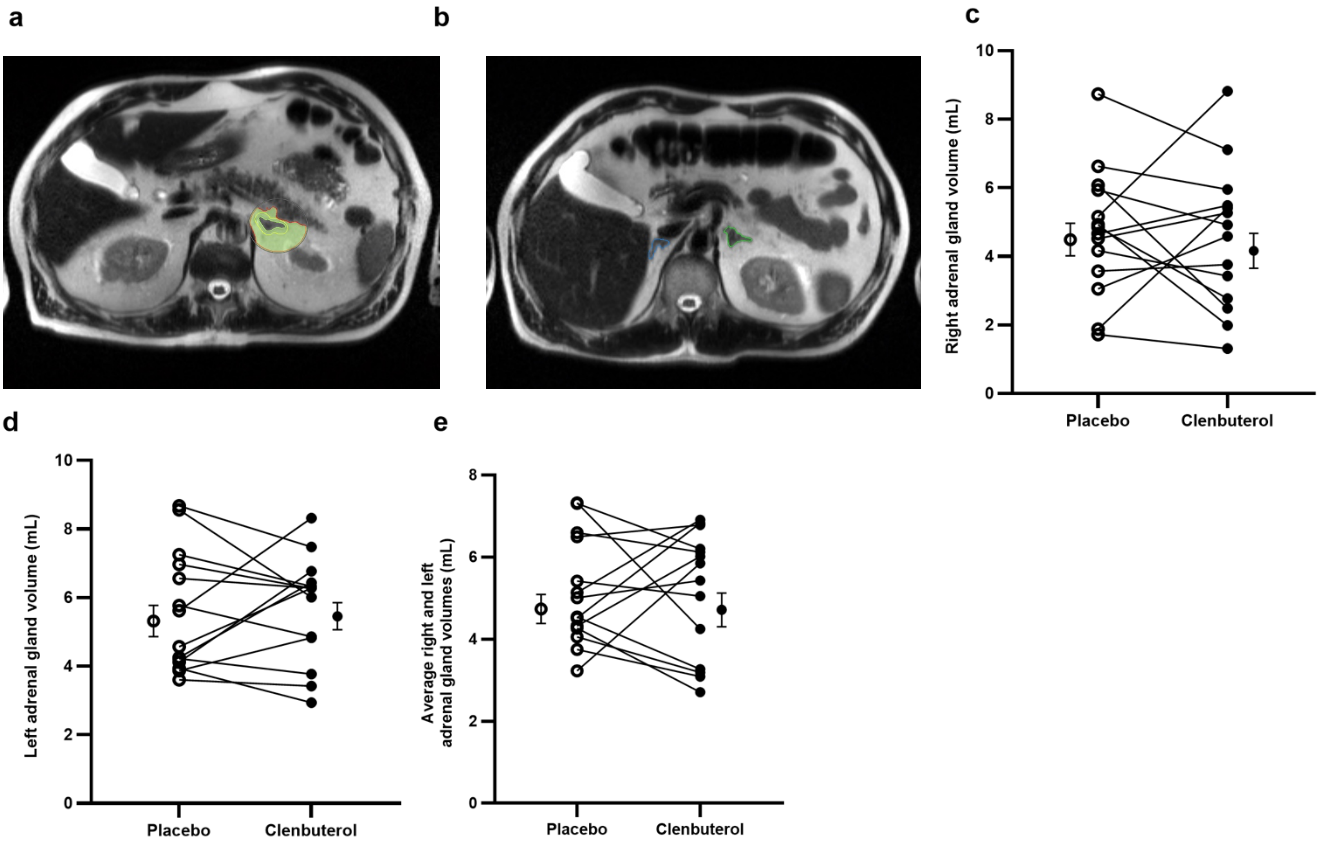

Fig S2. Adrenal gland volume was not different after four weeks of clenbuterol. A manual contour was drawn widely over the adrenal gland (yellow contour). Thereafter with a threshold tool based on intensity differences, the adipose tissue was segmented in the region of the adrenal gland (Red contour). The Adipose tissue contour was subtracted from the manually drawn contour of the adrenal gland, resulting in a more precise contour of the adrenal gland (green contour), using MIMvista software (a), The final segmentation consisted of the segmentation of the right adrenal gland (blue) and left adrenal gland (green) (b). All data are presented as mean ± SEM and statistically analyzed with two-sided paired sample t-test. n=14 for all figures.

Supplementary Material

RESEARCH PROTOCOL

Targeting the β_2-adrenergic pathway to improve skeletal muscle glucose uptake in obese humans

Investigators:

P.M.G. van Lier, MSc, Dept of Nutrition and Movement Sciences, Maastricht University, Maastricht, The Netherlands

Dr. J. Hoeks, Dept of Nutrition and Movement Sciences, Maastricht University, Maastricht, The Netherlands

(January 2023)

PROTOCOL TITLE ‘Targeting the β_2-adrenergic pathway to improve skeletal muscle glucose uptake in obese humans’

**Protocol ID**	Clenbuterol study
**Short title**	Obese human β_2-adrenergic stimulation and muscle glucose uptake
**EudraCT number**	2021-000731-31
**Version**	7
**Date**	January 2023
**Coordinating investigator/project leader**	Pip van Lier<br>Universiteitssingel 50 (room H0.366)<br>6229 ER Maastricht<br>Tel – 043 388 2124<br>E-mail: pip.vanlier@maastrichtuniversity.nl
**Principal investigator(s) (in Dutch: hoofdonderzoeker/ uitvoerder)**	Dr. Joris Hoeks<br>Universiteitssingel 50 (room 0.338)<br>6229 ER Maastricht<br>Tel – 043 3881507<br>E-mail: J.hoeks@maastrichtuniversity.nl
**Sponsor (in Dutch: verrichter/opdrachtgever)**	Maastricht University<br>PO Box 616, 6200 MD Maastricht
**Subsidising party**	The Eurostars programme
**Independent expert (s)**	Ronald Henry, MD PhD<br>Maastricht UMC+<br>Interne geneeskunde<br>Tel - 043-3871562<br>E-mail: rma.henry@mumc.nl
**Laboratory sites**	Maastricht University

	Department of Nutrition and Movement Sciences<br>Universiteitssingel 50, 6229 ER, Maastricht<br>Metabolic research unit (MRUM)<br><br>Maastricht UMC+<br>Centraal Diagnostisch Laboratorium<br>PO Box 5800, 6202 AZ Maastricht
**Pharmacy**	Apotheek Radboud Universitair Medisch Centrum<br>Postbus 9101, 6500 HB Nijmegen<br><br>Apotheek Maastricht UMC+, P. Debyelaan 25<br>6202 AZ Maastricht

PROTOCOL SIGNATURE SHEET

Name	Signature	Date
**Head of Department**<br><br>Prof. Dr. Jogchum Plat		
**[Coordinating Investigator/Project lea-der/Principal Investigator]:**<br><br>Drs. Pip van Lier<br><br>Dr. Joris Hoeks		

TABLE OF CONTENTS

1. INTRODUCTION AND RATIONALE 13
2. OBJECTIVES 16
3. STUDY DESIGN 17
4. STUDY POPULATION 22
   - 4.1 Population (base) 22
   - 4.2 Inclusion criteria 22
   - 4.3 Exclusion criteria 22
   - 4.4 Sample size calculation 23
5. TREATMENT OF SUBJECTS 25
   - 5.1 Investigational product/treatment 25
   - 5.2 Use of co-intervention (if applicable) 25
   - 5.3 Escape medication (if applicable) 25
6. INVESTIGATIONAL PRODUCT 26
   - 6.1 Name and description of investigational product(s) 26
   - 6.2 Summary of findings from non-clinical studies 27
   - 6.3 Summary of findings from clinical studies 27
   - 6.4 Summary of known and potential risks and benefits 27
   - 6.5 Description and justification of route of administration and dosage 28
   - 6.6 Dosages, dosage modifications and method of administration 28
   - 6.7 Preparation and labelling of Investigational Medicinal Product 29
   - 6.8 Drug accountability 29
7. NON-INVESTIGATIONAL PRODUCT 31
   - 7.1 Name and description of non-investigational product(s) 31
   - 7.2 Summary of findings from non-clinical studies 31
   - 7.3 Summary of findings from clinical studies 31
   - 7.4 Summary of known and potential risks and benefits 31
   - 7.5 Description and justification of route of administration and dosage 32
   - 7.6 Dosages, dosage modifications and method of administration 32
   - 7.7 Preparation and labelling of Non Investigational Medicinal Product 32
   - 7.8 Drug accountability 32
8. METHODS 33
   - 8.1 Study parameters/endpoints 33
     - 8.1.1 Main study parameter/endpoint 33
     - 8.1.2 Secondary study parameters/endpoints (if applicable) 33
     - 8.1.3 Other study parameters (if applicable) 33
   - 8.2 Randomisation, blinding and treatment allocation 33
   - 8.3 Study procedures 34
   - 8.4 Withdrawal of individual subjects 40
     - 8.4.1 Specific criteria for withdrawal (if applicable) 40
   - 8.5 Replacement of individual subjects after withdrawal 40
   - 8.6 Follow-up of subjects withdrawn from treatment 41

8.7 Premature termination of the study 41
9. SAFETY REPORTING 42
9.1 Temporary halt for reasons of subject safety 42
9.2 AEs, SAEs and SUSARs 42
9.2.1. Adverse events (AEs) 42
9.2.2. Serious adverse events (SAEs) 42
9.2.3. Suspected unexpected serious adverse reactions (SUSARs) 43
9.3. Annual safety report 44
9.4. Follow-up of adverse events 44
9.5. Data Safety Monitoring Board (DSMB) / Safety Committee 44
10. STATISTICAL ANALYSIS 45
10.2. Primary study parameter(s) 45
10.3. Secondary study parameter(s) 45
10.4. Other study parameters 46
10.5. Interim analysis (if applicable) .... 46.
11. ETHICAL CONSIDERATIONS 47
11.2. Regulation statement 47
11.3. Recruitment and consent 47
11.4. Objection by minors or incapacitated subjects (if applicable) 47
11.5. Benefits and risks assessment, group relatedness 48
11.6. Compensation for injury 50
11.7. Incentives (if applicable) 50
12. ADMINISTRATIVE ASPECTS, MONITORING AND PUBLICATION 52
12.2. Handling and storage of data and documents 52
12.3. Monitoring and Quality Assurance 53
12.4. Amendments 53
12.5. Annual progress report 54
12.6. Temporary halt and (prematurely) end of study report 54
12.7. Public disclosure and publication policy 55
13. STRUCTURED RISK ANALYSIS 56
13.2. Potential issues of concern 56
13.3. Synthesis 58
14. REFERENCES 59

LIST OF ABBREVIATIONS AND RELEVANT DEFINITIONS

**ABR**	**ABR form, General Assessment and Registration form, is the application form that is required for submission to the accredited Ethics Committee (In Dutch, ABR = Algemene Beoordeling en Registratie)**
**AE**	**Adverse Event**
**AMPK**	**AMP-activated protein kinase**
**AR**	**Adverse Reaction**
**β-ARs**	**β-adrenergic receptors**
**ALAT**	**Alanine aminotransferase**
**ASAT**	**Aspartate transaminase**
**BMI**	**Body mass index**
**CA**	**Competent Authority**
**cAMP**	**Cyclic adenosine monophosphate**
**CCMO**	**Central Committee on Research Involving Human Subjects; in Dutch: Centrale Commissie Mensgebonden Onderzoek**
**CV**	**Curriculum Vitae**
**DSMB**	**Data Safety Monitoring Board**
**eCRF**	**Electronic case report form**
**ELISA**	**Enzyme-linked immunosorbent assay**
**EU**	**European Union**
**EudraCT**	**European drug regulatory affairs Clinical Trials**
**Gamma-GT**	**Gamma-glutamyltransferase**
**GCP**	**Good Clinical Practice**
**GLUT4**	**Glucose transporter 4**
**HDL**	**High-density lipoprotein**
**IB**	**Investigator's Brochure**
**IC**	**Informed Consent**
**IMP**	**Investigational Medicinal Product**
**IMPD**	**Investigational Medicinal Product Dossier**
**LDL**	**Low-density lipoprotein**
**METC**	**Medical research ethics committee (MREC); in Dutch: medisch ethische toetsing commissie (METC)**

**MRUM**	**Metabolic research centre at Maastricht University**
**mTOR**	**Mammalian target of rapamycin**
**mTORC**	**Mammalian target of rapamycin complex 2**
**NSAIDs**	**Nonsteroidal anti-inflammatory drugs**
**Rd**	**Glucose disposal rate**
**RT-qPCR**	**Real-time quantitative polymerase chain reaction**
**(S)AE**	**(Serious) Adverse Event**
**SPC**	**Summary of Product Characteristics (in Dutch: officiële productinfomatie IB1-tekst)**
**Sponsor**	**The sponsor is the party that commissions the organisation or performance of the research, for example a pharmaceutical company, academic hospital, scientific organisation or investigator. A party that provides funding for a study but does not commission it is not regarded as the sponsor, but referred to as a subsidising party.**
**SUSAR**	**Suspected Unexpected Serious Adverse Reaction**
**T2DM**	**Type 2 diabetes mellitus**
**Wbp**	**Personal Data Protection Act (in Dutch: Wet Bescherming Persoonsgevens)**
**WMO**	**Medical Research Involving Human Subjects Act (in Dutch: Wet Medisch-wetenschappelijk Onderzoek met Mensen**

SUMMARY

Rationale: Skeletal muscle insulin resistance is a primary factor underlying an impaired postprandial glucose clearance and a major hallmark in the development of type 2 diabetes mellitus (T2DM). As such, stimulation of skeletal muscle glucose uptake independent of the insulin pathway could significantly contribute to a positive disease outcome. In this context, we have recently demonstrated in pre-clinical models that skeletal muscle glucose uptake can be mediated through an alternative novel pathway involving β_2-adrenergic receptors, through activation of mTORC2. Thus, robust improvements in glucose homeostasis were observed in diabetic rodents upon treatment with the selective β_2-agonist clenbuterol, also when administered at lower doses. Furthermore, we are currently finalizing a study in young, healthy individuals which indicates that a standard dose of clenbuterol (40 μg/day) is well-tolerated by adult humans. During the current study, we will therefore investigate the effect of clenbuterol supplementation on glucose homeostasis in obese subjects to identify if targeting the β_2-adrenergic-mTORC2 pathway could alleviate insulin resistance in skeletal muscle and activate brown adipose tissue (BAT).

Objective: The primary objective is to assess whether insulin-stimulated glucose uptake in quadriceps muscle can be improved through 4 weeks of supplementation of clenbuterol. The secondary objective is to test if 4 weeks of clenbuterol treatment improves insulin-stimulated glucose uptake in BAT.

Study design: 4-week randomized, double-blinded, placebo-controlled, cross-over design with a minimum wash-out period of 6-8 weeks.

Study population: 40 overweight or obese male and (postmenopausal) women (BMI: 25-35 kg/m^2) aged between 40-75 years.

Intervention (if applicable): 4-week oral supplementation with clenbuterol hydrochloride (40 μg/day) or placebo. Capsules (20 μg) will be consumed twice daily.

Main study parameters/endpoints: Main study parameter is insulin-stimulated ^{18}F-FDG uptake in quadriceps muscle as assessed using radio-active labelled tracer (^{18}F-FDG) in PET-MRI. The secondary outcome parameter is insulin-stimulated ^{18}F-FDG uptake in BAT as assessed using radio-active labelled tracer in PET-MRI.

Nature and extent of the burden and risks associated with participation, benefit and group relatedness: This study will not induce any benefits for the subjects and the major burden will be the time investment and potential side effects of clenbuterol. In total, the subjects will visit the University of Maastricht on 6 occasions (excluding screening) for measurements. Performed measurements will be without risks, but hematomas or bruises could develop upon blood sampling or muscle biopsies taken. This risk will be minimized due to state-of-the-art techniques and sterility measures taken. Clenbuterol or placebo supplementation will be given for 28 days, in which subjects ingest 1 capsule (20 μg) twice daily (40 μg/day). Clenbuterol could induce adverse effects, e.g. headache, increased heart rate/blood pressure,

tremors, dizziness. However, during this study we will use a standard dose of clenbuterol (40 μg/day), which has previously been demonstrated to be a safe dose for human application. Furthermore, we will apply a relatively short supplementation duration (4 weeks). To limit the number of subjects that need to be included we decided for a cross-over design in which every participant serves as his/her own control. Risks related to the clamp and PET-MRI measurements are low due to clear exclusion criteria and well-experienced researchers performing these tests. For the PET scan, a [18F]-FDG bolus will be infused in the subject, which is a radio-active tracer commonly used in standard medical practice. The total radiation burden in the study per subject is ~2.7 mSv (normal background radiation in the Netherlands is ~2.5 mSv). No contrast agents are used. MRI is a modern diagnostic tool, which does not imply significant risks (no ionizing radiation).

1. INTRODUCTION AND RATIONALE

Introduction

The skeletal muscle plays a pivotal role in the regulation of the glucose homeostasis as it accounts for ~80% of post-prandial, insulin-stimulated glucose uptake in healthy individuals (1). Not surprisingly, skeletal muscle insulin resistance is a major hallmark in the development of type 2 diabetes mellitus (T2DM) (2). Importantly, we have recently uncovered a novel pathway mediating skeletal muscle glucose uptake independent of both the insulin signalling and muscle contraction pathways, but instead involved the activation of β_2-adrenergic receptors (β_2-ARs), the major subtype of β-ARs in muscle cells (3-5). In more detail, activation of β_2-ARs markedly increased GLUT4-mediated glucose uptake in both L6 muscle cells and humans primary myotubes through the activation of the mammalian target of rapamycin (mTOR) complex 2 (mTORC2) (**Figure 1**). This pathway was also shown to be physiologically relevant, meaning that the activation of β_2-ARs induced glucose uptake in skeletal muscle both *ex vivo* and *in vivo*, and these effects were abolished in β_1/β_2-ARs KO mice (3).

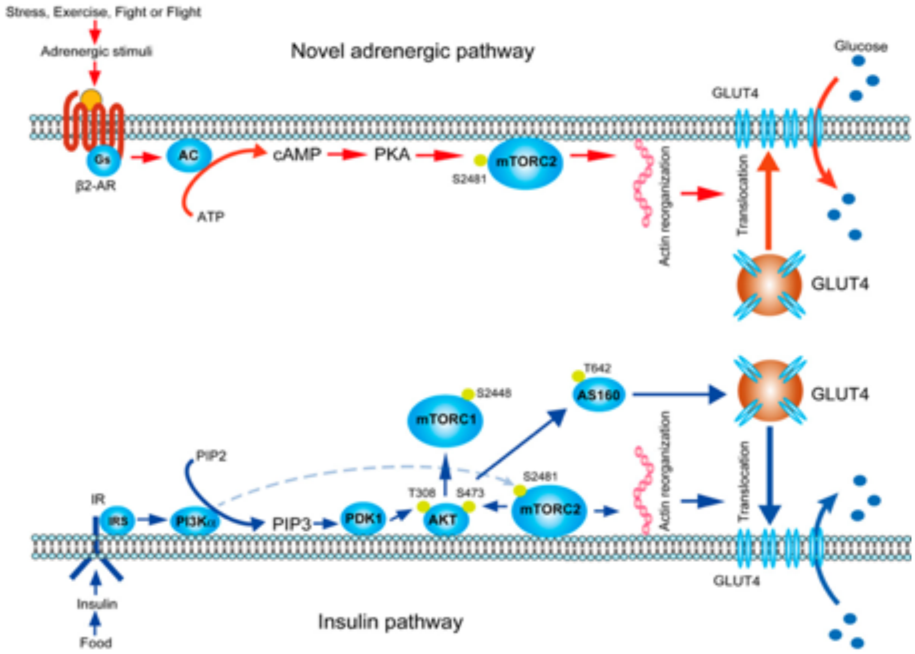

Figure 1: novel, insulin-independent pathway to stimulate glucose uptake in skeletal muscle. *The β_2-adrenoceptor pathway, when activated, couples to G_S to stimulate adenylate cyclase (AC), resulting in the production of cAMP, activation of PKA and phosphorylation of mTORC2. The phosphorylated mTORC2*

then stimulates actin reorganization and GLUT4 translocation to the plasma membrane to increase glucose uptake. The lower part of the graph represents the classical insulin signaling pathway. From ref. (3).

The existence of a pathway mediating skeletal muscle glucose disposal, independent of the activation of the insulin signalling and muscle contraction pathways, presents an interesting novel target to improve glucose disposal in the insulin resistant state. Indeed, short-term supplementation (4 days) with a relatively high dosage (30 mg/L in the drinking water) of clenbuterol, a selective β_2-adrenergic agonist, dramatically improved glucose tolerance in both Goto/Kakazaki rats and high-fat diet-induced obese (DIO) mice, two well-established animal models for diabetes (3). In addition, studies performed by Castle et al. (6) and Pan et al. (7) showed similar improvements in glucose homeostasis in obese Zucker rats upon prolonged supplementation with a relatively high-dose of clenbuterol. Interestingly, we recently found that even a low dose of clenbuterol was able to markedly improve glucose- and insulin tolerance, as well as fasting glucose, in DIO mice (8, 9). In addition, a single-dose of clenbuterol (80 µg) was recently shown to significantly increase skeletal muscle PKA concentrations in healthy male volunteers, thereby potentially hinting towards mTORC2 activation (10). Based on the latter findings, we initiated a clinical trial investigating the effects of a 2-week clenbuterol supplementation (40 µg/day) on whole-body insulin sensitivity in healthy young males (METC18-056). Despite the fact that this study is not fully finalised yet and we still await the data on glucose homeostasis, we observed that prolonged clenbuterol supplementation – at the dose also prescribed for the current study – is well-tolerated in a healthy population with relatively minor side-effects. In fact, if side-effects emerged in a subject, these effects faded over time as the drug was still consumed and completely disappeared upon withdrawal of the drug. This study hence demonstrates that clenbuterol – at the dose of 40 µg/day – can be safely administered in a human population to study the relevance of the β_2-mTORC2 pathway for alleviating insulin resistance and improving glucose homeostasis. We therefore aim to test the hypothesis that activation of the novel β_2-mTORC2 pathway via selective β_2-adrenergic stimulation increases skeletal muscle glucose uptake and beneficially affects metabolic health in overweight/obese volunteers.

Besides skeletal muscle, β-adrenergic stimulation is well-known for its activation of brown adipose tissue (BAT). BAT functions an important thermogenic organ which, upon cold exposure and/or sympathetic activation, dissipates heat through the uncoupling of mitochondrial oxidative phosphorylation via the activation of uncoupling protein 1 (UCP1) (11). Due to its capacity to increase energy expenditure and simultaneously enhance glucose clearance from the blood (11-13), BAT has been proposed as a prominent target for the treatment of T2DM. Indeed, stimulation of BAT activity through either cold exposure or selective β_3-adrenergic receptor stimulation has been reported to significantly improve glucose homeostasis in both mice and rats (14-17). In addition, transplantation of BAT into the visceral or dorsal interscapular

region in mice has been shown to protect against the detrimental effects of HFD feeding on glucose and insulin tolerance (18, 19), further highlighting the importance of the BAT in the regulation of glucose homeostasis in rodents. These studies combined strongly hint towards similar beneficial effects of BAT activation in (obese) humans, which has stimulated the search for specific pharmaceutical compounds able to stimulate BAT for over a decade. Interestingly, however, these pharmaceutical compounds (selective β_3-adrenergic agonists) have thus far not been highly successful in clinical trials unless administered at higher dosages, which is paralleled by cardiovascular side effects (20). These results thereby indicate a potential different regulatory mechanism of BAT in humans. Indeed, a recent study by Blondin et al. (21) demonstrated that human BAT – in contrast to rodents – is not regulated by the β_3-adrenergic receptor, but instead involves the activation of the β_2-adrenergic receptor. As such, selective β_2-adrenergic agonists could potentially be used to stimulate BAT activation and thereby improve glucose homeostasis in T2DM patients. However, it remains to be established whether prolonged stimulation of the β_2-adrenergic receptor can enhance BAT mass and activity, and thereby glucose homeostasis, in overweight/obese individuals. Therefore, we also aim to investigate the effect of prolonged clenbuterol supplementation on BAT mass and activity in overweight/obese volunteers.

2. OBJECTIVES

Primary Objective:

- To determine if prolonged treatment (4 weeks) with the selective β_2-adrenergic agonist clenbuterol improves *in vivo* quadriceps glucose disposal via the mTORC2 pathway in overweight/obese male and (postmenopausal) women.

Secondary Objective(s):

- To determine if prolonged treatment (4 weeks) with the selective β_2-adrenergic agonist clenbuterol enhances BAT mass and activity in obese male and (postmenopausal) women.

Explorative objectives:

- Does prolonged (4 weeks) administration with the selective β_2-adrenergic agonist clenbuterol affect:
    - Body weight/composition
    - Glucose infusion rate (GIR) during the one-step clamp
    - Skeletal muscle GLUT4 translocation
    - Plasma substrates
    - Heart rate and blood pressure
    - (Sleeping) energy expenditure and substrate oxidation
    - Skeletal muscle glycogen and lipid content
    - Gene and protein expression in skeletal muscle
    - Femoral artery flow mediated dilation

3. STUDY DESIGN

To investigate the effects of prolonged treatment with a selective β_2-agonist on *in vivo* quadriceps glucose uptake, a clinical trial will be conducted with 20 obese male and (postmenopausal) women. Thus, in a randomized, placebo-controlled, double-blinded, cross-over design, subjects will receive either the selective β_2- agonist clenbuterol (40 µg/day) or a placebo for 4 weeks with a wash-out period of at least 6-8 weeks **(Figure 2)**. The study ends when 14 participants completed the second intervention period.

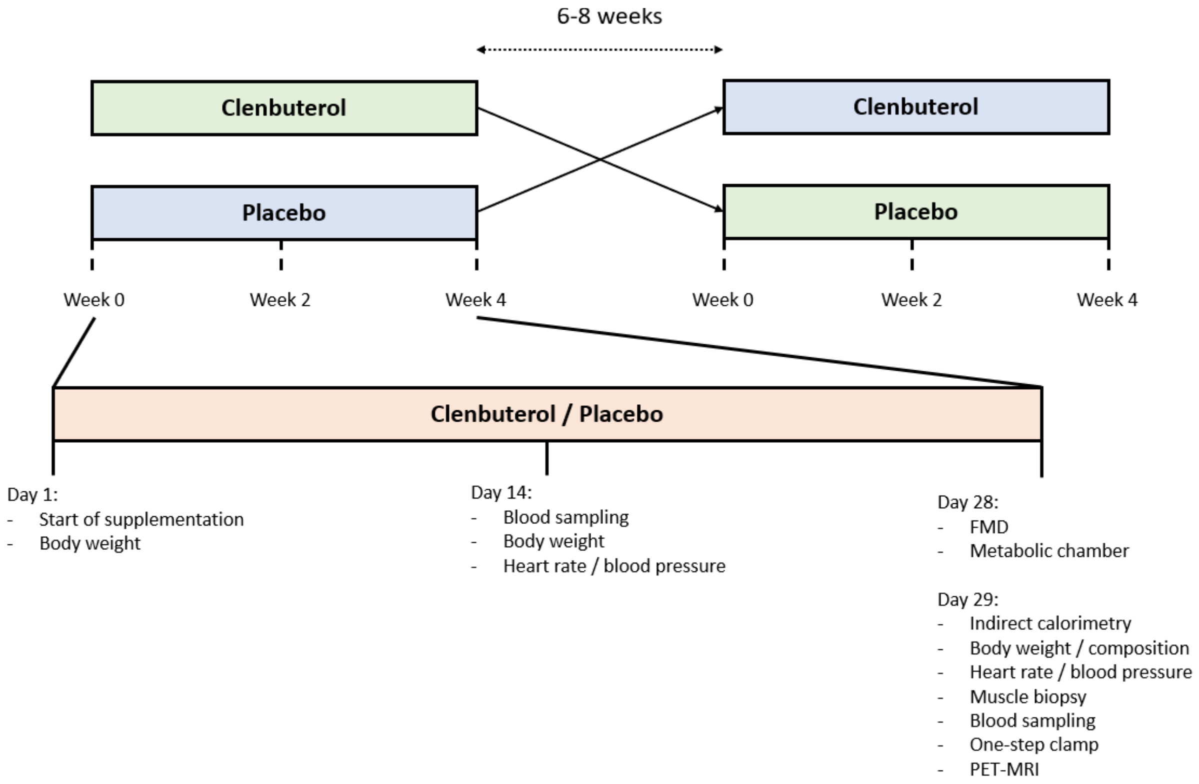

Figure 2: study design. *A randomized, placebo-controlled, double-blinded, cross-over design, in which subjects will receive either daily clenbuterol hydrochloride (40 µg/day) or placebo supplementation for 4 weeks with a wash-out period of at least 6-8 weeks. Participants will be randomly allocated to one of the study arms. Several metabolic read-out parameters will be evaluated upon treatment. Subjects will receive and start with the medication on day 1. This medication will last till visit 2. After 14 days of supplementation, a fasting blood sample will be taken, body weight will be measured, and heart rate and blood pressure will be measured. Subjects will receive new medication that lasts till the end of the supplementation period. After 28 days of supplementation, 24-hour urine will be collected, blood flow will be measured by means of FMD whereafter subjects will spend the rest of the night in a respiration chamber to measure sleeping energy expenditure. Following an overnight fast, body composition will be measured*

by means of a BodPod. A muscle biopsy will thereafter be taken followed by the performance of a one-step hyperinsulinemic-euglycemic clamp. Following the one-step clamp, PET-MRI will be performed to assess insulin-stimulated ^{18}F-FDG skeletal muscle and brown adipose tissue glucose uptake. All measurements within the first treatment period will be repeated within the second treatment period. A minimal wash-out period of 6-8 weeks will be applied. All participants will be closely monitored for AEs during the supplementation period.

For this study, the preferred drug of use is the selective β_2-adrenergic agonist clenbuterol. Clenbuterol can – in contrast to many other selective β_2- agonists – be administered orally due to its water-soluble composition and is additionally long acting (T_{50} = 35 hours, (22)), thereby ensuring systemic stimulation throughout the day. To further minimize the risk of adverse events and to prevent any confounding effects of changes in body composition that are commonly associated with prolonged treatment with high doses of clenbuterol (23), a standard dose of 40 µg/day (normal recommended dose for clinical use: 40-80 µg/day) - which in our experience (METC18-056) is well-tolerated in humans – will be applied during this study.

3.1 Detailed study design

After an initial screening, subjects will visit the Metabolic Research Centre at Maastricht University (MRUM) at 6 occasions (3 occasions per study arm) (**for details please see Section 8.3 → 'study procedures'** and Table 1) with a minimal wash-out period of 6-8 weeks:

Screening *(time investment = 1 hour)*

After the subject has shown interest in the study, a screening will be scheduled to assess whether the subject is suitable for the study. The subject will arrive at the university after an overnight fast. The screening will include filling in a medical history and physical activity questionnaire and MRI-contra-indications questionnaires (See Document F1). Body weight, heart rate and blood pressure will be measured, an ECG will be performed followed by the collection of a fasted blood sample (~15 mL) for determination of clinical health parameters, including markers for kidney and liver function (creatinine, ASAT, ALAT and gamma-GT for liver).

Study period

Day 1 *(time investment = 15 minutes)*

At the start of each treatment period, subjects will arrive at the MRUM in the morning after an overnight fast (i.e. no food consumption after 22:00 the night before). Body weight will be measured after which subjects will be provided with 2 weeks' worth of supplements (+ 3 days). Subjects will be instructed on the

dose, frequency, and route of administration of the supplements. Furthermore, subjects will receive a diary to report potential side effects. The subjects are instructed to get in contact with the researcher in case any side effects emerge.

Day 14 *(time investment = 30 minutes/study arm)*

After 2 weeks of supplementation, subjects will arrive at the MRUM in a fasted state (prior to clenbuterol/placebo ingestion for that day) for a safety check-up. During this visit, the diary for side effects will be discussed. Body weight, heart rate and blood pressure will be measured, followed by a fasting blood sample collection for determination of circulation plasma substrate concentrations (~15 mL). Subsequently, participants will receive an empty urine container with HCL for 24-hour urine collection on day 28 to assess protein oxidation. They will be instructed to collect urine in this container from the morning of day 28, excluding the first urine of the day, up until the point they arrive at university and enter the respiration chamber (~18:00). Furthermore, the dietary consumption of subjects will be monitor via a food diary. This diary must be filled out by the subject on day 28. Afterwards, subjects will receive 2 weeks' (+ 3 days) worth of supplements and are allowed to go home.

Day 28 *(time investment day 28 and 29 = 20 hours/study arm)*

On day 28 of each study arm, participants collect their urine (at home) in the provided container from the morning of day 28, excluding the first urine of the day, up until the point they arrive at university and enter the respiration chamber (~18:00). Subjects will arrive at the MRUM at 17:00 and a femoral artery flow mediated dilation (FMD) measurement will be performed. Afterwards, subjects will receive a standardized meal provided by the researcher and the last clenbuterol/placebo capsule is ingested. Around 18:00, subjects will enter a respiration chamber where they will stay overnight. This whole-room calorimeter is equipped with a toilet, sink, bed, desk, computer and television. At 11:00 pm, the lights will be turned off, and the subject is instructed to try to sleep. The overnight stay in the respiration chamber is performed to measure the subject's sleeping metabolic rate upon clenbuterol/placebo supplementation, as well as to create equal conditions between both study periods prior to the primary and secondary outcomes, i.e. the insulin-stimulated skeletal muscle and BAT [18F]-FDG uptake as assessed by means of PET-MRI. During their stay in the respiration chamber, we will also collect urine. In this way, we can collect urine over a 24-hour (2x12 hour of which 12 hours at home and twelve hours at the university) period to assess protein oxidation.

Day 29 *(time investment day 28 and 29 = 20 hours/study arm)*

On day 29 of the supplementation period of each study arm, subjects will be woken up in the respiration chamber at 06:00 and body composition will be assessed by means of the Bodpod (Cosmed). Afterwards, baseline energy expenditure and substrate oxidation will be measured for 30 minutes. Blood pressure will

be measured, and a fasted blood sample will be collected for determination of circulating plasma substrate concentrations (~15 mL). A skeletal muscle biopsy will be taken at baseline for the determination of the effect of prolonged clenbuterol supplementation on GLUT4 translocation, lipid content and gene and protein expression (i.e. mTORC2, insulin signalling, and AMPK pathways). Following these biopsies, a one-step hyperinsulinemic-euglycemic clamp will be performed to assess insulin-stimulated peripheral glucose uptake. The clamp procedure is explained in section **8.3.2. Measurement**. Following the 'steady state' of the clamp, the subject (still receiving insulin and glucose infusion) will be positioned in the PET-MRI scanner (located in the MUMC+) and will receive a bolus of circa ~1.0 MBq/kg (~75 MBq for a 75 kg individual = 1.35 mSv) [^{18}F]-FDG via the cannula in the antecubital vein (wherein also glucose and insulin are infused). We will then determine [^{18}F]-FDG uptake into the quadriceps muscles by means of dynamic scanning for 60 minutes. When the dynamic scan has been completed, the subject will be repositioned in the scanner for a static scan of BAT [^{18}F]-FDG uptake. Afterwards, the insulin infusion will be stopped, and the subject will receive lunch.

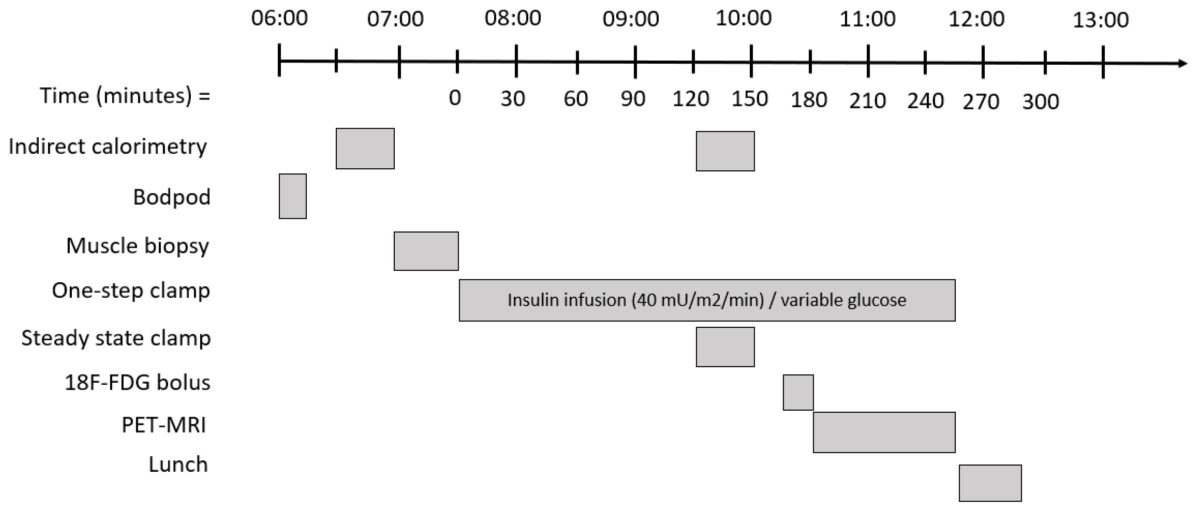

Figure 3. Time-line and measurements of day 28 of each supplementation period

Table 1. Overview of measurements and time investment

Day	Effect of clenbuterol	Test	Time per visit (hours)	Total time (hours)
0		Screening: body weight, heart rate, blood pressure, blood sampling, ECG	1	1
1	-	Body weight, subjects receive supplements and diary for side effects	0.25	0.5
14	Long-term	Body weight, heart rate, blood pressure and blood sampling, subjects receive food diary for day 28	0.5	1
28	Long-term	FMD, Metabolic chamber, urine collection	20	40
29	Long-term	Bodpod, muscle biopsy, one-step hyperinsulinemic-euglycemic clamp, PET-MRI of quadriceps and BAT, ^{1}H-MRS of hepatic lipid content.		
**Total amount of time investment**				42.5

4. STUDY POPULATION

4.1 Population (base)

The study population will consist of healthy overweight/obese male and postmenopausal women (40-75 years, BMI 25-35 kg/m^2). For recruitment procedures see section 8.3.1. Subjects must be able to provide written informed consent, meet all the inclusion criteria and none of the exclusion criteria.

4.2 Inclusion criteria

In order to be eligible to participate in this study, a subject must meet all of the following criteria:

1. Male or (postmenopausal; defined as 1 year after the last cycle) female;
2. Age between 40-75 years;
3. BMI: 25-35 kg/m^2;

4.3 Exclusion criteria

A potential subject who meets any of the following criteria will be excluded from participation in this study:

1. Not meeting all inclusion criteria
2. Cardiovascular disease (determined by means of questionnaires, heart rate/blood pressure measurements and an ECG)
3. Respiratory diseases (including asthma, bronchitis and COPD);
4. Unstable body weight (weight gain or loss > 3 kg in the last three months);
5. Intention to lose or gain body weight (e.g. with caloric restriction or physical activity)
6. Excessive alcohol and/or drug abuse;
7. Hypokalaemia;
8. Hyperthyroidism
9. Anaemia;
10. Epilepsy;
11. Smoking;
12. Renal and/or liver insufficiency;

13. Diagnosed with type 1 or type 2 diabetes mellitus;
14. Any contra-indications to MRI scanning. These contra-indications include patients with:
    a. Electronic implants such as pacemakers, defibrillators or neurostimulators
    b. Central nervous system aneurysm clip
    c. Some hearing aids (such as cochlear implant) and artificial (heart) valves which are contraindicated for MRI/MRS
    d. Iron containing corpora aliena in the eye or brains
    e. Claustrophobia
15. Participation in another biomedical study within 1 month before the first study visit, possibly interfering with the study results;
16. Medication use known to hamper subject's safety during the study procedures;
17. Subjects who do not want to be informed about unexpected medical findings;
18. Subjects who do not want that their treating physician to be informed;
19. Inability to participate and/or complete the required measurements;
20. Participation in organised or structured physical exercise (>2h per week);
21. Any condition, disease or abnormal laboratory test result that, in the opinion of the Investigator, would interfere with the study outcome, affect trial participation or put the subject at undue risk;

A medical doctor will judge participation eligibility based on the medical history questionnaire, medication use and fasting blood parameters. If the medical doctor advises that a subject cannot participate, he/she will be excluded from enrolment.

4.4 Sample size calculation

The primary endpoint of this study is insulin-stimulated ^{18}F-FDG uptake in quadricep muscles as assessed by PET-MRI scanning. As no previous studies have been performed regarding the effect of prolonged clenbuterol supplementation on *in vivo* glucose uptake by quadriceps muscle in obese subjects, estimated effect size is based on an intervention study performed by Johanssen et al. (24). In this study, ^{18}F-FDG uptake was assessed in thigh muscle of 5 healthy overweight individuals during a constant insulin infusion (56 mU/m2/min). During this study, average ^{18}F-FDG uptake in thigh muscle under insulin-stimulated conditions was ~0.0142 mL/cm3/min with a standard deviation of ~0.0037 mL/cm3/min. Sample size calculation was performed using a paired sample T-test (two-sided):

$$n = \frac{\sigma^2(z_{\alpha/2} + z_{\pi})^2}{\Delta\mu^2}$$

Based on this paired samples T-test, the expected standard deviation in ^{18}F-FDG uptake in healthy obese subjects (0.0142 mL/cm3/min) and an expected mean difference of 20%, which is in our opinion a physiological relevant percentage, we calculated that 14 subjects are required to reject the null hypothesis with a probability (power) of 80%. The type 1 error probability (α) is 0.05. Since we anticipate a drop-out rate of about 30%, we assume that a total number of 20 subjects have to be included in the study. Furthermore, it is estimated that 50% of screened subjects will eventually be included in the study and, therefore, a total of 40 subjects will be screened.

5. TREATMENT OF SUBJECTS

5.1 Investigational product/treatment

All subjects will participate in both study arms in a randomized order. On day 1 and 14 of each supplementation period, subjects will receive a container with the exact number of capsules for 2 weeks (34 capsules per 2 weeks). The daily ingested dose of clenbuterol is 40 µg (2x 20 µg/capsule), which is within the advised daily dose of clenbuterol (40-80 µg/day) for clinical use. The wash-out period in between the study arms will be at least 6-8 weeks.

5.2 Use of co-intervention (if applicable)

Subjects will be advised to maintain their normal patterns during both supplementation periods with respect to overall daily activities, eating and sleeping.

Regular medication used by the subject will be communicated to the dependent medical doctor and he will advise the researcher whether the subject is allowed to participate in the study or whether the medication interferes with the study outcome parameters (see SPC text of clenbuterol, Document D2). All medication used by the participants will be reported in the electronic case report form (eCRF).

Subjects will be asked to refrain from any extensive physical activities other than daily routines. Subjects will also be asked to perform no heavy 'household' tasks three days prior to every visit (e.g. cleaning the windows, gardening or lifting heavy groceries).

5.3 Escape medication (if applicable)

Not Applicable.

6. INVESTIGATIONAL PRODUCT

6.1 Name and description of investigational product(s)

Spiropent - Clenbuterol hydrochloride

Spiropent (Clenbuterol hydrochloride, $C_{12}H_{18}Cl_2N_2O$, **Figure 4**) (See SPC of Spiropent Document D2) is a long-acting selective β_2-adrenergic agonist with sympathomimetic activity which is used for the treatment of obstructive airways diseases with reversible airway narrowing such as bronchial asthma or chronic obstructive bronchitis (25-29). Clenbuterol hydrochloride binds and activates β_2-adrenergic receptors, thereby causing stimulation of adenylyl cyclase, leading to the subsequent synthesis of cyclic-3',5'-adenosine monophosphate (cAMP). Increased levels of cellular cAMP causes smooth muscle cell relaxation (30).

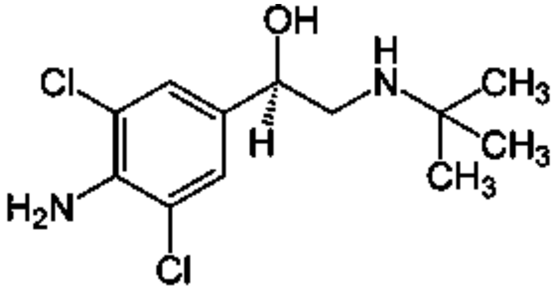

Figure 4. *Chemical structure of clenbuterol*

The normal recommended dose for clinical use of clenbuterol hydrochloride is between 40-80 µg/day (See document D2 for SPC of clenbuterol). After ingestion, clenbuterol hydrochloride is rapidly and fully absorbed in the gastrointestinal region. Maximal plasma concentrations in humans are achieved within 2-3 hours after ingestion, with maximal plasma concentrations of 0.2 ng/mL upon acute 40 µg intake (22). Binding to plasma proteins is 45-68% and clenbuterol is distributed throughout the tissues. Upon prolonged clenbuterol supplementation, plasma clenbuterol levels reach a plateau after 4 days of administration (plateau: 0.5-0.6 ng/mL with 40 µg/day intake) (22). Clenbuterol is metabolised – to a lesser extent – by the liver. In total, 8 metabolites have been found which do not possess any pharmacological activity. The excretion of clenbuterol occurs in two different phases. The T_{50} of the first phase is 1 hour, whereas the T_{50} of the second phase is 34 hours. The main route of excretion is through renal excretion in an unaltered form

(87% within 168 hours post dose) (See SPC clenbuterol, Document D2). Within 168 hours, approximately 97% of the administered dose is excreted.

Each clenbuterol hydrochloride tablet contains 109.98 mg of lactose monohydrate and 0.02 mg of clenbuterol hydrochloride (See SPC of clenbuterol D2). Clenbuterol hydrochloride tablets will be encapsulated by the GMP certified pharmacy of the Radboud UMC (See document D4) to a total dose 20 μg/capsule according to GMP standards. The capsules will be further filled with an inactive compound. Capsules of 20 μg will be ingested twice daily combined with breakfast and dinner.

Placebo

Identical placebo capsules will be created by the pharmacy of Radboud UMC, which contains the same inactive ingredients as the clenbuterol capsules but without the active ingredients.

6.2 Summary of findings from non-clinical studies

See Document D2 for a summary of findings from non-clinical studies.

6.3 Summary of findings from clinical studies

See Document D2 for a summary of findings from clinical studies.

6.4 Summary of known and potential risks and benefits

The side effects of clenbuterol are generally known for the pharmacological group of beta-sympathomimetics. These side effects include (see Document D2):

- Cardiac disorders:
    - palpitations, tachycardia, cardiac arrest, in very rare cases atrial fibrillation, in isolated cases, myocardial infarction; of unknown frequency: myocardial ischemia.
- Nervous system disorders:
    - trembling, anxiety, headache, dizziness, insomnia, redness, sweating, excitement.
- Respiratory, breast and mediastinum:

    - paradoxical bronchospasm disorders.
- Disorders of the kidneys and urinary tract:
    - rarely mycosis disorders.
- Disorders of the musculoskeletal system and connective tissue:
    - muscle cramps.
- Metabolism and nutrition disorders:
    - hypokalaemia, hyperglycaemia.
- Disorders of the blood vessels:
    - a decrease or a sharp rise in blood pressure.
- Immune system disorders:
    - skin rash, angioedema.

6.5 Description and justification of route of administration and dosage

The daily recommended dose of clenbuterol hydrochloride is between 40-80 µg/day in adults, which has to be administered orally together with a meal. In this study, we will administer 40 µg of clenbuterol a day (2x 20µg), which is within the daily recommended dose. Capsules have to be administered orally in the morning together with breakfast and in the evening together with dinner.

6.6 Dosages, dosage modifications and method of administration

Clenbuterol tablets will be encapsulated by the pharmacy of Radboud UMC in accordance with GMP and local regulatory guidelines. These capsules are then filled further with an inactive compound. These capsules have a final dose of 20 µg/capsule and can be directly swallowed with a drink (water, fruit juices etc.).

6.7 Preparation and labelling of Investigational Medicinal Product

The study drug, as well as the placebo, will be packaged into bottles containing the correct number of capsules for 2 weeks' (+ 3 days) supplementation by the pharmacy of Radboud UMC. The labels will be prepared in accordance with both GMP and local regulatory guidelines, which will thereby fulfil all requirements for labelling (See document D3), including:

- The name, address and telephone number of the researcher (Pip van Lier).
- Pharmaceutical formulation, route of administration, doses
- Batch number
- Information to identify the clinical trial (ABR number and EudraCT).
- Subject number
- Instructions on the use of the product
- Storage conditions
- Expiration date
- Notification to keep out of reach of children

Label texts will be provided in Dutch. Since the study will be performed in a double-blinded manner, both clenbuterol containing capsules, as well as the placebo capsules, will be delivered in identical bottles. These bottles will be stored under appropriate conditions at the research facility and kept at room temperature.

6.8 Drug accountability

All study medication will be encapsulated, packed, blinded and labelled by the pharmacy of Radboud UMC. The study medication will thereafter be sent to the hospital pharmacy (AMC) where it will be stored under the appropriate conditions. The researcher (Pip van Lier) will be able to collect the bottles for a subject with a recipe provided by the dependent physician. The bottles will thereafter be stored in a temperature-controlled room before they are given to a subject. The number of the bottles will be noted by the researcher in a log before the bottle is given to the subject. The subject is asked to return used bottles at the end of the period and the number of capsules left in the bottles will be counted. Subjects should always return all unused study drugs/empty bottles to the researcher. Left-over medication of subjects who have finished the study will be destroyed by the AMC. All dispensations of bottles will be carefully documented by the researcher in a log and eCRF.

7. NON-INVESTIGATIONAL PRODUCT

7.1 Name and description of non-investigational product(s)

- **Lidocainehydrochloride**:
  - Local anaesthetic (10 mg/mL) used by means of injection. Used during the muscle biopsy.
- **Glucose 20% (Baxter)**:
  - 20% w/v, solution for injection. Infused during the hyperinsulinemic-euglycemic clamp
- **Insulin aspart (100 IE/mL) (Novorapid):**
  - Peptide hormone, solution for injection. Infused during the hyperinsulinemic-euglycemic clamp

7.2 Summary of findings from non-clinical studies

Not applicable, all products have been approved for clinical research.

7.3 Summary of findings from clinical studies

Not applicable, all non-investigational products will be used as in standard medical practice:

- SPC lidocaine – see attachment document D2
- SPC glucose 20% - see attachment document D2
- SPC insulin Aspart – see attachment document D2

7.4 Summary of known and potential risks and benefits

Not applicable, all non-investigational products will be used as in standard medical practice:

- SPC lidocaine – see attachment document D2
- SPC glucose 20% - see attachment document D2
- SPC insulin Aspart – see attachment document D2

7.5 Description and justification of route of administration and dosage

Not applicable, all non-investigational products will be used as in standard medical practice.

7.6 Dosages, dosage modifications and method of administration

For the muscle biopsy, lidocaine (10 mg/mL) will be injected subcutaneously, as well as under the muscle fascia at the site of the muscle biopsy of the *m.* vastus lateralis.

During the one-step hyperinsulinemic-euglycemic clamp, an insulin infusion (1 IE/mL) will be prepared for intravenous administration according to the following instructions: 0.5 mL of 100 IE/mL Novarapid insulin diluted in 47.5 mL NaCl (9%) and 2 mL blood collected from the participant. The preparation of the insulin infusion will be checked and co-signed by the researcher and a second experienced researcher.

A total of 5.0 mL KCl 7.5% is added to 500 mL of glucose 20% infusion bag, to prepare glucose for intravenous infusion. It will be administered at variable infusion rates, in order to keep glucose values around 6.0 mmol/L.

7.7 Preparation and labelling of Non Investigational Medicinal Product

All non-investigational products will be stored appropriately. Lidocaine will be transferred to a syringe prior to administration.

7.8 Drug accountability

All non-investigational products have been regularly used for many studies by our research group. All non-investigational products are purchased from the MUMC pharmacy. All non-investigation products arrive in their original packaging and will be stored as recommended.

8. METHODS

8.1 Study parameters/endpoints

8.1.1 Main study parameter/endpoint

Primary outcome parameter:

- Insulin-stimulated ^{18}F-FDG uptake into quadriceps muscle (expressed as Ki) as assessed by means of PET-MRI.

8.1.2 Secondary study parameters/endpoints (if applicable)

Secondary study parameters:

- Insulin-stimulated ^{18}F-FDG uptake in BAT (expressed as Ki) as assessed by means of PET-MRI

8.1.3 Other exploratory study parameters (if applicable)

Other study parameters:

- Body weight/composition
- Glucose infusion rate (GIR) during the one-step clamp
- Skeletal muscle GLUT4 translocation
- Plasma substrates
- Heart rate and blood pressure
- (Sleeping) energy expenditure and substrate oxidation
- Skeletal muscle glycogen and lipid content
- Gene and protein expression in skeletal muscle
- Femoral artery flow mediated dilation

8.2 Randomisation, blinding and treatment allocation

A randomisation list for the study will be created by an independent researcher of the Department of Nutrition and Movement Sciences (Dr E. Phielix). To ensure that the order in which the medication is given (Treatment A: i.e. clenbuterol – placebo or Treatment B: placebo – clenbuterol) occurs equally frequent in the entire subject group. This randomisation list will be created with the help of the website

www.randomizer.org and will be performed in groups of 4 participants for the entire study. This randomization list containing the participant codes and allocation to the treatment A or B will be send to the pharmacy of Radboud UMC. Accordingly, the pharmacy of Radboud UMC prepares the study medication bottles for the participants. Four envelopes containing the unblinding key for the intervention arms A and B study will be prepared by the pharmacy of Radboud UMC. These envelopes will be send to the project leader (Dr. J. Hoeks), the independent physician (Dr. Ronald Henry), the secretary of the Department of Nutrition and Movement Sciences (Yolanda Verhaegen) and the responsible medical physician of the study (Dr. T. Weijer van de). All these players are responsible to keep these envelopes safe. The envelopes will be opened only in situations where de-blinding of the study is necessary. In view of the nature of this study no indications for breaking the randomization code are predetermined, neither expected. In a remote possibility of any untoward effect, the persons mentioned above can break the blinding code for the particular participant.

Participants that come to the MRUM for an initial screening will be given a screening code (SCR + number) and, in case the subject is allowed to participate in the study, the subject will be given a subject code (CLB + number). This subject code is linked to the randomisation list (i.e. CLB01 will receive treatment allocation of randomisation number 1). In this way, both subject and researcher will be blinded for the treatment.

8.3 Study procedures

8.3.1. Recruitment

Subjects will be recruited in Maastricht and surroundings by means of posters and advertisements in local newspapers and online (See document E3). Also, subjects that previously participated in other studies at the Department of Nutrition and Movement Sciences at Maastricht University or were screened and found not eligible to participate in other studies will be approached by means of a standardized email providing a short explanation of the study design (See Document K6). This type of recruitment will only occur if subjects indicated that they want to be approached for participation in future studies (signed consent) and appear to fulfill the inclusion criteria. The researchers will contact individuals who are interested in participating in the study by telephone, only after the individual has sought contact with the researcher on his or her own accord first. By contacting responders by telephone first, the burden of travel and time effort is reduced for the potential subject and researchers. In the telephone interview, the goal of the study will be explained, and the basic inclusion criteria will be discussed. When responders are interested, they will receive detailed subject information via e-mail or regular mail. They will be instructed to read this

information carefully and to ask questions if things are unclear. The researcher will contact the possible participant again at least 7 days after the study information was received by the participant. If responders want to participate after reading the study information and seem to be eligible, they are invited for a screening.

8.3.2. Screening

Only when the informed consent is signed, participants can undergo the screening. Both the participant as well as the researcher will sign the informed consent before the start of the screening. The screening will include the following procedures:

- together with the participant, a questionnaire will be filled on health status, physical activity and medical history (see document F1)
- MRI contra-indication questionnaire (see document F1)
- a fasting blood sample will be drawn to determine clinical health parameters including parameters for liver and kidney function
- physical examination, like body weight and length
- An ECG will be taken
- blood pressure will be measured 3 times in row at the contra-dominant arm

A total of 15 ml blood will be drawn.

Body weight

During the screening, body weight will be measured by means of a digital balance with an accuracy of 0.001 kg (seca, seca GmbH & Co, Hamburg, Germany).

Questionnaires

Questionnaires regarding medical history and physical activity will be filled in by the subjects during the screening (See document F1). In addition, a MRI contra-indication questionnaire will be filled in by the subject (See document F1).

Blood samples and invasive biomarkers

Fasted blood samples will be taken during the screening by means of venepuncture. Blood samples will be used for the determination of clinical health parameters. The analyses will be performed by the Maastricht University Medical Centre.

8.3.3. Measurements during study period

Body weight

During the supplementation periods (T = 1, 14 and 29 days of each study arm), body weight will be measured by means of a digital balance with an accuracy of 0.001 kg (seca, seca GmbH & Co, Hamburg, Germany).

Heart rate and blood pressure

Resting heart rate and blood pressure will be measured 3 times in a row in the contra-dominant arm at three occasions of each study arm (T = 14 and 29 days of each study arm) by means of an automatic inflatable cuff (Omron Healthcare, Hamburg, Germany).

Indirect calorimetry

Energy expenditure and substrate oxidation will be measured by means of an open circuit respirometry with an automated ventilated hood system (Omnical, Maastricht Instruments, Maastricht University, Maastricht, The Netherlands). This will be performed both acutely (after 2 days of supplementation) and after 4 weeks of supplementation.

Muscle biopsies

Muscle biopsies will be taken from all subjects after long-term (4-weeks) clenbuterol/placebo supplementation. Muscle biopsies from the m. vastus lateralis will be taken and performed under local anaesthesia with Lidocaine 1.0% solution (10mg/mL) without adrenalin. A side-cutting needle will be used to acquire the muscle tissue, following the Bergström method (31). The leg of the biopsy will be randomized to exclude influence of a relatively more trained muscle versus a relatively less trained muscle. The biopsy material will be processed *ex vivo* immediately and will be frozen in isopentane cooled with liquid nitrogen and stored at minus 80 °C for later analyses. For these analyses, about 300mg of muscle tissue is needed, being a standard amount of tissue that is to be acquired in each biopsy.

Bodpod

The Bod Pod® (Cosmed) will be used to determine body composition and measures fat mass, fat free mass, total body mass and estimates resting metabolic rate (RMR) via whole body densitometry. The Bod Pod utilizes the displacement of air during the measurement to determine body composition. The volume of air

a person's body displaces is measured while sitting inside a comfortable chamber for two 50- second measurements. It is a safe, non-invasive, easy-to-use and quick tool for measuring body composition. The duration of the measurement is approximately 5 minutes.

Metabolic chamber: sleeping energy expenditure and substrate oxidation

Subjects will spend the night from day 28 to 29 of each study arm in a respiration chamber, which is equipped with indirect calorimetry (Omnical, Maastricht Instruments, Maastricht University, Maastricht, The Netherlands) and measures oxygen consumption and carbon dioxide production. Based on these values, the sleeping metabolic rate and substrate oxidation will be calculated. Subjects will receive a standardized meal before they enter the chamber and will enter at around 18:00 hours. The respiration chamber is a $14m^2$ room furnished with a bed, chair, desk, TV, telephone, computer, washbowl and toilet. The room is ventilated with fresh air. Privacy is warranted, as the subject in the chamber can close the curtains of the outside windows.

Femoral artery flow mediated dilation (FMD)

Femoral artery flow mediated dilation (FMD) is assessed by Echo-Doppler (MyLab^{TM}Gamma, Esaote) by using a 7.5-MHz transducer and recording of echo images on the laptop. After a 3-minute reference period, the pneumatic cuff placed around the participant's leg is inflated 50 mmHg above systolic pressure for 5 minutes, causing distal hypoxia. Upon cuff-release reactive hyperemia ensues. The echo images are processed automatically to determine the diameter profiles over the entire 15 min of the femoral artery FMD measurement using a custom-written Matlab program (MyFMD, Prof. A.P.G. Hoeks, Department of Biomedical Engineering, Maastricht University, Maastricht, the Netherlands). The FMD response is then quantified as the maximal percentage change in post occlusion arterial diameter relative to baseline diameter.

One-step hyperinsulinemic-euglycemic clamp

A one-step hyperinsulinemic-euglycemic clamp will be performed on day 29 for the measurement of whole-body insulin sensitivity upon prolonged clenbuterol/placebo supplementation. The clamp will start around 07:30 in the morning after an 12-hour overnight fast. A teflon cannula will be inserted into an antecubital veins of one arm for the infusion of insulin and glucose. Another cannula will be inserted retrogradely into a superficial dorsal hand vein. This venous blood will be arterialized by placing the hand into a hotbox, which heats the air inside (50 ˚C). Baseline blood samples (T = 0) will be taken (~20 mL) for the assessment of baseline plasma substrate concentrations. Then, the 3h high-insulin clamp will be started (T = 0-180 min) with a high primed constant infusion of insulin (40 mU/m2/min). Plasma glucose levels will be clamped at

~6.0 mmol/L by variable co-infusion of 20% glucose. At regular time points (every 5 to 10 minutes), a small volume of blood (0.1 mL) will be sampled for immediate determination of plasma glucose concentration. When necessary, glucose infusion rate will be adjusted to maintain plasma glucose levels around ~6.0 mmol/L (euglycemia). A steady state will be reached at t=150 min and glucose infusion rates (GIR) will be kept stable between t=150 and t=180 min. This period will therefore be called 'the steady state'. The GIR during this steady state phase is a measure of whole-body insulin sensitivity. From t=150 till t=180 min, also indirect calorimetry will be performed to measure energy expenditure and substrate oxidation.

DNA analyse

A fasting blood sample will be collected during the baseline phase of the one-step clamp. Out of the fasted blood sample, DNA will be extracted from leucocytes in this sample to study genetic variants that may affect hormones and energy metabolism. The results will not have any consequence for the future health condition of the participants, does not have any consequences for the family and is not used for diagnosis or medical treatment. Therefore, the patient will not be informed about the DNA results.

PET-MRI scan

A dynamic and static ^{18}F-FDG PET-MRI scan will be performed under insulin-stimulated conditions (40 mU/m2/min) after the 'steady state' of the one-step clamp. The subject will be positioned in the PET-MRI scanner, whilst still receiving a continuous insulin and glucose infusion. The subject is then injected with ^{18}F-FDG tracer and a dynamic PET-MRI scan will be performed for 60 minutes for the measurement of insulin stimulated ^{18}F-FDG uptake in quadriceps muscles (one bed position, multiple scan frames). We intend to do this dynamically to not only quantify the (absolute) amount of uptake, but also the rate/speed of glucose uptake, as both these factors are associated with insulin sensitivity. Following this dynamic scan, the subject will be positioned in the PET-MRI with the torso to perform a static scan for the measurement of the amount of glucose uptake by BAT for 15 minutes.
The radiation dose or the injected radioactive tracer ^{18}F-FDG is 0.018 mSv per MBq. For adequate imaging a radiation dose with injection of ~1.0 MBq/kg is required (=75 MBq for a 75 kg individual), resulting in a radiation burden of ~1.35 mSv for the subject per scan. Given that every subject is scanned in two different conditions (clenbuterol hydrochloride and placebo intervention), the total radiation burden for this study is 2 x 1.35 = ~2.7 mSv. This is considered a low risk (as stated by the Annals of ICRP (32)), considering that the background radiation received in the Netherlands is 2.5 mSv. The effective dose is between 1-10 mSv (risk category $<10^{-4}$), which is recommended and acceptable for research conducted with healthy adults and

has been approved of by the Medical Ethics Committee in earlier studies performed by our group (i.e. see also METC10-3-027).

MRS and MRI are modern diagnostic tools that do not imply significant risks (no ionizing radiation). During the scans the subjects will receive a buzzer to let the researcher know when they are uncomfortable and they will stay in contact with the researcher via an intercom communication system. The acquisition of MRI images is accompanied by a 'clanging' noise. Therefore, subjects will be provided with protective headphones. There is a chance that MRI reveals an unexpected medical condition, of which the subject will be informed. His physician
will also be informed. All MRI images will be screened for unexpected medical findings by a radiologist or a radiologist in training.

24-hour urine collection

24-hour urine will be collected on day 28 to assess protein oxidation. For this purpose, subjects will receive an empty urine container with HCL during the visit on day 14. They will be instructed to collect urine in this container from the morning of day 28, excluding the first urine of the day, up until the point they arrive at university and enter the respiration chamber (~18:00). During their stay in the respiration chamber, we will also collect urine. In this way, we can collect urine over a 24-hour (2x12 hour) period to assess the individual protein oxidation of the participant.

8.3.4. Analyses

Blood samples

Fasting blood samples acquired during the screening will be analyzed by the Maastricht University Medical Centre for determination of clinical health parameters.

Collection of fasting blood samples will be performed in the appropriate tubes on day 14 and 28 of the supplementation periods. Blood samples will be centrifuged at high-speed and blood plasma will be transferred to a new tube. Plasma will be immediately flash-frozen in liquid nitrogen and stored in -80 degrees Celsius until further analyses.

Glycogen content

Muscle glycogen content will be analysed in the frozen muscle biopsies by means of a commercial glycogen kit (Abcam).

Gene and protein expression

Skeletal muscle will be assessed for gene and protein expression of several metabolic pathways (e.g. insulin signalling pathway, AMPK-pathway, mTORC2 pathway and mitochondrial markers) by means of RT-qPCR or Western blot, respectively. For the assessment of the activation of the mTORC2 pathway, we will analyse the phosphorylation of mTOR at SER2481 (p-mTOR S2481) by means of a western blot.

Immunohistochemistry

Determination of skeletal muscle GLUT4-translocation and lipid content will be performed by means of immunohistochemistry and assessed by wide-field microscopy.

8.4 Withdrawal of individual subjects

Subjects can leave the study at any time for any reason if they wish to do so without any consequences. The investigator can decide to withdraw a subject from the study for urgent medical reasons.

8.4.1 Specific criteria for withdrawal (if applicable)

The investigator can decide to withdraw a participant from the study for the following reasons:

- Non-medical reasons e.g. request by the participant or non-compliance to the study
- Medical reasons argued to be significant by the medical responsible doctor/researcher and/or participant.
- Protocol violation
- In case of illness or changes use of medication of the participant

In all cases, the researcher together with the medical responsible doctor decides whether the participant may continue the study or not.

8.5 Replacement of individual subjects after withdrawal

The subject will not be replaced upon withdrawal from the study since subject drop-out has been anticipated for in the sample size calculation.

8.6 Follow-up of subjects withdrawn from treatment

No follow-up of the subject will be performed upon withdrawal. In case a subject drops out of the study due to medical complication, the research team will provide the subject with guidance to the responsible medical doctor of the study and eventually be referred to the general practitioner.

8.7 Premature termination of the study

In case of premature termination of the study, the METC AzM/UM and CCMO (competent authority) will be informed within 15 days after termination. Both the METC and CCMO will be notified regarding the reason of premature termination of the study using the appropriate documents. Potential reasons for premature termination of the study include:

- the judgment of the competent medical research ethics committee that has assessed the study is irrevocably revoked;
- a reasonable case can be made for terminating the study in the interests of the health of the research subjects;
- it transpires that continuation of the study cannot serve any scientific purpose, and this is confirmed by the medical research ethics committee that has issued a positive decision on the study;
- the principal investigator is no longer capable of performing the tasks of the principal investigator, and no replacement can be found;
- circumstances beyond the control of the investigator make it impossible or unnecessary to continue the study

9. SAFETY REPORTING

9.1 Temporary halt for reasons of subject safety

In accordance to section 10, subsection 4, of the WMO, the sponsor will suspend the study if there is sufficient ground that continuation of the study will jeopardise subject health or safety. The sponsor will notify the accredited METC without undue delay of a temporary halt including the reason for such an action. The study will be suspended pending a further positive decision by the accredited METC. The investigator will take care that all subjects are kept informed.

9.2 AEs, SAEs and SUSARs

9.2.1. Adverse events (AEs)

Adverse events are defined as any undesirable experience occurring to a subject during the study, whether or not considered related to the investigational product. All adverse events reported spontaneously by the subject or observed by the investigator or his staff will be recorded.

9.2.2. Serious adverse events (SAEs)

A serious adverse event is any untoward medical occurrence or effect that

- results in death;
- is life threatening (at the time of the event);
- requires hospitalisation or prolongation of existing inpatients' hospitalisation;
- results in persistent or significant disability or incapacity;
- is a congenital anomaly or birth defect; or
- any other important medical event that did not result in any of the outcomes listed above due to medical or surgical intervention but could have been based upon appropriate judgement by the investigator.

An elective hospital admission will not be considered as a serious adverse event.

The investigator will report all SAEs to the sponsor without undue delay after obtaining knowledge of the events. The sponsor will report the SAEs through the web portal *ToetsingOnline* to the accredited METC that approved the protocol, within 7 days of first knowledge for SAEs that result in death or are life threatening followed by a period of maximum of 8 days to complete the initial

preliminary report. All other SAEs will be reported within a period of maximum 15 days after the sponsor has first knowledge of the serious adverse events.

9.2.3. Suspected unexpected serious adverse reactions (SUSARs)

Adverse reactions are all untoward and unintended responses to an investigational product related to any dose administered.

Unexpected adverse reactions are SUSARs if the following three conditions are met:

1. the event must be serious (see chapter 9.2.2);
2. there must be a certain degree of probability that the event is a harmful and an undesirable reaction to the medicinal product under investigation, regardless of the administered dose;
3. the adverse reaction must be unexpected, that is to say, the nature and severity of the adverse reaction are not in agreement with the product information as recorded in:
   - Summary of Product Characteristics (SPC) for an authorised medicinal product;
   - Investigator's Brochure for an unauthorised medicinal product.

The sponsor will report expedited the following SUSARs through the web portal *ToetsingOnline* to the METC:

– SUSARs that have arisen in the clinical trial that was assessed by the METC;
– SUSARs that have arisen in other clinical trials of the same sponsor and with the same medicinal product, and that could have consequences for the safety of the subjects involved in the clinical trial that was assessed by the METC.

The remaining SUSARs are recorded in an overview list (line-listing) that will be submitted once every half year to the METC. This line-listing provides an overview of all SUSARs from the study medicine, accompanied by a brief report highlighting the main points of concern. The expedited reporting of SUSARs through the web portal Eudravigilance or ToetsingOnline is sufficient as notification to the competent authority.

The sponsor will report expedited all SUSARs to the competent authorities in other Member States, according to the requirements of the Member States.

The expedited reporting will occur not later than 15 days after the sponsor has first knowledge of the adverse reactions. For fatal or life threatening cases the term will be maximal 7 days for a preliminary report with another 8 days for completion of the report.

9.3. Annual safety report

In addition to the expedited reporting of SUSARs, the sponsor will submit, once a year throughout the clinical trial, a safety report to the accredited METC, competent authority, and competent authorities of the concerned Member States.

This safety report consists of:

- a list of all suspected (unexpected or expected) serious adverse reactions, along with an aggregated summary table of all reported serious adverse reactions, ordered by organ system, per study;
- a report concerning the safety of the subjects, consisting of a complete safety analysis and an evaluation of the balance between the efficacy and the harmfulness of the medicine under investigation.

9.4. Follow-up of adverse events

All AEs will be followed until they have abated, or until a stable situation has been reached. Depending on the event, follow up may require additional tests or medical procedures as indicated, and/or referral to the general physician or a medical specialist.

SAEs need to be reported till end of study within the Netherlands, as defined in the protocol

9.5. Data Safety Monitoring Board (DSMB)

For this study, a Data Safety Monitoring Board (DSMB) has been assembled. The DSMB will review all deblinded data after every 4 subjects to review the quality of acquired data, as well as to ensure the safety of subjects. The DSMB consist of statistician Bjorn Winkens (UM) and cardiologists Christian Knackstadt (MUMC+) and Vanessa van Empel (MUMC+). All members of the DSMB have read the DSMB charter file (See Document K5) and have signed the competing interest form.

10. STATISTICAL ANALYSIS

Statistical analyses will be performed using SPSS for Mac iOS (IBM, version 23). All data will be tested for normality by visual inspection of data histograms and by means of Kolmogorov-Smirnov normality tests. When normally distributed, all data will be analysed with a paired Students T-test to analyse statistical differences between clenbuterol vs. placebo groups. In case of non-normally distributed data, a non-parametrical Wilcoxon signed-ranked test will be performed. Differences will be considered statistically significant when tested two-sided $p < 0.05$.

Data will be presented as mean ± standard error of the mean in case of normally distributed data. In case of non-normally distributed data, data will be presented as median and 25% or 75%. The hypothesis being tested during this study is:

H_0: $\mu_{placebo} = \mu_{clenbuterol}$

H_A: $\mu_{placebo} \neq \mu_{clenbuterol}$

During the study, only data from subjects who completed both study periods will be included in the data analysis. Missing values will not be replaced and, if a data for a specific parameter is missing, the subject in question will not contribute to the analysis of said parameter. The missing value policy was chosen due to the per-protocol statistical analysis of the study.

10.1. Primary study parameter(s)

The primary outcome parameter of this study is insulin-stimulated ^{18}F-FDG uptake in quadriceps muscle (expressed as Ki (min^{-1})) during the PET-MRI. This parameter is a numerical variable and will be expressed as mean ± standard error, minimum and maximum. Statistical analyses of glucose disposal rate will be performed by means of a paired students' T-test or Wilcoxon signed-ranked test based on the normality.

10.2. Secondary study parameter(s)

The secondary parameter is a numerical variable and will be presented as mean ± standard error, minimum and maximum or median, based on the normality of the data. The secondary parameter will be analysed by

means of a Students' T-test of Wilcoxon signed-rank test. Correlations between primary and secondary parameters will be performed with a Pearson's R correlation for normally distributed data. A Spearman's correlation test will be applied for non-normally distributed data.

10.3. Other study parameters

Subject characteristics will not be statistically analysed and will merely be presented as minimum, maximum, mean and standard error of the mean. Results acquired at the beginning, half-way and end of a supplementation period will be compared by means of a two-way ANOVA with a Bonferonni post-hoc test. This is performed to statistically analyse effects of clenbuterol/placebo supplementation on different variables upon acute and prolonged supplementation.

10.4. Interim analysis (if applicable)

N.A.

11. ETHICAL CONSIDERATIONS

11.1. Regulation statement

This study will be conducted according to the principles of the Declaration of Helsinki (64th WMA General Assembly, Fortaleza, Brazil, October 2013) and in accordance with the Medical Research Involving Human Subjects Act (WMO). The study will be conducted in compliance with International Conference on Harmonization Good Clinical Practice. The study will be approved by local medical ethics committee and local authorities before start of the study.

11.2. Recruitment and consent

Subjects will be recruited in Maastricht and surroundings by means of posters and advertisements in local newspapers and online (See document E3). Also, subjects that previously participated in other studies at the Department of Nutrition and Movement Sciences at Maastricht University or were screened and found not eligible to participate in other studies will be approached by means of a standardized email providing a short explanation of the study design (See Document K6). This type of recruitment will only occur if subjects indicated that they want to be approached for participation in future studies (signed consent) and appear to fulfill the inclusion criteria. The researchers will contact individuals who are interested in participating in the study by telephone, only after the individual has sought contact with the researcher on his or her own accord first. By contacting responders by telephone first, the burden of travel and time effort is reduced for the potential subject and researchers. In the telephone interview, the goal of the study will be explained, and the basic inclusion criteria will be discussed. When responders are interested, they will receive detailed subject information via e-mail or mail about participating in a medical study. They will be instructed to read this information carefully and to ask questions if things are unclear. These questions can be directed towards the researcher or towards the independent expert. The researcher will contact the possible participant again by email at least 7 days after the study information was received by the participant. If responders want to participate after reading the study information and seem to be eligible, they are invited for a screening. The informed consent form will be signed by both the researcher and subject on the day of screening prior to screening measurements will be performed.

11.3. Objection by minors or incapacitated subjects (if applicable)

Not applicable.

11.4. Benefits and risks assessment, group relatedness

Participation in this study will not result in any health benefits. During this study, the effect of clenbuterol vs. placebo will be investigated within the same subjects due to the cross-over design. The main benefit of this study is the acquired knowledge regarding the role of beta-2 adrenergic receptors in skeletal muscle and BAT glucose uptake and whole-body glucose homeostasis in general. Furthermore, this study could stimulate the development of novel beta-2 adrenergic ligands by pharmaceutical companies to prevent and treat T2DM.

Participation to this study will pose an intermediate risk to subject's health. The main burdens for the subjects are: time spend to participate in the study, potential adverse effects of clenbuterol, invasive sample collection, non-invasive measurements and unexpected medical findings. These burdens will be discussed into more detailed below.

Time spend to participate in the study

During this study, subjects will visit the University of Maastricht at 6 different occasions (excl. screening) in a period of 3-4 months. The total time which will be spend to the study is approximately 42.5 hours, which is excluding travelling time. To minimize this burden, the visits to the University will be carefully planned together with the subject.

Potential adverse effects of clenbuterol

Clenbuterol could potentially induce side effects, including headache, dizziness, heartburn, tremor, muscle ache and spasms. For a full list of all side effects, please see the SPC of clenbuterol in Document D2.

Invasive sample collection

During this study, several invasive sample collections will be performed, including blood sample collection, muscle biopsies and the hyperinsulinemic-euglycemic clamp. These measurements could be associated with local hematoma or bruise development. However, due to the state-of-the-art techniques, risks for infection or prolonged bleeding will be minimized. To further minimize these risks, pressure bandage will be placed which will have to be worn by the subject for at least 24 hours and subjects are recommended to refrain from any intensive exercise or heavy lifting. Within a couple of days, bruises will disappear. Finally, the muscle biopsy will be performed by an experienced medical doctor.

Non-invasive measurements

Blood pressure and heart rate measurements will be performed by means of an automatic inflatable cuff. These measurements might feel a bit uncomfortable, but do not induce detrimental health effects. In addition, the subject will have to lie in a PET-MRI scanner for a total of 150 minutes (75 minutes per study arm). During the imaging measurement, the noise may be somewhat unpleasant, but participants are provided with earplugs and/or headphones.

MRI is a safe procedure, with no known health risks as long as none of the exclusion criteria are met

Radiation

For the PET application, a radioactive tracer (^{18}F-FDG) will be injected (1.0 MBq/kg, for a 75 kg person = 75 MBq x 0.018 mSv = ~1.35 mSv radiation per injection) prior to the scan. Since this scanning protocol will be executed in two different conditions the total radiation dose for this study is 2 x 1.35 = 2.7 mSv. This is considered as a low risk (as stated by the Annals of ICRP 32), comparable to the background radiation received in one year. The effective dose is between 1-10 mSv (risk category <10-4), which is recommended and acceptable for research conducted with healthy adults and has been approved of by the Medical Ethics Committee in an earlier study by our group (see also METC10-3-027).

Unexpected medical findings

During the screening and measurements of the study, unexpected medical findings might be found. Subjects will always be informed regarding unexpected findings and this information will also be communicated to the general physician. If a subject does not want to be informed about unexpected medical findings, the subject is not allowed to participate.

Covid-19 statement

During the course of the study, several precautions have been put in place to limit the risk of covid-19 infection. First, upon entry of the facility, both subjects and researchers will disinfect their hands with hand sanitizer and will wear face masks (medical or non-medical) in all public spaces (i.e. hallways). Second, all researchers and subjects that enter the facility will be logged in a logbook, to allow a contact investigation if necessary. Within the MRUM facility, a walking designated route has been put in place to limit exposure to other individuals. The researcher will guide the subject directly to the clinical research room and will open all doors for the subjects. A distance of 1.5m will be maintained to the subject where possible. Inside the measurement rooms, it is allowed for the subject to remove their face mask whereas the researchers will always have theirs on. Following a measurement, the room will be thoroughly cleaned with alcohol wipes

and tubes of the indirect calorimetry will be bathed in decon for at least 30 minutes for disinfection. At last, all rooms will be ventilated for at least 30 minutes.

Risk-benefit assessment

The current study could potentially contribute to the development of a novel class of medication to prevent and treat T2DM. Since the prevalence of T2DM is reaching epidemic levels, we believe that the risks and time investment of this study our outweighed by the benefits.

11.5. Compensation for injury

The sponsor/investigator has a liability insurance which is in accordance with article 7 of the WMO. The sponsor also has an insurance which is in accordance with the legal requirements in the Netherlands (Article 7 WMO). This insurance provides cover for damage to research subjects through injury or death caused by the study.
The insurance applies to the damage that becomes apparent during the study or within 4 years after the end of the study.

1. € 650.000,-- (i.e. six hundred and fifty thousand Euro) for death or injury for each subject who participates in the Research;
2. € 5.000.000,-- (i.e. five million Euro) for death or injury for all subjects who participate in the Research;
3. € 7.500.000,-- (i.e. seven million and five hundred thousand Euro) for the total damage incurred by the organisation for all damage disclosed by scientific research for the Sponsor as 'verrichter' in the meaning of said Act in each year of insurance coverage.

11.6. Incentives (if applicable)

Subjects will receive a financial compensation of €620 for completing the study (See Attachment J1). In case of premature termination of the study, a reduced fee will be transferred which is dependent on the time investment and invasive procedures performed. The screening will not be compensated for since this could provide beneficial information for the participant. Compensation of travel costs will be made for all subjects

participating in the study, including the screening. This compensation will be maximally 19 eurocent per kilometre when travelling by car and complete cover of expenses when travelling by public transport.

12. ADMINISTRATIVE ASPECTS, MONITORING AND PUBLICATION

12.1. Handling and storage of data and documents

All information which will be obtained from the subject prior to and during the study will be kept private and protected. At the start of the study, subjects will be given an code that will not change during the study. The code consists of the study acronym and a two-digit number (i.e. CLB01). For all purposes this code will be used for participant identification. In a password protected file, this code is linked to the name, date of birth, address, and telephone number of the participant. This file can only be accessed by the research team (Drs. Pip van Lier and Dr. Joris Hoeks). The privacy of the participants who take part in the study will be protected. This means that the study code will not contain the participants' initials or birth date as explained above. These data will be stored for 15 years. In addition, data acquired during the test days will be stored in the researchers room in a closet with a lock.

The analyses of documents, data or samples outside the scope of this project will always require the permission of the subject. However, when collected data and samples obtained during the study can be applied during future studies within the scope of the study (i.e. investigating the effects of beta-2 agonists on glucose homeostasis), no permission will be asked.

Subjects will be given the opportunity to obtain information regarding their personal study results either verbally or in writing. This will always be performed in accordance to the privacy statement (Algemene Verordening Gegevensbescherming, AVG). In case of chance findings obtained during the screening (including elevated blood pressure or abnormal blood plasma values), these events will be noted in the CRF of the subject. In collaboration with the dependent physician, suitable action will be taken.

All samples collected during this study will be stored for 15 years. Storage of muscle biopsies will be performed in the -80 degrees freezers at the Department of Nutrition and Movement Sciences at Maastricht university, whereas blood samples will be stored at the BioBank Maastricht University Medical Centre+. Approval will be asked during the informed consent and if subjects decline the storage of the material, they will not be allowed to participate in the study. After 15 years, all the study material will be destroyed. Until then, only the research team, IGJ, DSMB and monitors have access to the research data and documents.

Follow up research

Subjects will be asked permission to be added to a subject list. We ask their permission to add the data (name, birth date, contact information, height, weight, and diabetes status) to a list that can be used to approach participants for follow-up research. This data will only be available for researchers from the department Nutrition and Movement Sciences. The administrator of the subject list (Yvonne Op den Kamp – Bruls) can create a list of potential candidates for the new study of a researcher upon his/her request. In this way, it is easier to approach participants for future studies. The participants can indicate in the informed consent whether they want to be added to this subject list. They can also decide not to share some data. They can also indicate if they no longer want to receive information about ongoing studies. Participants can edit their data or have it removed from the subject list anytime by contact the administrator by email (proefpersoon@dmrg.nl) or phone (06-40 60 56 22). To prevent the data, remain in the list if the participants do not want this, they will be asked annually (via email) whether their data may remain in the subject list. At their request, or after no response for two years, the data will be deleted. Subjects could contact researcher(s) or the subject list administrator for any questions regarding the subject list.

12.2. Monitoring and Quality Assurance

An independent monitor from the Clinical Trials Centre Maastricht (CTCM) will monitor all data obtained during the study. In addition, the DSMB will receive access to all data of the trial.

12.3. Amendments

A ‘substantial amendment’ is defined as an amendment to the terms of the METC application, or to the protocol or any other supporting documentation, that is likely to affect to a significant degree:

- the safety or physical or mental integrity of the subjects of the trial;
- the scientific value of the trial;
- the conduct or management of the trial; or
- the quality or safety of any intervention used in the trial.

All substantial amendments will be notified to the METC and to the competent authority.

Non-substantial amendments will not be notified to the accredited METC and the competent authority, but will be recorded and filed by the sponsor.

12.4. Annual progress report

The sponsor/investigator will submit a summary of the progress of the trial to the accredited METC once a year. Information will be provided on the date of inclusion of the first subject, numbers of subjects included and numbers of subjects that have completed the trial, serious adverse events/ serious adverse reactions, other problems, and amendments.

12.5. Temporary halt and (prematurely) end of study report

The sponsor will notify the accredited METC and the competent authority of the end of the study within a period of 90 days. The end of the study is defined as the last patient's last visit.
The sponsor will notify the METC immediately of a temporary halt of the study, including the reason of such an action.

In case the study is ended prematurely, the sponsor will notify the accredited METC and the competent authority within 15 days, including the reasons for the premature termination.
Within one year after the end of the study, the investigator/sponsor will submit a final study report with the results of the study, including any publications/abstracts of the study, to the accredited METC and the Competent Authority.

12.6. Public disclosure and publication policy

Both positive and negative results of this study will be published in peer-reviewed scientific journals. The publication policy is in agreement with the CCMO publication statement and with the Eurostars Programme.

13. STRUCTURED RISK ANALYSIS

13.1. Potential issues of concern

a. Level of knowledge about mechanism of action

It is currently unknown whether supplementation with a selective β_2-agonist enhances *in vivo* skeletal muscle glucose uptake through the mTORC2 pathway in obese humans. However, we have compelling evidence from both *in vitro* and pre-clinical *in vivo* models that supplementation with a β_2-agonist beneficially affects skeletal muscle glucose uptake, and glucose homeostasis, through the activation of the mTORC2 pathway (See Chapter 1 Introduction), thereby strengthening our working hypothesis.

b. Previous exposure of human beings with the test product(s) and/or products with a similar biological mechanism

Clenbuterol hydrochloride is used as an asthma medication in several European countries and effects have been reported in several peer-reviewed articles. Furthermore, extensive research has been performed on the safety and pharmacokinetics of the medicine (See Document D2). These studies have all been performed on patients with obstructive airway diseases. In addition, we have shown that clenbuterol – at the same dose applied during this study – is well-tolerated in young male adults.

c. Can the primary or secondary mechanism be induced in animals and/or in *ex-vivo* human cell material?

Multiple studies have shown an increase in *in vitro* skeletal muscle glucose uptake upon β_2-agonist treatment (33-36), although the precise underlying molecular mechanisms remained unclear. Recently, it has been shown that incubation with clenbuterol significantly enhanced glucose uptake in both cultured L6 muscle cells and human primary myotubes through the activation of mTORC2 (3). In addition, clenbuterol supplementation (30 mg/L in the drinking water) significantly improved glucose tolerance in both high-fat diet-induced obese mice and Goto-Kakizaki rats, two-well established animal models for diabetes (3, 8, 9). In addition, these beneficial effects were abolished in β_1/β_2-knockout mice, indicating the physiological relevance of this pathway (3). These effects were also observed upon a low-dose of clenbuterol supplementation (See Chapter 1 introduction).

d. Selectivity of the mechanism to target tissue in animals and/or human beings

The beneficial effects seen in rodents are most likely mediated by the skeletal muscle, which has a high abundancy of the β_2-adrenergic receptor (4, 5). For further information regarding the toxicology, please see the SPC of clenbuterol (Document D2). Besides the skeletal muscle, BAT could potentially be activated upon prolonged clenbuterol supplementation. BAT is a highly energetic tissue which is able to consume large amounts of glucose. In turn, this could beneficially affect whole-body glucose homeostasis.

e. Analysis of potential effect

The daily recommended dose of clenbuterol is between 40 and 80 µg/day. The dose used during this study falls within the daily recommended dose and, thereby, does not induce toxic doses. The effect of clenbuterol on skeletal muscle glucose uptake is known, in mice, to be dose-dependent (8). Common side effects do not pose a hazard for overall health and at the current dose well-tolerated by humans. For further detailed information regarding side effects, please see SPC text of clenbuterol.

f. Pharmacokinetic considerations

Clenbuterol hydrochloride is rapidly and fully absorbed after oral administration in the gastrointestinal region. Maximal plasma concentrations are achieved within 2-3 hours after ingestion (with maximal concentrations of 0.2 ng/mL with an acute 40 µg intake) and is distributed evenly throughout the tissues. Binding to plasma proteins is 45-68%. Upon prolonged clenbuterol supplementation, plasma levels reach a plateau within 4 days after initial administration (plateau: 0.5-0.6 ng/mL with 40 µg/day) (22).
The metabolism of clenbuterol is performed, to a lesser extent, by the liver. In total, 8 metabolites have been found which do not have any pharmacological activity. The excretion of clenbuterol occurs in two phases. The T_{50} of the first phase is 1 hour, whereas the second phase is 34 hours. The main route of excretion is through renal excretion in an unaltered form (87% within 168 hours post dose). Within 168 hours, approximately 97% of the administered dose is excreted.

g. Study population

The study population will consist of overweight/obese (BMI: 25-35 kg/m^2) male and (postmenopausal) female subjects aged between 40-75 years old.

h. Interaction with other products

Please see SPC text of Clenbuterol (Document D2).

i. Predictability of effect

Please see SPC text of Clenbuterol (Document D2).

j. Can effects be managed?

Any side effects that emerge with clenbuterol supplementation will disappear upon withdrawal. Participants will receive a card with the relevant information of the study that a physician needs to know in case of an emergency (See Document F3). Furthermore, the physician can, in case of emergency, contact the independent physician (Dr. Ronald Henry), dependent medical physician (Dr. T. Weijer van de), the secretary of the department of Nutrition and Movement Sciences (Yolanda Verhaegen) or the principle investigator (Dr. J. Hoeks) for the unblinding key to decipher whether the subject has clenbuterol or placebo supplementation. In less emergent situations, the subject can contact the researcher (Pip van Lier) or the principle investigator (Dr J. Hoeks) during working hours. In that case, the responsible medical doctor will be contacted to decide whether the subject should be excluded from the study.

13.2. Synthesis

The risks of the measurements during this study are low which is highlighted by the low prevalence of adverse effects. This is mainly due to the state-of-the-art techniques that are applied and the sterile working methods. In addition, the burden of time investment is relatively low (~42.5 hours spread over 3-4 months including two overnight stays). Clenbuterol hydrochloride is an asthma medication that is used in several European countries. Extensive research has been performed on the safety and pharmacokinetics of the drug. At the dose currently applied in this study (40 µg/day), the use of clenbuterol hydrochloride is generally accepted as safe. Nevertheless, the use of clenbuterol hydrochloride can induce potential side-effects, as listed in the patient leaflet (Document D2).

However, within the clinical range, these adverse effects are minimal and these effects will disappear upon discontinuation of the supplement. To further reduce the risk of adverse effects, we deliberately choose for a standard dose, which we have experienced to be safe for clinical use. In addition, subjects will receive a radiation burden of ~2.7 mSv (normal background radiation in the Netherlands ~2.5 mSv). We believe that these risks are acceptable for the subjects since this study can potentially open fundamentally new therapeutic methods to treat insulin resistance in T2DM.

14. REFERENCES

1. DeFronzo RA, Gunnarsson R, Bjorkman O, Olsson M, Wahren J. Effects of insulin on peripheral and splanchnic glucose metabolism in noninsulin-dependent (type II) diabetes mellitus. J Clin Invest. 1985;76(1):149-55.
2. Czech MP. Insulin action and resistance in obesity and type 2 diabetes. Nat Med. 2017;23(7):804-14.
3. Sato M, Dehvari N, Oberg AI, Dallner OS, Sandstrom AL, Olsen JM, et al. Improving type 2 diabetes through a distinct adrenergic signaling pathway involving mTORC2 that mediates glucose uptake in skeletal muscle. Diabetes. 2014;63(12):4115-29.
4. Liggett SB, Shah SD, Cryer PE. Characterization of beta-adrenergic receptors of human skeletal muscle obtained by needle biopsy. Am J Physiol. 1988;254(6 Pt 1):E795-8.
5. Sillence MN, Matthews ML. Classical and atypical binding sites for beta-adrenoceptor ligands and activation of adenylyl cyclase in bovine skeletal muscle and adipose tissue membranes. Br J Pharmacol. 1994;111(3):866-72.
6. Castle A, Yaspelkis BB, 3rd, Kuo CH, Ivy JL. Attenuation of insulin resistance by chronic beta2-adrenergic agonist treatment possible muscle specific contributions. Life Sci. 2001;69(5):599-611.
7. Pan SJ, Hancock J, Ding Z, Fogt D, Lee M, Ivy JL. Effects of clenbuterol on insulin resistance in conscious obese Zucker rats. Am J Physiol Endocrinol Metab. 2001;280(4):E554-61.
8. Kalinovich A, Dehvari N, Aslund A, van Beek S, Halleskog C, Olsen J, et al. Treatment with a beta-2-adrenoceptor agonist stimulates glucose uptake in skeletal muscle and improves glucose homeostasis, insulin resistance and hepatic steatosis in mice with diet-induced obesity. Diabetologia. 2020;63(8):1603-15.
9. van Beek S, Kalinovich A, Schaart G, Bengtsson T, Hoeks J. Prolonged beta2-adrenergic agonist treatment improves glucose homeostasis in diet-induced obese UCP1(-/-) mice. Am J Physiol Endocrinol Metab. 2021.
10. Jessen S, Solheim SA, Jacobson GA, Eibye K, Bangsbo J, Nordsborg NB, et al. Beta2 -adrenergic agonist clenbuterol increases energy expenditure and fat oxidation, and induces mTOR phosphorylation in skeletal muscle of young healthy men. Drug Test Anal. 2020;12(5):610-8.
11. Cannon B, Nedergaard J. Brown adipose tissue: function and physiological significance. Physiol Rev. 2004;84(1):277-359.
12. Orava J, Nuutila P, Lidell ME, Oikonen V, Noponen T, Viljanen T, et al. Different metabolic responses of human brown adipose tissue to activation by cold and insulin. Cell Metab. 2011;14(2):272-9.

13.Shibata H, Perusse F, Vallerand A, Bukowiecki LJ. Cold exposure reverses inhibitory effects of fasting on peripheral glucose uptake in rats. Am J Physiol. 1989;257(1 Pt 2):R96-101.
14.Bartelt A, Bruns OT, Reimer R, Hohenberg H, Ittrich H, Peldschus K, et al. Brown adipose tissue activity controls triglyceride clearance. Nat Med. 2011;17(2):200-5.
15.Olsen JM, Csikasz RI, Dehvari N, Lu L, Sandstrom A, Oberg AI, et al. beta3-Adrenergically induced glucose uptake in brown adipose tissue is independent of UCP1 presence or activity: Mediation through the mTOR pathway. Mol Metab. 2017;6(6):611-9.
16.Vallerand AL, Lupien J, Bukowiecki LJ. Cold exposure reverses the diabetogenic effects of high-fat feeding. Diabetes. 1986;35(3):329-34.
17.Xiao C, Goldgof M, Gavrilova O, Reitman ML. Anti-obesity and metabolic efficacy of the beta3-adrenergic agonist, CL316243, in mice at thermoneutrality compared to 22 degrees C. Obesity (Silver Spring). 2015;23(7):1450-9.
18.Liu X, Wang S, You Y, Meng M, Zheng Z, Dong M, et al. Brown Adipose Tissue Transplantation Reverses Obesity in Ob/Ob Mice. Endocrinology. 2015;156(7):2461-9.
19.Stanford KI, Middelbeek RJ, Townsend KL, An D, Nygaard EB, Hitchcox KM, et al. Brown adipose tissue regulates glucose homeostasis and insulin sensitivity. J Clin Invest. 2013;123(1):215-23.
20.Cypess AM, Weiner LS, Roberts-Toler C, Franquet Elia E, Kessler SH, Kahn PA, et al. Activation of human brown adipose tissue by a beta3-adrenergic receptor agonist. Cell Metab. 2015;21(1):33-8.
21.Blondin DP, Nielsen S, Kuipers EN, Severinsen MC, Jensen VH, Miard S, et al. Human Brown Adipocyte Thermogenesis Is Driven by beta2-AR Stimulation. Cell Metab. 2020;32(2):287-300 e7.
22.Yamamoto I, Iwata K, Nakashima M. Pharmacokinetics of plasma and urine clenbuterol in man, rat, and rabbit. J Pharmacobiodyn. 1985;8(5):385-91.
23.Kamalakkannan G, Petrilli CM, George I, LaManca J, McLaughlin BT, Shane E, et al. Clenbuterol increases lean muscle mass but not endurance in patients with chronic heart failure. J Heart Lung Transplant. 2008;27(4):457-61.
24.Johansson E, Lubberink M, Heurling K, Eriksson JW, Skrtic S, Ahlstrom H, et al. Whole-Body Imaging of Tissue-specific Insulin Sensitivity and Body Composition by Using an Integrated PET/MR System: A Feasibility Study. Radiology. 2018;286(1):271-8.
25.Su WJ, Perng RP. Spiropent (clenbuterol): another choice for patients with chronic reversible airways obstruction. Zhonghua Yi Xue Za Zhi (Taipei). 1991;47(1):13-7.
26.Wheatley D. Clenbuterol ("Spiropent'): a long-acting bronchodilator. Curr Med Res Opin. 1982;8(2):113-9.
27.Pasotti C, Capra A, Vibelli C. NAB 365 (clenbuterol) and salbutamol in asthmatics: a double-blind clinical trial. Int J Clin Pharmacol Biopharm. 1979;17(4):176-80.

28. Baronti A, Grieco A, Vibelli C. Oral NAB 365 (clenbuterol) and terbutaline in chronic obstructive lung disease: a double-blind, two-week study. Int J Clin Pharmacol Ther Toxicol. 1980;18(1):21-5.
29. Salorinne Y, Stenius B, Tukiainen P, Poppius H. Double-blind cross-over comparison of clenbuterol and salbutamol tablets in asthmatic out-patients. Eur J Clin Pharmacol. 1975;8(3-4):189-95.
30. Al-Majed AA, Khalil NY, Khbrani I, Abdel-Aziz HA. Clenbuterol Hydrochloride. Profiles Drug Subst Excip Relat Methodol. 2017;42:91-123.
31. Bergstrom J, Hermansen L, Hultman E, Saltin B. Diet, muscle glycogen and physical performance. Acta Physiol Scand. 1967;71(2):140-50.
32. ICRP. Annals of the ICRP. Risks associated with ionizing radiation. Pergamon Press. 1992.
33. Ngala RA, O'Dowd J, Wang SJ, Agarwal A, Stocker C, Cawthorne MA, et al. Metabolic responses to BRL37344 and clenbuterol in soleus muscle and C2C12 cells via different atypical pharmacologies and beta2-adrenoceptor mechanisms. Br J Pharmacol. 2008;155(3):395-406.
34. Ngala RA, O'Dowd J, Wang SJ, Stocker C, Cawthorne MA, Arch JR. Beta2-adrenoceptors and non-beta-adrenoceptors mediate effects of BRL37344 and clenbuterol on glucose uptake in soleus muscle: studies using knockout mice. Br J Pharmacol. 2009;158(7):1676-82.
35. Nevzorova J, Bengtsson T, Evans BA, Summers RJ. Characterization of the beta-adrenoceptor subtype involved in mediation of glucose transport in L6 cells. Br J Pharmacol. 2002;137(1):9-18.
36. Nevzorova J, Evans BA, Bengtsson T, Summers RJ. Multiple signalling pathways involved in beta2-adrenoceptor-mediated glucose uptake in rat skeletal muscle cells. Br J Pharmacol. 2006;147(4):446-54.
