# Supplementary material for: Prolonged β2-agonist treatment enhances muscle-specific glucose uptake in individuals with overweight and obesity: a randomized placebo-controlled trial
Source: Nat Commun. 2026 Apr 21;17:5483. doi: 10.1038/s41467-026-71897-9 (PMC13284196; doi:10.1038/s41467-026-71897-9)
Supplement: Supplementary file 2 — Reporting summary [file 41467_2026_71897_MOESM2_ESM.pdf]

nature portfolio

Corresponding author(s): Pip van Lier, Joris Hoeks

Last updated by author(s): 05MAR2026

Reporting Summary

Nature Portfolio wishes to improve the reproducibility of the work that we publish. This form provides structure for consistency and transparency in reporting. For further information on Nature Portfolio policies, see our Editorial Policies and the Editorial Policy Checklist.

Statistics

For all statistical analyses, confirm that the following items are present in the figure legend, table legend, main text, or Methods section.

n/a	Confirmed	
☐	☒	The exact sample size (n) for each experimental group/condition, given as a discrete number and unit of measurement
☐	☒	A statement on whether measurements were taken from distinct samples or whether the same sample was measured repeatedly
☐	☒	The statistical test(s) used AND whether they are one- or two-sided<br>*Only common tests should be described solely by name; describe more complex techniques in the Methods section.*
☒	☐	A description of all covariates tested
☐	☒	A description of any assumptions or corrections, such as tests of normality and adjustment for multiple comparisons
☐	☒	A full description of the statistical parameters including central tendency (e.g. means) or other basic estimates (e.g. regression coefficient) AND variation (e.g. standard deviation) or associated estimates of uncertainty (e.g. confidence intervals)
☐	☒	For null hypothesis testing, the test statistic (e.g. F, t, r) with confidence intervals, effect sizes, degrees of freedom and P value noted<br>*Give P values as exact values whenever suitable.*
☒	☐	For Bayesian analysis, information on the choice of priors and Markov chain Monte Carlo settings
☒	☐	For hierarchical and complex designs, identification of the appropriate level for tests and full reporting of outcomes
☐	☒	Estimates of effect sizes (e.g. Cohen's d, Pearson's r), indicating how they were calculated

Our web collection on statistics for biologists contains articles on many of the points above.

Software and code

Policy information about availability of computer code

Data collection	N/A
Data analysis	PMOD software (version 3.7, PMOD technologies, Zurich, Switzerland) for tissue-specific MRgluc measurements. Static BAT glucose uptake was analysed using Siemens Syngo.via Client 10.4 (x64) VB60I_HF01, Siemens syngo.via Bootstrapper 8.0 (Siemens Healthcare). FMD measurements were analysed with Matlab software (MyFMD 2015, developed by Professor A.P.G. Hoeks, Department of Biomedical Engineering, Maastricht University, The Netherlands). The adrenal gland volume is measured semi-automatically with MIMvista software (MIM 7.3.7) on T2 weighted images acquired during PET-MRI scanning, which are used for anatomical correlation.

For manuscripts utilizing custom algorithms or software that are central to the research but not yet described in published literature, software must be made available to editors and reviewers. We strongly encourage code deposition in a community repository (e.g. GitHub). See the Nature Portfolio guidelines for submitting code & software for further information.

Data

Policy information about availability of data

All manuscripts must include a data availability statement. This statement should provide the following information, where applicable:

- Accession codes, unique identifiers, or web links for publicly available datasets
- A description of any restrictions on data availability
- For clinical datasets or third party data, please ensure that the statement adheres to our policy

Source data are provided in this paper. Upon reasonable scientific request, de-identified and processed participant data can be requested from the corresponding author (j.hoeks@maastrichtuniversity.nl), with no end date, folllowing the completion of a signed data transfer agreement. De-identified data will be shared due to participant privacy.

Research involving human participants, their data, or biological material

Policy information about studies with human participants or human data. See also policy information about sex, gender (identity/presentation), and sexual orientation and race, ethnicity and racism.

Reporting on sex and gender	In the study, both males and females were included. Sex and/or gender was recorded based on self-report. We did not focus on sex- or gender-specific differences in the outcome parameters as the study assessed individual changes as compared to a between-group analysis. Participants of both sexes were included to improve the generalizability of the findings. However, the study was not powered to detect sex-specific effects, and the sample size was insufficient to support reliable stratified analyses
Reporting on race, ethnicity, or other socially relevant groupings	All participants were white western European individuals
Population characteristics	Participants were aged 40-75 years, BMI 25-35 kg/m2, male or (postmenopausal) female.
Recruitment	Recruitment was conducted in Maastricht and its direct surroundings through flyers and online advertisements. Informed consent was obtained from all participants prior to participation.
Ethics oversight	The study was reviewed and approved by the Medical Ethics Committee azM/UM.

Note that full information on the approval of the study protocol must also be provided in the manuscript.

Field-specific reporting

Please select the one below that is the best fit for your research. If you are not sure, read the appropriate sections before making your selection.

☒ Life sciences ☐ Behavioural & social sciences ☐ Ecological, evolutionary & environmental sciences

For a reference copy of the document with all sections, see nature.com/documents/nr-reporting-summary-flat.pdf

Life sciences study design

All studies must disclose on these points even when the disclosure is negative.

Sample size	The sample size was based on a previous study, in which 18F-FDG uptake was assessed in thigh muscle of healthy, overweight individuals averaged 0.0142 mL/cm3/min with a standard deviation of 0.0037 mL/cm3/min during a constant insulin infusion (Johansson et al. 2018). Assuming a physiologically relevant 20% increase in muscle glucose uptake, 80% power, and a Type I error probability of 0.05, a power calculation based on a paired sample T-test indicated that in total 14 participants were needed to complete the study.
Data exclusions	The number of participants included in the analyses differs by tissue type. For skeletal muscle, one or two participants were excluded from the analysis due to inadequate image quality (e.g. noise, movement artefacts) (vastus lateralis: n=12; hamstring: n=13). One participant could not complete the entire scan procedure and was therefore excluded from the BAT analysis (n=13). For liver (n=13) and heart (n=13) only one participant was excluded from the analysis due to inadequate image quality.<br><br>Respiratory exchange ratio (RER) data was excluded for one participant due to technical issues with the respiration chamber overnight (n=13). For non-protein RER (nPRER) and substrate oxidation analyses, two participants were excluded due to failed urine collection, which prevented nitrogen analysis and subsequent corrections for protein, fat, carbohydrate oxidation, and nocturnal energy expenditure (n=12).<br><br>One participant was excluded from free fatty acid analyses due to poor sample quality (n=13). One participant was excluded from the C-peptide analysis due to a missing blood sample, resulting in a final sample size of 13 (n = 13).<br><br>Due to scheduling issues, FMD data is lacking for one participant (n=13). For the analysis of FMD corrected for peak velocity flow stimulus (pFMDv%), one additional participant was excluded because of a failed (peak velocity flow) stimulus assessment (n=12).

Replication	The primary and secondary outcomes were not replicated within subjects.
Randomization	Block randomization was performed in groups of four by an independent researcher to achieve random allocation in the study arms.
Blinding	This study was a double-blinded study

Reporting for specific materials, systems and methods

We require information from authors about some types of materials, experimental systems and methods used in many studies. Here, indicate whether each material, system or method listed is relevant to your study. If you are not sure if a list item applies to your research, read the appropriate section before selecting a response.

Materials & experimental systems

n/a	Involved in the study
☒	☐ Antibodies
☒	☐ Eukaryotic cell lines
☒	☐ Palaeontology and archaeology
☒	☐ Animals and other organisms
☐	☒ Clinical data
☒	☐ Dual use research of concern
☒	☐ Plants

Methods

n/a	Involved in the study
☒	☐ ChIP-seq
☒	☐ Flow cytometry
☒	☐ MRI-based neuroimaging

Clinical data

Policy information about clinical studies

All manuscripts should comply with the ICMJE guidelines for publication of clinical research and a completed CONSORT checklist must be included with all submissions.

Clinical trial registration	The research was registered on the 10th of June 2021 at ClinicalTrials.gov, carrying the identifier NCT04921306
Study protocol	The full trial protocol can be found in the supplementary information or can be requested from the corresponding author.
Data collection	The research data was collected at Maastricht University (Maastricht, The Netherlands) between the periods of November 1st 2022 and March 1st 2024.
Outcomes	The primary objective of this study was to assess the effect of 4 weeks of clenbuterol versus placebo treatment on tissue-specific insulin-stimulated glucose uptake, as reflected by MRgluc, in the quadriceps muscle. The secondary objective was defined as insulin-stimulated glucose uptake in brown adipose tissue (BAT), as reflected by the SUV. These outcomes were quantified using a hyperinsulinemic-euglycemic clamp combined with 18F-FDG PET-MRI.

Plants

Seed stocks	N/A
Novel plant genotypes	N/A
Authentication	N/A
