# Supplementary material for: Prolonged β2-agonist treatment enhances muscle-specific glucose uptake in individuals with overweight and obesity: a randomized placebo-controlled trial
Source: Nat Commun. 2026 Apr 21;17:5483. doi: 10.1038/s41467-026-71897-9 (PMC13284196; doi:10.1038/s41467-026-71897-9)
Supplement: Supplementary file 3 — Transparent Peer Review file [file 41467_2026_71897_MOESM3_ESM.pdf]

nature portfolio

Peer Review File

Prolonged β2-agonist treatment enhances muscle-specific glucose uptake in individuals with overweight and obesity: a randomized placebo-controlled trial

Corresponding Author: Ms Pip Lier

This file contains all reviewer reports in order by version, followed by all author rebuttals in order by version.

Version 0:

Reviewer comments:

Reviewer #1

(Remarks to the Author)
Van Lier and coworkers performed a randomized, placebo-controlled, double-blind, crossover study on the effects of 4-week clenbuterol treatment (40 µg/day) on tissue-specific insulin-stimulated glucose uptake in overweight and obese individuals at risk for type 2 diabetes mellitus (T2DM). Using 18F-FDG PET-MRI combined with hyperinsulinemic-euglycemic clamps, the authors demonstrated that clenbuterol enhanced insulin-stimulated glucose uptake in hamstring muscle and showed a trend toward improvement in vastus lateralis muscle. Clenbuterol did not affect brown adipose tissue (BAT) glucose uptake, whole-body insulin sensitivity, or fasting metabolic parameters. While these findings provide valuable translational evidence for β-adrenergic pathway modulation in glucose metabolism, several methodological and interpretive limitations impact the clinical significance of the results.

MAJOR COMMENTS
- The sample size is very small considering the heterogeneity of the population under investigation. The power calculation was based on an expected 20% improvement in glucose uptake, but the observed effects were smaller (13-15%). The Cohen's d values (0.282-0.441) indicate small to medium effect sizes, suggesting the study was underpowered for the actual effects observed. This raises questions about the clinical meaningfulness of the detected changes.
- The authors should provide a better rationale for choosing clenbuterol over more selective β2-agonists such as salbutamol or formoterol. Given the cardiovascular side effects observed and the known selectivity profiles of available β2-agonists, the choice of clenbuterol requires better justification, particularly in a population at risk for cardiovascular disease.
- The lack of improvement in whole-body insulin sensitivity, fasting glucose, or other systemic metabolic parameters raises questions about the clinical relevance of the muscle-specific improvements. The authors need to better address how localized muscle glucose uptake improvements without systemic benefits would translate to meaningful clinical outcomes in T2DM prevention or treatment.

MINOR COMMENTS
- The exclusion of T2DM individuals should be mentioned in the "Study participants" section rather than first appearing in the discussion.
- The manuscript should specify how many participants fell into overweight versus Class I obesity categories and whether outcomes differed by BMI subgroup.
- With only 3 females out of 14 participants, the generalizability of results is limited.
- All blood parameter values in Table 1 are reported without corresponding units.
- "Kalium" should be replaced with "Potassium" and "Hemoglobin" is misspelled.
- Plasma glucose values could be added to Table 1 if measured during screening.
- The relevance of measuring insulin's suppressive effect on fatty acids should be better explained.
- The authors should better address the apparent paradox of how β2-agonists could have therapeutic utility in T2DM treatment if they increase hepatic glucose production without changing fasting plasma glucose. This relates to the need for more selective β2-agonist profiles or weight-based dosing strategies.

Reviewer #2

(Remarks to the Author)
This study has examined the effects of a 4-week treatment with the β2-adrenergic agonist clenbuterol (40 μg/day) on insulin-stimulated metabolic rate of glucose (MRgluc) in the quadriceps muscle (primary outcome) and glucose uptake in brown adipose tissue (BAT) (secondary outcome) using 18F-FDG PET-MRI in fourteen individuals with overweight or obesity. The authors conclude that insulin-stimulated MRgluc tended to improve in vastus lateralis muscle (15%, p=0.072) and increased significantly in hamstring muscle (13%, p=0.037), while BAT glucose uptake remained unaffected and that these findings suggest potential therapeutic benefits of β2-AR agonists for improving skeletal muscle glucose uptake in individuals with (pre)diabetes.
In general this is a well conducted comprehensive clinical study by an experienced group using state-of-the-art methodology. I do however have major concerns as regards the design of the study and the interpretation of data.
It appears that clenbuterol was given twice daily together with morning and evening meals, the final dose being taken at 18.00 prior to the study day; this effectively implies that this by design is a post intervention study, which disregards and is not representative of metabolic phenomena occurring the previous 4 wks during active β-2 treatment. It is the metabolic events during treatment, which determines the suitability of the treatment, not withdrawal repercussions and it is generally well documented that other β2-adrenergic agonists like adrenaline are potent insulin antagonists glucose and lipid-wise and induces insulin resistance acutely – one study has reported that insulin stimulated glucose uptake in the adrenaline perfused human leg(including the vastus lateralis) acutely decreases > 80 % by A/V techniques(Gjedsted et al Acta Physiol, 2011 (& J Onslev et al, J Clin Endocrinol Metab. 2024)) and it is well described that adrenaline is a crucial component of the counterregulatory cascade and that up to 50% of patients with pheochromocytoma develop diabetes. Thus, if anything one would in parallel with all other stress hormones expect intermittent hyperglycemia and compensatory hyperinsulinemia during the active 4 wk treatment - continuous glucose – and lactate - monitoring or perhaps HbA1c would have been useful in this context. Another possibility is that 4 wks of active treatment inhibits endogenous catecholamine generation and tentatively induces refractory adrenal atrophy and low levels of endogenous catecolamines after intervention – it might be considered to measure urinary catecholamine excretion post-intervention. Given the cardiometabolic side effects of β-2 treatment and the vulnerability of patients with pre-diabetes/metabolic syndrome, most clinicians would be much reluctant to use such a remedy.
M-values are clearly the most important measure of WB insulin sensitivity and should be given in the abstract & numerically elsewhere – the conclusions seem a bit overstretched – if there is a minor improvement of insulin sensitivity in some muscles, there must by implication be deterioration in other tissues – where?
Target plasma glucose values during the clamp were high(6.0 - 6.5 mmol/L) – did the authors measure c-peptide during the clamp?

Reviewer #3

(Remarks to the Author)
General Comments

This manuscript reports a crossover trial examining whether β2-adrenergic receptor stimulation improves tissue-specific glucose clearance in skeletal muscle and brown adipose tissue (BAT), and whether this augments insulin sensitivity in individuals at risk of developing type 2 diabetes mellitus (T2DM). The research question is interesting and relevant; however, several methodological and analytical issues require clarification.

Sample Size Calculation

While the reported sample size may be sufficient, the rationale for using a paired t-test to estimate the required sample size is unclear. In a crossover design, the primary goal is typically to compare differences between treatment and control conditions rather than only within-subject changes. In much of the methodological literature, sample size estimation for crossover trials incorporates elements more consistent with independent samples t-tests [1,2] or online calculator https://www2.ccrb.cuhk.edu.hk/stat/mean/tsmc_sup.htm

Furthermore, if the authors assumed a mean difference (d) of 0.0142 with an SD of 0.0037, the implied effect size would be approximately 3.8, which would lead to an even smaller sample size than reported. I attempted a calculation using the pwr package in R, which supported this observation. The authors should clarify their assumptions and provide more detail on the sample size estimation approach.

Carryover Effect

Although the study included a 6–8 week washout period, it would be important to report whether potential carryover effects were formally assessed or accounted. This is a key concern in crossover trials and should be addressed explicitly.

Statistical Analysis

The data analysis section is currently very brief and states only that paired t-tests were used for normally distributed variables and Wilcoxon signed-rank tests for non-normal variables. As noted above, it is not clear whether a paired t-test is the most appropriate method for the primary analyses. More detail should be provided on the planned statistical approach, including whether more robust methods such as mixed-effects models (treating subjects as random effects) were considered. Such approaches are frequently recommended in crossover designs [2].

Results

In the results section, I noted descriptions such as:
"… although this did not reach statistical significance (11.43 ± 0.97 vs. 9.96 ± 0.96 μmol/min/100g, p=0.072, Cohen's d = 0.441; Fig 1a and b)…"
The reporting here suggests the use of independent samples t-tests, which seems inconsistent with the stated use of paired tests. The authors should clarify exactly which statistical tests were performed for each analysis and ensure alignment between the methods and results sections.

I think the results are clearly presented.

[1] Wellek, S., & Blettner, M. (2012). On the proper use of the crossover design in clinical trials: part 18 of a series on evaluation of scientific publications. Deutsches Arzteblatt international, 109(15), 276–281. https://doi.org/10.3238/arztebl.2012.0276
[2] Jones, B., & Kenward, M. G. (2003). Design and analysis of cross-over trials (2nd ed., Vol. 98;98.;). Chapman & Hall/CRC. [Chapter 2 and 5]

Version 1:

Reviewer comments:

Reviewer #2

(Remarks to the Author)
The ms has improved considerably - I still believe that it is important to underline that the design implies that the investigation is carried out in a transitional phase in which drug levels are declining and that generalisability is dubious

Reviewer #3

(Remarks to the Author)
The authors have addressed all my questions. I don't have further comments.

Reviewer #4

(Remarks to the Author)
Review Prolonged β2-agonist treatment enhances muscle-specific glucose uptake in individuals with overweight and obesity: a randomized placebo-controlled trial

This is a well conducted and timely mechanistic clinical trial investigating the effect of beta2-AR agonist clenbuterol on muscle glucose uptake. It uses state-of-the-art technology. The conclusions drawn by the authors are supported by the presented data.

As tasked I am reviewing the answers to the requests of Reviewer 1 who was not able to review the revised manuscript.

Major points:
Sample size and power.
- i agree with the authors that their sample size was adequate. Importantly, it should be noted that the project was designed to elucidate basic physiological / pharmacological mechanisms and not to re-purpose clenbuterol as a new diabetes medication. Furthermore, the question whether the power was too low would only be of relevance when no significant difference between active treatment and placebo was observed.

- choice of clenbuterol as beta2-agonists.
the authors satisfactorily justify their choice of medication. It should be highlighted that in this kind of investigator-initiated trials rely on the availability of medications. I.e. usually medications that are already approved and marketed have to be used.

- clinical relevance of the study
As commented above, the trial presented here was intended to elucidate basic mechanisms in humans and to guide further research. It was not a clinical trial intended to obtain approval for a new treatment indication. As such, Nature Communications is also a very suitable journal to present these data. The authors have adressed the reviewer's comment satisfactorily.

Minor points
- the authors have satisfactorily answered all minor points raised by the reviewer.

============================
I recommend to accept the manuscript for publication in Nature Communications.

Open Access This Peer Review File is licensed under a Creative Commons Attribution 4.0 International License, which permits use, sharing, adaptation, distribution and reproduction in any medium or format, as long as you give appropriate credit to the original author(s) and the source, provide a link to the Creative Commons license, and indicate if changes were made.
In cases where reviewers are anonymous, credit should be given to 'Anonymous Referee' and the source.
The images or other third party material in this Peer Review File are included in the article’s Creative Commons license, unless indicated otherwise in a credit line to the material. If material is not included in the article’s Creative Commons license and your intended use is not permitted by statutory regulation or exceeds the permitted use, you will need to obtain permission directly from the copyright holder.
To view a copy of this license, visit https://creativecommons.org/licenses/by/4.0/

Reviewer #1 (Remarks to the Author):

Van Lier and coworkers performed a randomized, placebo-controlled, double-blind, crossover study on the effects of 4-week clenbuterol treatment (40 μg/day) on tissue-specific insulin-stimulated glucose uptake in overweight and obese individuals at risk for type 2 diabetes mellitus (T2DM). Using 18F-FDG PET-MRI combined with hyperinsulinemic- euglycemic clamps, the authors demonstrated that clenbuterol enhanced insulin-stimulated glucose uptake in hamstring muscle and showed a trend toward improvement in vastus lateralis muscle. Clenbuterol did not affect brown adipose tissue (BAT) glucose uptake, whole-body insulin sensitivity, or fasting metabolic parameters. While these findings provide valuable translational evidence for β-adrenergic pathway modulation in glucose metabolism, several methodological and interpretive limitations impact the clinical significance of the results.

Major Comments

- The sample size is very small considering the heterogeneity of the population under investigation. The power calculation was based on an expected 20% improvement in glucose uptake, but the observed effects were smaller (13-15%). The Cohen's d values (0.282-0.441) indicate small to medium effect sizes, suggesting the study was underpowered for the actual effects observed. This raises questions about the clinical meaningfulness of the detected changes.

Our sample size calculation was based on a crossover design, where each participant underwent both conditions (placebo and clenbuterol). This design allows for within-subject comparisons, thereby increasing statistical power and reducing inter-individual variability. Consequently, both the sample size estimation and the analyses were based on a paired t-test, which accounts for within-subject correlations. We have clarified this in the Methods section.

Following this reviewer's comment of the reported Cohen’s d values, we noticed that our initial values were incorrect, unfortunately. When calculating with the correct values using SPSS 28, the effect sizes are d = 0.575 (mean difference= -1.468 and SD difference= 2.554) for the vastus lateralis and d = 0.649 (mean difference= -1.567 and SD difference= 2.416) for the hamstring muscle. These values indicate moderate to large effects, suggesting that the sample size was sufficient to detect the observed increase in skeletal muscle glucose uptake. The Cohen's values mentioned in the manuscript have been amended accordingly. For the clinical relevance of this mechanistic target or increasing muscle glucose uptake in general, we would like to refer to our answer on major comment #3 from this reviewer.

- The authors should provide a better rationale for choosing clenbuterol over more selective β2-agonists such as salbutamol or formoterol. Given the cardiovascular side effects observed and the known selectivity profiles of available β2-agonists, the choice of clenbuterol requires better justification, particularly in a population at risk for cardiovascular disease.

We thank the reviewer for pointing this out. The authors wish to emphasize that the aim of this study was not to advocate for repurposing clenbuterol as a therapy for type 2 diabetes mellitus (T2DM) or insulin resistance. The well-known side-effect profile of clenbuterol clearly prevents this. Instead, clenbuterol was used in the current study, as a tool compound to probe the metabolic effects of prolonged systemic β2-adrenergic receptor stimulation in overweight to obese individuals. We opted for clenbuterol over other β2-agonists such as salbutamol or formoterol, given its high β2-selectivity, oral bioavailability and long half-life (~35h), allowing specific and relatively stable systemic β2-agonist exposure over time (Vulić et al., *J. Anal. Toxicol*, 2014. PMID: 24661992; Yamamoto et al., *J. Pharmacobiodyn*, 1985. PMID: 4045696; Baker et al., *Br. J. Pharmacol,* 2010. PMID: 20590599). In response to the reviewer's comment, we have now emphasized the proof-of-concept nature of the study in the manuscript and explicitly added the rationale for the use of clenbuterol to the Methods section of the revised manuscript.

- The lack of improvement in whole-body insulin sensitivity, fasting glucose, or other systemic metabolic parameters raises questions about the clinical relevance of the muscle- specific improvements. The authors need to better address how localized muscle glucose uptake improvements without systemic benefits would translate to meaningful clinical outcomes in T2DM prevention or treatment.

We thank the reviewer for raising this important point. As mentioned above, our study was designed as a proof-of-concept to demonstrate that β_2-adrenergic stimulation can directly enhance skeletal muscle glucose uptake in humans with overweight and obesity, a population at increased risk of developing T2DM. Although, we did not observe improvements in systemic metabolic control, the increase in muscle glucose uptake observed in the current study is an encouraging signal. Since skeletal muscle accounts for the majority of post-prandial glucose uptake, enhancing skeletal muscle glucose uptake is a plausible approach to reduce glycaemia on the long-term. In this context, it should be noted that acute exercise also produces rapid increases in muscle glucose uptake that do not immediately translate to systemic metabolic benefits, such as lower fasting glucose or HbA1c. However, when exercise is sustained for a longer period (i.e. exercise training) glycaemic control is improved. Exercise training is also commonly adopted as first-line lifestyle intervention in people with or at risk for T2DM.

To achieve benefits on clinical endpoints, the duration (and dose) of β_2-adrenergic treatment likely matter. Obviously, clenbuterol itself is not suitable for (long-term) clinical studies in people with or at risk for T2DM due to its impact on hepatic glucose production and its cardiovascular side effects. However, a recent report highlighted the development of GRK2-biased β_2-agonists, able to selectively activate the intracellular GRK2 pathway, thereby enhancing skeletal muscle glucose uptake while limiting intracellular cAMP production and hence potentially minimizing associated metabolic (e.g. enhanced hepatic glucose output) and cardiovascular side effects (Motso et al. *Cell*, 2025; PMID: 40882626). Such compounds would allow safe (long-term) studies in people with or at risk for T2DM, probably also at higher doses. Such studies are warranted to establish the true clinical potential of this pathway. The current study

indicates that this clinical avenue is worth pursueing since we could show that the mechanistic target is active and functional in this population at risk for developing T2DM.

The following sentence(s)/section has been added to the discussion of the manuscript to reflect this point:

“Nevertheless, modest alterations in hepatic glucose output or possibly in adipose tissue glucose and lipid fluxes cannot be excluded and could have offset the beneficial effects on skeletal muscle MRgluc, resulting in unchanged whole-body insulin sensitivity. Despite the lack of changes in whole-body insulin sensitivity, the increase in muscle glucose uptake observed in the current study is an important finding, as skeletal muscle accounts for the majority of post-prandial glucose uptake (32), and enhancing skeletal muscle glucose uptake—for example, through acute exercise—is known to have beneficial health effects. However, longer-term studies will be needed to investigate whether β2-adrenergic stimulation of muscle glucose uptake results in clinical benefits on the long term.”

“Obviously, clenbuterol itself is not suitable for long-term treatment or clinical studies in people with or at risk for T2DM due to its cardiovascular side effects, which is why we also excluded these patients from the current study and focused on relatively healthy overweight to obese individuals.”

“In this context, a recent report highlighted the development of GRK2-biased β_2-agonists, able to selectively activate the intracellular GRK2 pathway, thereby enhancing skeletal muscle glucose uptake while limiting intracellular cAMP production and hence potentially minimizing associated metabolic (e.g. enhanced hepatic glucose output) and cardiovascular side effects (Motso et al. Cell, 2025; PMID: 40882626). Such compounds would allow safe (long-term) studies in people with or at risk for T2DM - probably also at higher doses - warranted to establish the true clinical potential of this pathway.”

Minor Comments

- The exclusion of T2DM individuals should be mentioned in the "Study participants" section rather than first appearing in the discussion.

We have now added this information to the method section entitled “Study participants”.

- The manuscript should specify how many participants fell into overweight versus Class I obesity categories and whether outcomes differed by BMI subgroup.

Unfortunately, our study was not powered to allow analyses per BMI sub-group. The number of overweight (n=10) versus class I obese (n=4) individuals included in the study is now mentioned in Table 1 of the Result section of the manuscript, as requested.

- With only 3 females out of 14 participants, the generalizability of results is limited.

The current study was primarily designed to assess within-individual differences in muscle-specific glucose uptake upon placebo vs. β_2-agonist treatment, rather than to compare sex differences. Nonetheless, we acknowledge that having only three females (out of 14 participants) in the study sample may limit the generalizability of our findings and mention this as a limitation in the discussion section of the manuscript:

"Secondly, our study included fewer females than males, which may reduce the generalizability of the results, as sex-specific differences in glucose metabolism (36) and/or β2-AR agonist stimulation (37, 38) have been observed."

- All blood parameter values in Table 1 are reported without corresponding units.

We have added the units in Table 1 of the Results section.

- "Kalium" should be replaced with "Potassium" and "Hemoglobin" is misspelled.

We have corrected the misspelled terms in Table 1 of the Results section.

- Plasma glucose values could be added to Table 1 if measured during screening.

Plasma glucose values were not measured during screening, and could therefore not be reported in Table 1, unfortunately.

- The relevance of measuring insulin's suppressive effect on fatty acids should be better explained.

Given the inhibitory action of insulin on lipolysis, the measurement of insulin-induced suppression of plasma free fatty acids (FFA) provides an index of adipose tissue insulin sensitivity, complementing the assessment of insulin-stimulated glucose uptake. In our study, the absence of change in FFA suppression indicates that adipose tissue insulin sensitivity was not affected by the intervention. We provided additional explanation of the relevance of this measurement in the Result section.

- The authors should better address the apparent paradox of how β2-agonists could have therapeutic utility in T2DM treatment if they increase hepatic glucose production without changing fasting plasma glucose. This relates to the need for more selective β2-agonist profiles or weight-based dosing strategies.

Indeed, the observed increase in skeletal muscle glucose uptake, as directly measured by ^{18}F-FDG PET-MRI, is likely parallelled by an increased hepatic glucose output upon clenbuterol treatment, resulting in unchanged fasting glucose levels. A similar effect has been observed with SGTL2i, which also leads to a compensatory increase in hepatic glucose production (Merovci et al. *J Clin Invest*. 2014). However, as indicated above (major comment #3 of this reviewer), the observed increase in skeletal muscle glucose uptake upon clenbuterol does show that the mechanistic target is active and functional in a population at risk for developing T2DM. For the therapeutic utility of this mechanistic target or for increasing muscle glucose uptake in general, we would like to refer to our answer on major comment #3 from this reviewer.

Reviewer #2 (Remarks to the Author):

This study has examined the effects of a 4-week treatment with the β2-adrenergic agonist clenbuterol (40 μg/day) on insulin-stimulated metabolic rate of glucose (MRgluc) in the quadriceps muscle (primary outcome) and glucose uptake in brown adipose tissue (BAT) (secondary outcome) using 18F-FDG PET-MRI in fourteen individuals with overweight or obesity. The authors conclude that insulin-stimulated MRgluc tended to improve in vastus lateralis muscle (15%, p=0.072) and increased significantly in hamstring muscle (13%, p=0.037), while BAT glucose uptake remained unaffected and that these findings suggest potential therapeutic benefits of β2-AR agonists for improving skeletal muscle glucose uptake in individuals with (pre)diabetes.

In general this is a well conducted comprehensive clinical study by an experienced group using state-of-the-art methodology. I do however have major concerns as regards the design of the study and the interpretation of data.

It appears that clenbuterol was given twice daily together with morning and evening meals, the final dose being taken at 18.00 prior to the study day; this effectively implies that this by design is a post intervention study, which disregards and is not representative of metabolic phenomena occurring the previous 4 wks during active β-2 treatment. It is the metabolic events during treatment, which determines the suitability of the treatment, not withdrawal repercussions and it is generally well documented that other β2-adrenergic agonists like adrenaline are potent insulin antagonists glucose and lipid-wise and induces insulin resistance acutely – one study has reported that insulin stimulated glucose uptake in the adrenaline perfused human leg(including the vastus lateralis) acutely decreases > 80 % by A/V techniques(Gjedsted et al Acta Physiol, 2011 (& J Onslev et al, J Clin Endocrinol Metab. 2024)) and it is well described that adrenaline is a crucial component of the counterregulatory cascade and that up to 50% of patients with pheochromocytoma develop diabetes. Thus, if anything one would in parallel with all other stress hormones expect intermittent hyperglycemia and compensatory hyperinsulinemia during the active 4 wk treatment - continuous glucose – and lactate - monitoring or perhaps HbA1c would have been useful in this context. Another possibility is that 4 wks of active treatment inhibits endogenous catecholamine generation and tentatively induces refractory adrenal atrophy and low levels of endogenous catecolamines after intervention – it might be considered to measure urinary catecholamine excretion post-intervention. Given the cardiometabolic side effects of β-2 treatment and the vulnerability of patients with pre-diabetes/metabolic syndrome, most clinicians would be much reluctant to use such a remedy.

We thank the reviewer for his/her feedback and for raising these important points. As mentioned in our response to reviewer 1, we wish to emphasize that the aim of this study was not to advocate for repurposing clenbuterol as a therapy for type 2 diabetes mellitus (T2DM) or insulin resistance. The side effects of clenbuterol clearly preclude such repurposing. Instead, this was a proof-of-principle study to determine whether selective β_2-adrenergic receptor stimulation can improve skeletal muscle glucose uptake in overweight/obese humans, to confirm this pathway as a clinical target and to guide the development of novel, safer β-agonists.

Given the long (~35h) half-life of clenbuterol, no rebound or withdrawal effects are anticipated after the last dose, taken during the evening prior to the study day. This design, continuing treatment up to and including the day prior to the study day, was considered the most appropriate to assess chronic, steady-state metabolic adaptations to prolonged β_2-adrenergic stimulation.

The reviewer correctly points out that acute or short-term β-adrenergic stimulation can elevate plasma glucose and insulin levels. However, it has previously been demonstrated that these pro-diabetogenic effects normalize and even reverse into anti-diabetogenic effects upon prolonged β_2-agonist exposure (Kalinovich et al, *Diabetologia*, 2020. PMID: 32472192). Various preclinical (Elayan et al. *Cell Mol Neurobiol*, 2012., Meister et al. *Nat Comm*. 2022. PMID: 35013148) and clinical studies (van Beek et al. *Nat Comm*, 2023, PMID: 36635304, Jessen et al. *J Physiol*, 2022. PMID: 35218559) have hitherto highlighted the anti-diabetogenic effects of (prolonged) β_2-agonist exposure. Regrettably, we did not perform continuous glucose (or lactate) monitoring in the current study. Since HbA1C assessment requires a whole-blood sample and reliable catecholamine analysis requires specific pre-analytical handling prior to freezing, that were not available or applied in the current study, these analyses could not be performed either. However, to address the reviewer's comment related to potential refractory adrenal atrophy upon clenbuterol treatment, we determined adrenal gland volume using T2-weighted images acquired during PET-MRI scanning, which were used for anatomical correlation (***Fig S1a and S1b***). After 4 weeks of clenbuterol treatment however, no significant differences were observed between clenbuterol and placebo, neither in the right adrenal gland volume ($p = 0.752$) nor in the left ($p = 0.747$) and mean adrenal gland volume (average of left and right adrenal glands; $p = 0.963$) (Results section of the manuscript and ***Fig S1c-e***).

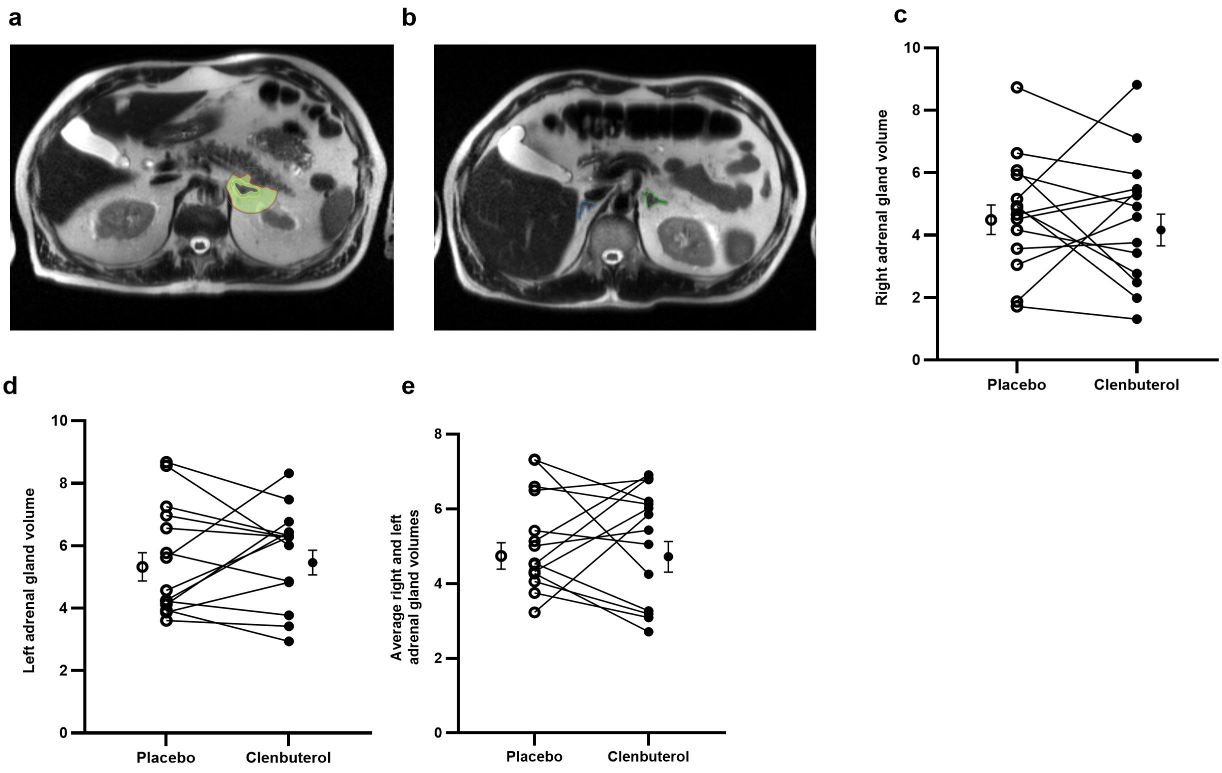

***Figure S1. Adrenal gland volume was not different after four weeks of clenbuterol.** A manual contour was drawn widely over the adrenal gland (yellow contour). Thereafter with a threshold tool based on intensity differences, the adipose tissue was segmented in the region of the adrenal gland (Red contour). The Adipose tissue contour was subtracted from the manually drawn contour of the adrenal gland, resulting in a more precise contour of the adrenal gland (green contour), using MIMvista software (a), The final segmentation consisted of the segmentation of the right adrenal gland (blue) and left adrenal gland (green) (b). All data are presented as mean ± SEM and statistically analyzed with paired sample t-test. n=13 for all figures.*

M-values are clearly the most important measure of WB insulin sensitivity and should be given in the abstract & numerically elsewhere – the conclusions seem a bit overstretched – if there is a minor improvement of insulin sensitivity in some muscles, there must by implication be deterioration in other tissues – where?

We thank the reviewer for this valuable comment. We have now added the M-value to the Abstract, as requested. We wish to emphasize though, that the M-value was an exploratory outcome in our study, whereas the primary endpoint was insulin-stimulated skeletal muscle glucose uptake assessed by ^{18}F-FDG PET, and brown adipose tissue (BAT) glucose uptake was a predefined secondary outcome.

We agree with the reviewer that the observed increase in insulin-stimulated glucose uptake in skeletal muscle, is likely parallelled by opposing effects elsewhere. For example, modest alterations in hepatic glucose output or adipose tissue glucose/lipid fluxes cannot be excluded and could offset the beneficial effects in skeletal muscle,

resulting in unchanged M-values. Although we did not observe improvements in systemic metabolic control, we believe that the increase in muscle glucose uptake observed in the current study nonetheless bears clinical relevance, as further elaborated on in response to Reviewer 1, major comment #3.

In response to this reviewer's comment, we refined the Discussion section to reflect that clenbuterol increased insulin-stimulated skeletal muscle glucose uptake without affecting whole-body insulin sensitivity, consistent with its localized rather than systemic action under the current conditions.

Target plasma glucose values during the clamp were high (6.0 - 6.5 mmol/L) – did the authors measure c-peptide during the clamp?

The relatively high target plasma glucose values (6.0–6.5 mmol/L) were deliberately chosen to ensure participant safety during PET imaging, as hypoglycemia can pose a substantial risk under these experimental conditions. Guided by the reviewer's comment, we have now determined C-peptide during the hyperinsulinemic-euglycemic clamp, to estimate endogenous insulin secretion under these conditions. These new data show that c-peptide levels were similar between placebo and clenbuterol treatment (1.71 ± 0.23 nmol/L vs. 1.45 ± 0.17 nmol/L, respectively; p= 0.103), indicating that clenbuterol did not significantly affect endogenous insulin secretion during the clamp. These data have now been added to the Results section of the manucript.

Reviewer #3 (Remarks to the Author): General Comments

This manuscript reports a crossover trial examining whether β2-adrenergic receptor stimulation improves tissue-specific glucose clearance in skeletal muscle and brown adipose tissue (BAT), and whether this augments insulin sensitivity in individuals at risk of developing type 2 diabetes mellitus (T2DM). The research question is interesting and relevant; however, several methodological and analytical issues require clarification.

- Sample Size Calculation:

  While the reported sample size may be sufficient, the rationale for using a paired t-test to estimate the required sample size is unclear. In a crossover design, the primary goal is typically to compare differences between treatment and control conditions rather than only within-subject changes. In much of the methodological literature, sample size estimation for crossover trials incorporates elements more consistent with independent samples t-tests [1,2] or online calculator https://www2.ccrb.cuhk.edu.hk/stat/mean/tsmc_sup.htm

  Furthermore, if the authors assumed a mean difference (d) of 0.0142 with an SD of 0.0037, the implied effect size would be approximately 3.8, which would lead to an even smaller sample size than reported. I attempted a calculation using the pwr package in R, which supported this observation. The authors should clarify their assumptions and provide more detail on the sample size estimation approach.

  [1] Wellek, S., & Blettner, M. (2012). On the proper use of the crossover design in clinical trials: part 18 of a series on evaluation of scientific publications. Deutsches Arzteblatt international, 109(15), 276–281. https://doi.org/10.3238/arztebl.2012.0276

  [2] Jones, B., & Kenward, M. G. (2003). Design and analysis of cross-over trials (2nd ed., Vol. 98;98.;). Chapman & Hall/CRC. [Chapter 2 and 5]

  In our crossover design, each participant undergoes both conditions (placebo and clenbuterol), which means that treatment effects are evaluated within the same individuals. Consequently, the appropriate statistical approach for both analysis and sample size estimation is a paired t-test, which accounts for the within-subject correlation between repeated measures rather than treating the two conditions as independent samples. The sample size of 14 participants was based on published reproducibility data for skeletal muscle glucose uptake in the thigh measured by ^{18}F-FDG PET (Johansson et al. Radiology. 2018, PMID: 28846496). The reviewer may have missed that the value of 0.0142 mL/cm3/min does not reflect the mean difference, but rather the mean ^{18}F-FDG uptake in the thigh muscle, with a standard deviation of 0.0037 mL/cm3/min. Since we anticipated an intervention effect of 20%, the mean average difference (0.0142 x 1.2-0.0142= 0.00284 mL/cm3/min) was used in the formula, rendering a sample size of 13.4, which equals 14 subjects.

Formula (sample size N per sequence):

$N = \sigma^2 * (Z\_\alpha/2 + Z\_\beta)^2 / 2\,\delta^2 = 0.0037^2 * (1.96 + 0.84)^2 / (2 * 0.00284^2) = 1.0733*10^{-4} / 1.61312*10^{-5} = 6.7$ → 7 per sequence (Clenbuterol followed by placebo or vice versa)

N = 14 in total.

The description of the sample size estimation approach in the Methods section has been refined in the revised version of the manuscript.

- Carryover Effect:

  Although the study included a 6–8 week washout period, it would be important to report whether potential carryover effects were formally assessed or accounted. This is a key concern in crossover trials and should be addressed explicitly.

  We thank the reviewer for raising this important point. In crossover studies, carry-over effects are a potential concern. To minimize this risk, we implemented a washout period of 6–8 weeks. Given the half-life of clenbuterol (~35 hours), approximately 97% of the drug is eliminated after 5 half-lives (~7 days) and >99% after 7 half-lives (~10 days). Our implemented washout period of 6–8 weeks (42–56 days) is therefore substantially longer than required to completely eliminate the drug.

  However, as physiological outcomes could potentially outlast drug availability, we analyzed the change in metabolic rate of glucose uptake in vastus lateralis (primary outcome) between the clenbuterol and placebo arms and compared this change across the two randomization sequences (clenbuterol - placebo; n=6, meanΔ=1.437, SD= 2.176, and placebo - clenbuterol; n=6, meanΔ=1.499, SD= 3.100, two participants were excluded from the analysis due to inadequate image quality for the primary outcome, n=12). Although group sizes were small, no significant differences (p= 0.989, two tailed independent sample t-test) were observed between both sequences. This analysis, together with the long washout period, indicate that carry-over or order effects are unlikely to have influenced the study outcomes. The Methods section has been updated to reflect this point.

Statistical Analysis

The data analysis section is currently very brief and states only that paired t-tests were used for normally distributed variables and Wilcoxon signed-rank tests for non-normal variables. As noted above, it is not clear whether a paired t-test is the most appropriate method for the primary analyses. More detail should be provided on the planned statistical approach, including whether more robust methods such as mixed-effects models (treating subjects as random effects) were considered. Such approaches are frequently recommended in crossover designs [2].

[2] Jones, B., & Kenward, M. G. (2003). Design and analysis of cross-over trials (2nd ed., Vol. 98;98.;). Chapman & Hall/CRC. [Chapter 2 and 5]

We acknowledge that mixed-effects models, which treat subjects as random effects, are frequently recommended for crossover trials, particularly for outcomes measured repeatedly within a period (e.g., week 0, week 2, week 4). In our study, the primary endpoint was measured only at the end of each treatment period. Mixed-effects models could additionally offer advantages in handling missing data. While only a few participants had missing data (hamstring: n = 13; vastus lateralis: n = 12) and the outcome was only measured at week 4, the advantages of the mixed-effects model approach would be limited. Importantly, a key benefit of the paired t-test in this context is that all statistical power is directed toward estimating the within-subject intervention effect. Because no additional parameters are estimated—as would be the case in mixed-effects models—the analysis is more stable, particularly with our sample size. Furthermore, since the sample size of our study was calculated based on a paired t-test, and the primary analyses were also predefined in the research protocol, we preferred this less complex method for analyzing our data. We feel this approach is appropriate given our study design and the nature of the primary outcome (change in skeletal muscle glucose disposal at the end of each treatment period).

- Results:

In the results section, I noted descriptions such as: "... although this did not reach statistical significance (11.43 ± 0.97 vs. 9.96 ± 0.96 μmol/ min/100g, p=0.072, Cohen's d = 0.441; Fig 1a and b)..."

The reporting here suggests the use of independent samples t-tests, which seems inconsistent with the stated use of paired tests. The authors should clarify exactly which statistical tests were performed for each analysis and ensure alignment between the methods and results sections.

I think the results are clearly presented.

We thank the reviewer for this comment and the positive feedback on the clarity of our results. All statistical comparisons, including the example cited, were performed using paired tests, which has been stated in the Methods section and is additionally indicated in our figure legends. However, we adapted the reported format for the primary and secondary research questions to clarify that these were results of the paired comparisons between clenbuterol and placebo, not independent samples: 11.43 ± 0.97 vs. 9.96 ± 0.96 μmol/min/100g, **mean difference in paired sample t-test= 1.47, 95%CI -0.15, 3.09**, p=0.072, Cohen's d = -0.575; Fig 1a and b.

REVIEWERS' COMMENTS

Reviewer #2 (Remarks to the Author):

The ms has improved considerably - I still believe that it is important to underline that the design implies that the investigation is carried out in a transitional phase in which drug levels are declining and that generalizability is dubious.

Thank you, we have now included this as a limitation in the Discussion section of the manuscript, as follows: " Secondly, given the long (~35h) half-life of clenbuterol, rebound or withdrawal effects following the last dose taken in the evening prior to the study day are likely limited, but cannot be fully excluded and may influence the observed metabolic responses."

Reviewer #3 (Remarks to the Author):

The authors have addressed all my questions. I don't have further comments.

We thank the reviewer for the careful reassessment of our revised manuscript and for confirming that the previous comments have been satisfactorily addressed.

Reviewer #4 (Remarks to the Author):

Review Prolonged β2-agonist treatment enhances muscle-specific glucose uptake in individuals with overweight and obesity: a randomized placebo-controlled trial

This is a well conducted and timely mechanistic clinical trial investigating the effect of beta2-AR agonist clenbuterol on muscle glucose uptake. It uses state-of-the-art technology. The conclusions drawn by the authors are supported by the presented data.

As tasked I am reviewing the answers to the requests of **Reviewer 1** who was not able to review the revised manuscript.

Major points:
Sample size and power.
- I agree with the authors that their sample size was adequate. Importantly, it should be noted that the project was designed to elucidate basic physiological / pharmacological mechanisms and not to re-purpose clenbuterol as a new diabetes medication. Furthermore, the question whether the power was too low would only be of relevance when

no significant difference between active treatment and placebo was observed.

- choice of clenbuterol as beta2-agonists.
the authors satisfactorily justify their choice of medication. It should be highlighted that in this kind of investigator-initiated trials rely on the availability of medications. I.e. usually medications that are already approved and marketed have to be used.

- clinical relevance of the study
As commented above, the trial presented here was intended to elucidate basic mechanisms in humans and to guide further research. It was not a clinical trial intended to obtain approval for a new treatment indication. As such, Nature Communications is also a very suitable journal to present these data. The authors have addressed the reviewer's comment satisfactorily.

Minor points
- the authors have satisfactorily answered all minor points raised by the reviewer.

We thank the reviewer for taking the time to review our answers to the comments raised by reviewer 1, who was not available at this time. The reviewer is satisfied with our responses to Reviewer 1’s comments and does not raise any additional points that require a response.
